# How addressing menstrual health and hygiene may enable progress across the Sustainable Development Goals

**DOI:** 10.1080/16549716.2021.1920315

**Published:** 2021-06-30

**Authors:** Marni Sommer, Belen Torondel, Julie Hennegan, Penelope A. Phillips-Howard, Thérèse Mahon, Albert Motivans, Garazi Zulaika, Caitlin Gruer, Jacquelyn Haver, Bethany A. Caruso, Monitoring Menstrual Health and Hygiene Group

**Affiliations:** a Department of Sociomedical Sciences, Mailman School of Public Health, Columbia University, New York, NY, USA; b Department of Disease Control, London School of Hygiene and Tropical Medicine, London, UK; c Maternal, Child and Adolescent Health Program, Burnet Institute, Melbourne, Australia; d Melbourne School of Population and Global Health, University of Melbourne, Melbourne, Australia; e Department of Clinical Sciences, Liverpool School of Tropical Medicine, Liverpool, UK; f WaterAid, London, UK; g Equal Measures 2030, Plan International, London, UK; h Department of Sociomedical Sciences, Mailman School of Public Health, Columbia University, New York, USA; i Save the Children US, Washington, DC, USA; j Hubert Department of Global Health, Rollins School of Public Health, Emory University, Atlanta, USA; k Multiple Institutions; End of Manuscript and Appendix B; Monitoring Menstrual Health and Hygiene Management, Green Paper

**Keywords:** Menstrual health, gender, education, sanitation, psychosocial

## Abstract

There is increasing global attention to the importance of menstrual health and hygiene (MHH) for the lives of those who menstruate and gender equality. Yet, the global development community, which focuses on issues ranging from gender to climate change to health, is overdue to draw attention to how addressing MHH may enable progress in attaining the Sustainable Development Goals (SDGs). To address this gap, we undertook a collective exercise to hypothesize the linkages between MHH and the 17 SDGs, and to identify how MHH contributes to priority outcome measures within key sectoral areas of relevance to menstruating girls in low- and middle-income countries. These areas included Education, Gender, Health (Sexual and Reproductive Health; Psychosocial Wellbeing), and Water, Sanitation and Hygiene (WASH). These efforts were undertaken from February – March 2019 by global monitoring experts, together with select representatives from research institutions, non-governmental organizations, and governments (*n* = 26 measures task force members). Through this paper we highlight the findings of our activities. First, we outline the existing or potential linkages between MHH and all of the SDGs. Second, we report the identified priority outcomes related to MHH for key sectors to monitor. By identifying the potential contribution of MHH towards achieving the SDGs and highlighting the ways in which MHH can be monitored within these goals, we aim to advance recognition of the fundamental role of MHH in the development efforts of countries around the world.

## Background

The Sustainable Development Goals (SDGs) are a collective agenda that identifies how the global society can enable sustainable economic, social, and environmental development for all, with an emphasis in the preamble to the SDGs on the need ‘to achieve gender equality and the empowerment of all women and girls [1]. Despite the emphasis on gender, the SDGs do not explicitly address the natural biological occurrence of menstruation, something experienced by almost two billion people globally, or its effects on the health and development agenda [1]. There is an overdue need to assess the importance of menstrual health and hygiene (MHH) towards enabling progress across the entirety of the 17 SDGs, and highlight the sectors that would benefit from addressing menstruation when seeking to achieve their own goal-specific outcomes [2].

The onset of menstruation, menarche, is a natural and important aspect of a female’s physiological development [3–5]. From menarche through to menopause, people who menstruate experience the necessity of regularly managing menstruation: collecting, removing, and cleaning menstrual blood from the body; contraceptives that may disrupt menstrual bleeding; and for some, the experience of debilitating menstrual discomfort or disorders, for which, awareness, diagnosis and treatment is still lacking. In most societies, menstruation is laden with societal taboos and secrecy, which can hinder the ability to manage menstruation with ease and confidence [6]. Menarche may also bring expectations about roles, responsibilities, and behaviors, in some cases serving as a ‘signal’ of sexual or marital readiness [7,8] and intensifying the gendered experiences of girls, women, and all people who menstruate [9,10].

The term ‘menstrual health and hygiene (MHH)’ is used to describe the needs experienced by people who menstruate, including having safe and easy access to the information, supplies, and infrastructure needed to manage their periods with dignity and comfort (menstrual hygiene management) [11] as well as the systemic factors that link menstruation with health, gender equality, empowerment, and beyond [12].

We posit that to fully achieve the SDGs by 2030, it is necessary to recognize MHH as a contributing factor to a broad set of SDGs. In this paper, we outline the potential linkages between MHH and all of the SDGs. Furthermore, to ensure MHH is adequately considered, we highlight the range of outcomes to which unmet menstrual needs are relevant, as well as specific outcomes prioritized within sectors closely aligned with MHH, with a main focus on girls in and out of school from low- and middle-income countries.

## A framework linking MHH with SDGs

**Figure 1.**
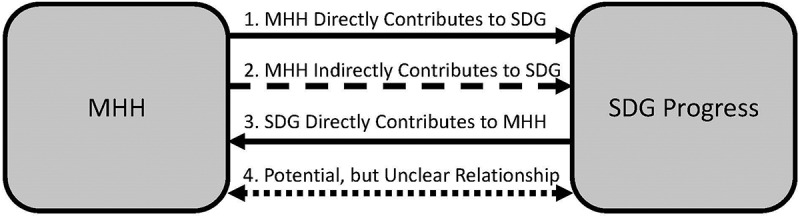
Relationship between menstrual health and hygiene (MHH) and Sustainable Development Goals (SDGs)

In highlighting the relevance of MHH across a broad set of global priorities, we suggest that MHH may align with the SDGs in the following ways: (1) MHH directly contributes to achieving a given SDG, but its role has not been recognized or directly evaluated; (2) MHH contributes to achieving a given SDG through clear indirect pathways, suggesting a value to prioritizing attention; (3) MHH is influenced by progress towards a given SDG, and may serve as a ‘proxy’ for gender-equitable progress; and (4) potential but unclear relationship between SDG and MHH (see Figure 1).

Linkages between the SDGs and MHH can be made evident by questioning how the overall achievement of a given SDG may be altered if MHH is not addressed. For example, in examining SDG3, which focuses on health, is it possible to achieve health and wellbeing for all if MHH practices, knowledge, access to healthcare, and support are not adequate or available? If not, the global community must consider the policy levers available to address this gap. Or, in examining SDG5, is it possible to achieve gender equality of schools, workplaces and even households if they do not provide social and physical environments enabling safe, hygienic, and comfortable management of menstruation? If not, governments must consider resource investments to address this need.

The overall aim of this paper is to raise awareness of MHH as a contributing factor to a broader array of SDGs. Two specific aims are to:

- Describe the SDGs that are most closely aligned with MHH and the type of linkages between them; and
- Highlight priority outcomes for sectors to monitor MHH directly, or to use as mechanisms for long-term tracking of MHH as a useful proxy measure for the advancement of a broader array of SDGs.

## Rationale for linking MHH with the SDGs and monitoring MHH across sectors

Over the last decade there has been significant growth in attention to MHH, and more investment to evaluate if programs or policies have the desired impact, particularly in relation to girls in and out of school in low- and middle-income countries [13–15]. However, despite this significant growth, MHH remains underacknowledged and underfunded, frequently dismissed as an extra, rather than essential consideration for development. The topic risks continued marginalization without explicit recognition of its role across development objectives. Furthermore, there are no consistent or agreed upon means of monitoring MHH to assess change and progress over time [16]. Identifying linkages between MHH and the SDGs could help to establish monitoring efforts. Linking with the SDGs not only provides an opportunity to demonstrate how interconnected MHH is with other globally recognized priorities but may also facilitate the development of indicators and measures that could be integrated into systems already created for tracking each of the linked goals. If explicit linkages between MHH and the SDGs are not made clear and monitored, MHH may continue to be ignored or considered ‘outside the scope’ of various sectors and their priorities. Therefore, identifying these explicit linkages is critical to making sure MHH is appropriately acknowledged and addressed.

It is important to highlight that menstruation has been assumed to be linked to SDG 6.2, which calls for ‘paying special attention to the needs of women and girls’ in relation to sanitation and hygiene. While linkages with SDG 6.2 are clear, menstruation is not considered in any of the indicators to monitor SDG 6.2, which itself is considered gender blind [17].

## Methodological approach

To support future efforts to explore and more directly address the linkages between MHH and the SDGs, we undertook a consultation process to highlight key linkages and priority outcomes attentive to a broader range of SDGs, with a main focus on girls in and out of school in low- and middle-income countries. International experts representing key sectors [Health (sexual and reproductive; psychosocial wellbeing), Education, Gender, WASH] for girls came together to identify outcomes within their respective sectors to be prioritized by the following: (1) their hypothesized relation to MHH and (2) importance to achieving the SDGs. The process included a desk review and compilation of existing MHH indicators and priorities led by a Scientific Technical Advisory Group (STAG) (*n* = 7); the engagement of a Global Advisory Group (GAG) (*n* = 38) to feed into the analysis and its outputs; and a ‘Monitoring Menstruation’ task force meeting of sectoral measurement experts (*n* = 26). Activities were based on the aspirational goal: ‘Girls live in societies that enable them to be confident and knowledgeable about their menstruation and able to manage it with dignity, safety, and comfort, thereby promoting their health, wellbeing, and ability to realize their potential and equitable role in society’ [18].

**Figure 2.**
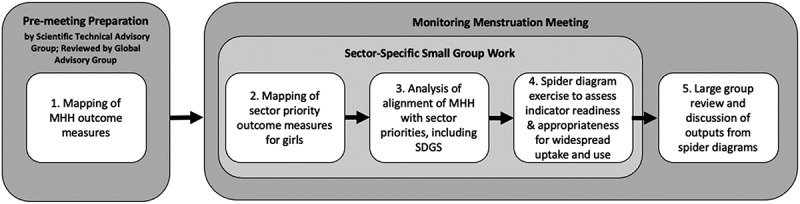
Methodological approach for identifying priority outcomes

The specific methodological approach for identifying priorities among select sectors (see Figure 2; see *supplemental files*) included the following: [a] mapping of MHH outcome measures recognized to be of importance for girls, and of priority outcome measures for girls within key sectoral areas; [b] analysis of how addressing MHH aligns with identified key sectoral priorities, including attention to the SDGs; and [c] development of spider diagrams focused on the top three identified MHH-aligned outcome measures per relevant key sector. The spider diagram analyses assessed the readiness and appropriateness of identified outcome indicators for widespread uptake and use. The terminology being utilized refers to the following: goals describe the overall objective; targets provide a numerical value for achieving the goal; indicators are summary measures that attempt to estimate the status of a given target or goal; input measures assess the resources available for a given program or intervention; and outcome measures are used to capture short or medium-term change in a given population [19]

After completing Steps 1–3 (see Figure 2), the priority outcomes identified by experts from each represented sector (Gender, Education, Health, WASH) were found to align with MHH in multiple ways for girls in and out of school in low- and middle-income countries [18].

## SDGs aligned with MHH

As depicted in Table 1 (generated from content discussed in Steps 2–5 above), multiple SDGs were identified as relevant to MHH. While we will not reiterate the content of the table, we will highlight some key linkages. For example, in addition to the provision of water, sanitation and hygiene (WASH) facilities, which SDG 6 does capture, materials to deal with menstrual flow are influenced by poverty and inequality (e.g. SDG1, no poverty). The need for adequate disposal systems for menstrual material waste has important implications for the larger environment (SDG 15), and for rural and urban planners in regard to basic services, including gendered sanitation (SDG 11).

**Table 1.** Relevance of Menstrual Health and Hygiene (MHH) to the Sustainable Development Goals (SDGs) and type of linkage (see Figure 1)

SDG	Hypothesized linkage to MHH	Type of link
		1. MHH directly contributes to SDG 2. MHH indirectly contributes to SDG 3. SDG directly contributes to MHH 4. Potential but unclear and/or undocumented relationship between SDG and MHH
**#1 No Poverty**	Lack of WASH, menstrual supplies/products, and pain management solutions may contribute to perpetuating the cycle of poverty as it limits the ability for those who menstruate to take part in economic opportunities.	2 and 3
	Poverty may contribute to lack of adequate WASH and menstrual supplies and products.	2 and 3
**#2 Zero Hunger**	Those who menstruate in poverty may need to make trade-offs (with food purchasing and other survival necessities) in daily life for menstrual materials.	3
	Restrictions on girls’ and women’s movements during menstruation may hinder the ability to engage in food procurement or food preparation-related activities.	3
**#3 Good Health and Well-Being**	Knowledge of the menstrual cycle and confidence navigating menstruation may have strong links with SRH and wellbeing, including family planning.	1
	Understanding menstrual pain and norms of menstrual bleeding may be essential for wellbeing, and for identifying whether or not there is a need to seek out advice or medical attention.	1
	Being able to hygienically manage menses may have consequences for reproductive health, reproductive tract infections (impacting maternal and HIV-related outcomes), and wellbeing.	1
	Access to health care and menstrual pain relief may influence MHH (e.g. universal access to SRH health-care services; integration of RH into national strategies)	1 and 3
	Positive associations may exist between MHH, SRH and mental health and psychosocial well-being; linkages, for example, with improved education/#4 and economics #8).	1
**#4 Quality Education**	School physical (e,g. toilets by sex with privacy, see SDG#6) and social environments that are not discriminatory towards menstruating students may improve MHH; includes disposal systems, such as SDG#6 and #9).	1 and 3
	Quality of teaching about MHH may strengthen knowledge and confidence in school for students who menstruate; Providing MHH training to teachers may increase support for menstruating students and improve their MHH.	1 and 3
	Presence of female teachers may increase social and more broadly education system support around MHH in school.	1 and 3
	Schools may serve as a location of MHH interventions, especially related to information, knowledge, life skills	1 and 3
	MHH experience may shape learning experiences; a lack of MHH knowledge, access to facilities and supplies, and health support may impede learning.	1 and 3
	Comprehensive Sexuality Education (CSE) supports knowledge and awareness mentioned in SDG3.	1 and 3
**#5 Gender Equality**	Assuring that all schools, workplaces, households, etc., may enable managing menstruation with dignity and comfort, challenge discriminatory gender norms and reduce experiences of stigma and gender inequitable social and physical environments.	1 and 3
	Poor MHH may be a source of gender inequality and discrimination, hindering the health and well-being of those who menstruate, and their engagement in activities of daily living, free of fear, shame and stigma.	1
	Engaging men and boys on menstruation in order to increase their knowledge and understanding, may reduce stigma and increase their support of those who menstruate in their lives.	1 and 3
**#6 Clean Water and Sanitation**	May enable the ability to manage menstruation in safety and comfort with facilities, clean water, disposal, etc. at home, schools, institutions, in public places, reducing gender discrimination of inadequate facilities.	1 and 3
	Access/right to water, sanitation and hygiene during menstruation may be essential for daily life.	3
	Menstrual waste may impact the sustainability and functionality of WASH services.	1 and 3
	Privacy and safety of WASH may be essential to protect against gender and sexual violence.	3
**#7 Affordable and Clean Energy**	Energy may be needed to produce single-use pads.	3 and 4
	Energy may be needed in maintaining (washing, boiling) menstrual products; ranging from menstrual pads to cloths to cups.	3 and 4
	Energy may be needed to adequately dispose of used menstrual products (e.g. burning); the latter also contributes to pollution.	3 and 4
**#8 Decent Work and Economic Growth**	Workplace physical and social environments may need to support MHH in order to not be gender discriminatory.	1 and 4
	Inadequate awareness of and resources for menstrual disorders may hinder engagement in work	1 and 4
	Reduced economic productivity may occur when MHH needs are not fully met.	1 and 4
	Ensuring access to safe and ggender-sensitivesanitation facilities and disposal solutions in work environments, the absence of which may hinder those who are menstruating from high economic productivity.	1 and 4
**#9 Industry, Innovation and Infrastructure**	Encouraging innovation in toilet design, disposal systems, laundering and menstrual product types may improve MHH.	2
	Small companies and social entrepreneurs making menstrual products and creating small sanitation-related businesses (female friendly toilets, waste disposal) may be generating employment	2
	May support environmentally responsible production of MHH products.	2
**#10 Reduced Inequalities**	May address inequalities in who can manage menstruation without significant barriers (e.g. people with disabilities, transgender individuals, those with unstable housing).	1
	Economic inequalities contribute to unmet MHH needs, and unmet MHH needs may exacerbate inequalities based on access to resources including products and changing/bathing (WASH) facilities, exposing girls and women to SRH risks and violence.	1
**#11 Sustainable Cities and Communities**	Providing female friendly toilets may enable good MHH.	1 and 2
	Constructing or adapting sanitation systems may adequately address product disposal and waste management.	1 and 2
	May ensure water availability and access for cleaning and laundering menstrual products as needed.	1 and 2
	May address pollution generated by single-use disposables and used reusables; dumping and burning of waste (plastics, fluoxins, dioxins).	1 and 2
**#12 Responsible Consumption and Production**	May support innovation on menstrual product waste management to reduce pollution.	2 and 3
	May provide menstrual product choice, including bio-degradable and safe reusable options.	2 and 3
**#13 Climate Action**	Water shortages secondary to climate change may impact MHH.	2 and 3
	Climate change-related planning and management may be insufficient if does not include MHH considerations for women, youth, and marginalized communities in relation to product innovations.	2 and 3
	May address pollution related to menstrual product disposal practices.	2 and 3
	Education of girls is a high-impact climate solution, which may be hindered if inadequate attention given to MHH in schools (see #4).	2 and 3
**#14 Life Below Water**	Single-use menstrual materials may contribute to environmental pollution.	2
	Lack of appropriate menstrual product disposal options may lead to disposal practices contaminating rivers, lakes and other waterways.	2
**#15 Life on Land**	Improper menstrual product disposal (and increased use of ssingle-usedisposables) may contribute to contaminating the environment.	2 and 3
	Pads are often promoted without disposal implications, and may end up in rivers, forests; more attention may be needed for menstrual product disposal and waste management.	2 and 3
**#16 Peace, Justice and Strong Institutions**	Marginalized groups may not receive attention/resources/support for MHH (e.g. homeless, transgender and non-binary, refugees).	4
	Gender-based violence (GBV) may inhibit access to MHH needs; may experience vulnerability to GBV near toilets.	4
**#17 Partnerships for the Goals**	Persistence and impact of gender unequal taxation (e.g. menstrual products as luxury goods) may negatively impact those who menstruate.	3
	Could address the intersection women’s empowerment and health.	3
	MHH may be essential to meet education for all, and a reduction of the ongoing gender gap in schooling.	3
	Public-private partnerships may be essential for WASH delivery, menstrual product provision, etc.	3
	Could encourage cooperation on access to menstrual related technologies and innovations including those related to menstrual disposal solutions that could be adapted to local contexts.	3
[24]		

As another example, menstruation is also linked to SDG 3.7 which aims to ensure universal access to sexual and reproductive health services (of which menstruation support is a critical element) and was also promised in the Beijing Platform for Action, and to SDG 5.3 which calls for the elimination of ‘child, early and forced marriage.’ In some contexts, the onset of menarche may trigger a girl’s vulnerability to early marriage [20] given the perception that she is now able to reproduce [13,21].

Further, as a final example, menstruation relates to SDG 4 promoting ‘inclusive and equitable quality education.’ The lack of adequate, safe, clean toilets in schools may impact a girl or female teacher’s abilities to engage effectively in the learning process [22–24].

Despite the relevance of menstruation across a range of global goals, the research, monitoring and evaluation (M&E), and advocacy around MHH have primarily focused on the contribution of unmet MHH needs to outcomes such as school attendance and dropout. Other important linkages, such as impacts on psychosocial wellbeing [15,25], sexual and reproductive health [26–28], and other educational outcomes have not received equivalent attention [29–32]. Many sectors, such as Gender and Health, may not as of yet considered the role of MHH in contributing to the achievement of their own priority outcomes.

## Linking sector priority outcomes with MHH and SDGs

**Figure 3.**
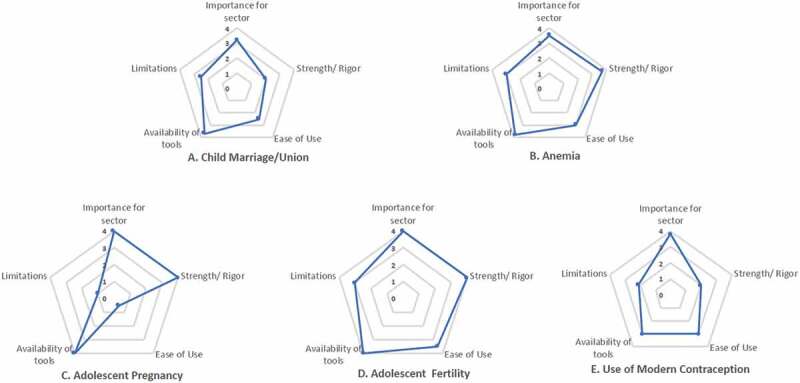
Spider diagram analyses of Sexual and Reproductive Health (SRH) and MHH aligned outcome measures

The spider diagrams (Steps 4 and 5) enabled experts to appraise the top 3–4 priority outcomes per sector for their level of impact, relevance to MHH, and readiness for uptake more broadly. For the exercise (see exemplars in Figure 3), each group was asked to score each indicator in terms of importance for the sector, strength/rigor, ease of use, availability of tools (to measure), and limitations for uptake and scaled use. Insights included, for example, how from a sectoral perspective, the MHH alignment may not always be as evident given the lower position of MHH on the results chain in terms of contributing to the ultimate impact of a program or policy. Case in point, a national child marriage policy might not consider age of menarche as a relevant factor (see Table 2; which only includes the top 3–4 selected priority outcomes (with related indicators) per sector, although additional outcomes were identified as aligned with MHH.).

**Table 2.** Examples of sector priority outcomes’ alignment with Menstrual Health and Hygiene (MHH)

Sector priority outcomes Linked to SDGs	Alignment with MHH
***Education sector***	
Proportion of schools, and teachers in school who have received high quality gender-sensitive teacher training	Teacher training enables effective and sensitive delivery of MHH guidance and support for menstruating girls.
Proportion of schools that provide life-skills-based HIV and sexuality education (SDG 4.7.2)	Curricula that include comprehensive sexuality education (CSE). MHH content and teaching methods that raise awareness for girls, boys and teachers, help challenge discriminatory gender norms and destigmatize menstruation.
Attendance, completion rates (primary, lower and upper secondary education) (SDG 4.1.2, 4.1.4 and 4.1.5), transition rates (from primary to lower secondary education), disaggregated by sex	May restrict ability to participate and engage in school during menstruation, and related absenteeism. Potential implications of menarche for child marriage and/or early pregnancy.
Learning achievement (proportion of children and young people in grade 2/3, end of primary and end of lower secondary achieving at least a minimal level of proficiency in reading and mathematics disaggregated by sex (SDG 4.1.1)	Inadequate, educational, social and physical support for menstruating girls may impact confidence, concentration and engagement in classroom; performance on academic activities.
***Health sector (psychosocial)***	
Proportion of girls suffering psychological distress and anxiety associated with MHH (SDG 3.8.1)	Shame, stigma/teasing, and other menstruation-related stressors (e.g. pain, lack of materials) may lead to psychological distress.
Proportion of girls diagnosed with mental health issues related to MHH (SDG 3.8.1)	Inability to participate fully in valued/important social activities; the negative impact that not participating in educational/economic activities can have on mental health.
Proportion of girls reporting stigma associated with their MHH	Internationalized menstrual stigma around menarche and menstruation.
***Health sector (sexual and reproductive health)***	
Adolescent birth rate (10–14 year olds and 15–19 year olds) (SDG 3.7)	Limited menstruation-related knowledge (and related social norms) may constrain ability to make decisions about sex and pregnancy prevention; sexual risk-taking behaviors; need for transactional sex due to economic vulnerability and need for menstrual supplies; lack of sexual decision-making authority within relationships due to gender power disparities.
Proportion of girls in a given setting with anemia (moderate or severe)	Lack of reproductive health care services, ongoing anemia may contribute to poor health outcomes.
Contraception (modern contraceptive prevalence, disaggregated by age) (SDG 3.7.1)	Knowledge, bodily autonomy/empowerment, management of CIMBCs*.
Proportion of girls <19 years forced into child marriage (SDG 5.3.1)	Menarche may indicate readiness for marriage in societies where women are first seen as spouses, mothers and caregivers
***WASH sector***	
Proportion of schools with female-friendly WASH facilities (SDG 6.2 and SDG 4.a.1)	Enables MHH while in classroom and at school for female students, teachers and administration
Proportion of women and girls able to manage MHH specific needs at home (SDG 6.2)	Enables MHH while at home
Proportion of health care facilities with acceptable female-friendly WASH facilities (SDG 6.2 and SDG 3.8.1)	Enables MHH while in health care facilities and supports targets for universal health coverage and quality of care, etc.
Proportion of women and girls in refugee camps able to manage MHH specific needs (SDG6.2)	Enables MHH in refugee camps and other vulnerable situations
***Gender sector***	
Gender norms and discriminatory practices (SDG 5.1)	Shape norms around menstruation – impacts menstrual health, while menstrual specific norms reinforce some gender norms/expectations; such as policies and tax regulations that support those who menstruate; or practices that limit those who menstruate from engaging in religious life.
Proportion of girls forced into child labor, including domestic work (SDG 5.1.1)	Gender enabling environments – including for MHH – needed beyond schools.
Proportion of girls <19 forced into child marriage (SDG 5.3.1)	Menarche may indicate readiness for marriage, negatively impacting efforts to empower girls as they transition into adulthood.
Gender budgeting or resource allocation for gender equity (SDG 5.c.1)	Enables the financing of MHH related interventions.
Proportion of girls experiencing gender-based violence (SDG 5.2.2)	Menarche may increase vulnerability of girls to sexual violence, sexual coercion, adolescent births and early marriage.
[24]	

*CIMBC = Contraceptive-Induced Menstrual Bleeding Changes, may include bleeding patterns which are predictable but deviate from a typical menstrual pattern, such as amenorrhea induced by some long-acting contraceptives [34].

In addition, prioritized outcome measures may require higher levels of financial or human capacity resources, limiting their usefulness for wider uptake and use at national and local levels. This is illustrated by the Education sector indicators, such that the *availability and quality of ggender-sensitiveteacher training* has a high perceived impact for education, with easy measurement of training presence or absence, but difficulties and high costs to conducting and assessing training quality. This differs from the outcome measure of *transition (grade) rates* for education, which serves as an output leading to an outcome and is captured easily, albeit not always rigorously in countries with poor data systems. Both are considered high impact in the Education sector, are related to MHH, and need gender supportive school environments, highlighting the potential role of menarche and MHH in hindering grade progression (see Table 2).

Additional insights from the spider diagram exercise, which analyzed the 16 different outcome indicators identified across the sectoral areas (see Table 2), included, for example, that while adolescent pregnancy is a high-impact measure, with those newly menstruating more vulnerable to becoming pregnant, it can be harder to measure and thus perhaps is not viable for national monitoring. In general, countries are more likely to measure adolescent birth rate than pregnancy given the ethical and logistical challenges of sampling and assessing who is pregnant. Identifying pregnancy among schoolgirls is hampered by the custom across different settings to expel girls from school [33]; girls thus hesitate to declare pregnancy until it is clearly observable, and conducting pregnancy tests risks confidentiality disclosure. Another insight based on the spider diagrams, as portrayed above (see Figure 3), relates to anemia, a critical measure for girls’ and women’s health, with adequate tools available. However, the difficulty in routine collection of blood to measure hemoglobin reduces the utility of this measure.

## Strengths and limitations

Key strengths of this undertaking included the extensive consultation process undertaken with stakeholders, including experts both experienced in menstrual health and more broadly among international expertise involved with decision-making for other sectors. Further, a systematic approach was used to examine key measures across sectors and document synergies. An important limitation was the potential bias amongst the experts included in the monitoring measures meeting group and global advisory group; although many were not engaged in MHH activities, they were selected based on their measurement expertise, role within global monitoring, and willingness to explore the potential alignments between MHH and priority sectors.

## Conclusion

Important alignments exist between MHH and priority outcome measures for sectoral areas of relevance to girls in and out of school in low- and middle-income countries. These are essential to making progress on a broad array of SDGs. However, challenges related to limited sectoral understanding of MHH as relevant to making progress beyond sanitation, and insufficient validated outcome measures at programmatic and national levels, must be addressed. For the MHH community, future efforts should seek to (a) test these hypothesized relationships and the effect of unmet MHH needs to these outcomes; and (b) include these outcomes in trials for MHH interventions to assess impact on these sector-specific priority outcomes. Those who have not previously engaged with menarche and menstruation should (c) consider the contribution of MHH to these outcomes in national level monitoring, with the expectation that mediating or more proximal MHH indicators would likely be needed to claim that improvements in MHH were having the articulated impact on sectoral outcomes. MHH-related indicators should also be considered in problem and program theories related to these outcomes in other research efforts. Lastly, (d) there were a number of SDGs that may be perceived as either contributing (but not recognized) to making progress on MHH, or that could serve as a proxy for actual progress; these are important to consider as well moving forward. Efforts should go beyond low- and middle-income countries to incorporate the globe.

## Supplementary Material

Supplemental Material

## Paper context

There is an overdue need to assess the importance of menstrual health and hygiene across the entirety of the s17 Sustainable Development Goals, and highlight the sectors that would benefit from addressing menstruation when seeking to achieve goal-specific outcomes. To fully achieve the goals by 2030, it is necessary to recognize menstruation as a contributing factor. In this paper, we outline the potential linkages between menstrual health and hygiene and all of the goals.
