# Supplementary material for: How addressing menstrual health and hygiene may enable progress across the Sustainable Development Goals
Source: Glob Health Action. 2021 Jun 30;14(1):1920315. doi: 10.1080/16549716.2021.1920315 (PMC8253211; doi:10.1080/16549716.2021.1920315)
Supplement: Supplemental Material [file ZGHA_A_1920315_SM8296.zip › Supplementary files/Supple.pdf]

Monitoring MHM: Making progress on menstruation

BRIEFING DOCUMENT – MARCH 11-13TH 2019, GENEVA MEETING

Supplemental Materials, Suggested Citation: Sommer, M., Weston, A., Torondel, B., Caruso, B., Haver, J., Mahon, T., Phillips-Howard, P. (2019), Monitoring Menstrual Health and Hygiene Meeting: Briefing document. New York and Geneva: Columbia University and WSSCC.

TABLE OF CONTENTS

(1) Summaries of studies (e.g. trial, quasi-experimental, cross-sectional) 3
(2) Systematic Reviews (summary table) 14
(3) Excel spreadsheet with indicators (**to be sent separately from this document*)

CITATION LIST

Montgomery, P., Ryus, C. R., Dolan, C. S., Dopson, S., & Scott, L. M. (2012). Sanitary Pad Interventions for Girls Education in Ghana: A Pilot Study. *PLoS ONE, 7*(10), 1-7. doi:10.1371/journal.pone.0048274

Oster, E., & Thornton, R. (2009). Menstruation and Education in Nepal. *The National Bureau of Economic Research*. doi:10.3386/w14853

Haque, S. E., Rahman, M., Itsuko, K., Mutahara, M., & Sakisaka, K. (2014). The effect of a school-based educational intervention on menstrual health: An intervention study among adolescent girls in Bangladesh. *BMJ Open, 4*(7). doi:10.1136/bmjopen-2013-004607

Phillips-Howard, P. A., Nyothach, E., Kuile, F. O., Omoto, J., Wang, D., Zeh, C., . . . Laserson, K. F. (2016). Menstrual cups and sanitary pads to reduce school attrition, and sexually transmitted and reproductive tract infections: A cluster randomised controlled feasibility study in rural Western Kenya. *BMJ Open*, *6*(11), 1-11. doi:10.1136/bmjopen-2016-013229

Das, P., Baker, K. K., Dutta, A., Swain, T., Sahoo, S., Das, B. S., ... & Mishra, P. R. (2015). Menstrual hygiene practices, WASH access and the risk of urogenital infection in women from Odisha, India. *PloS one*, *10*(6), e0130777.

Montgomery, P., Hennegan, J., Dolan, C., Wu, M., & Scott, L. (2016). OP47 Menstruation and the cycle of poverty: A cluster quasi-randomised control trial of sanitary pad and puberty education provision in Uganda. *Journal of Epidemiology and Community Health*, *70*(Suppl 1). doi:10.1136/jech-2016-208064.47

Freeman, M. C., Greene, L. E., Dreibelbis, R., Saboori, S., Muga, R., Brumback, B., & Rheingans, R. (2011). Assessing the impact of a school-based water treatment, hygiene and sanitation programme on pupil absence in Nyanza Province, Kenya: A cluster-randomized trial. *Tropical Medicine & International Health*. doi:10.1111/j.1365-3156.2011.02927.x

Muthengi, E., & Austrian, K. (2018). Cluster randomized evaluation of the Nia Project: Study protocol. *Reproductive Health, 15*(1). doi:10.1186/s12978-018-0586-4

Garazi Zulaika, Daniel Kwaro, Elizabeth Nyothach, Duolao Wang, Emily Zielinski-Gutierrez Linda Mason, Alie Eleveld, Tao Chen, Emily Kerubo, Annemieke van Eijk, Cheryl Pace, David Obor, Jane Juma, Boaz Oyaro, Louis Niessen, Godfrey Bigogo, Isaac Ngere, Carl Henry, Maxwell Majiwa, Clayton O. Onyango, Feiko O. ter Kuile, Penelope A. Phillips-Howard. (2019). Menstrual cups and cash transfer to reduce sexual and reproductive harm and school dropout in adolescent schoolgirls: protocol of a cluster-randomised controlled trial in western Kenya *(In Progress)*

Emily Wilson, Calum Smith, Steven Julious, Laura Flight, Josie Reynolds. (2019). Does Menstrual Hygiene Matter: Investigating the Impact of a Menstrual Hygiene Program (Reusable Sanitary Pad and Menstrual Health Education) on Rural Ugandan Girls' School Absenteeism *(In Progress)*

Alam, M., Luby, S. P., Halder, A. K., Islam, K., Opel, A., Shoab, A. K., . . . Unicomb, L. (2017). Menstrual hygiene management among Bangladeshi adolescent schoolgirls and risk factors affecting school absence: Results from a cross-sectional survey. *BMJ Open, 7*(7). doi:10.1136/bmjopen-2016-015508

Hennegan, J., Dolan, C., Wu, M., Scott, L., & Montgomery, P. (2016). Measuring the prevalence and impact of poor menstrual hygiene management: A quantitative survey of schoolgirls in rural Uganda. *BMJ Open, 6*(12). doi:10.1136/bmjopen-2016-012596

Preswich, G. (n.d.). An exploratory study into Menstrual Hygiene Management amongstrural, primary schoolgirls in Uganda: What implications does menstrual related absenteeism have for future interventions? 1-37. Retrieved February 28, 2019, from http://www.irise.org.uk/uploads/4/1/2/1/41215619/prestwich_georgina_dissertation.pdf

Miiro, G., Rutakumwa, R., Nakiyingi-Miiro, J., Nakuya, K., Musoke, S., Namakula, J., . . . Weiss, H. A. (2018). Menstrual health and school absenteeism among adolescent girls in Uganda (MENISCUS): A feasibility study. *BMC Womens Health, 18*(1). doi:10.1186/s12905-017-0502-z

SUMMARIES OF STUDIES

Sanitary Pad Interventions for Girls' Education in Ghana: A Pilot Study
Paul Montgomery, Caitlin R. Ryus, Catherine S. Dolan, Sue Dopson, Linda M. Scott

DESIGN:
- Three-arm non-randomized pilot study

STUDY POPULATION
- 120 participating schoolgirls aged 12-18 years in primary and junior secondary schools in 1 rural and 3 periurban schools in Ghana
- Eligibility schools – schools selected in collaboration with NGO partner based on the criteria of having sufficient school population, a gender disparity in school enrollment, community acceptance, and enthusiastic support for the project
- Eligibility participants – all girls 12-18y identified by teachers were enrolled

INTERVENTION:
- (i) pads with puberty education (2 schools- rural and periurban), (ii) puberty education alone (1 periurban school), and (iii) control (1 periurban school).

METHODS
- Follow-up over 5 months, examining school registers for attendance

OUTCOME MEASURES:
- Primary: school attendance

RESULTS:
- After 3 months, pads with education significantly improved attendance among participants, (lambda 0.824, F=3.760, p,.001). After 5 months, attendance among girls provided puberty education alone (M=91.26, SD=7.82) was similar to girls provided pads with puberty education (Rural M=89.74, SD=9.34; Periurban M=90.54, SD=17.37), all of which were higher than control (M=84.48, SD=12.39). The total improvement through pads with education intervention after 5 months was a 9% higher attendance compared with periurban control.

LIMITATIONS:
- Limited generalizability, very small sample size, short follow-up time
- Menstruation not an eligibility requirement.
- Control was only peri-urban
- No differentiation of absence, migrated, dropped-out

Menstruation, Sanitary Products and School Attendance: Evidence from a Randomized Evaluation
Emily Oster, Rebecca Thornton

DESIGN
- 2-arm quasi-randomized controlled trial

STUDY POPULATION
- 198 participating girls averaging age 14 in 7th and 8th grade among 2 urban and 2 peri-urban schools in Nepal
- Eligibility of schools – no information on selection
- Eligibility of participants – girls in 7th and 8th grade from selected schools and their mothers who attended meeting

INTERVENTION
- Random assignment of mother-daughter pairs to treatment (menstrual cup) and control

METHODS
- In-school nurse visit once per month, over 15 months
- Baseline and follow-up surveys
- Monthly diaries self-completed by participating girls

OUTCOME MEASURES

- Primary: School attendance
- Secondary: test scores, gynecological health, self-esteem

RESULTS

- Menstrual cup use has no significant effect on school attendance.
- The primary benefit of menstrual sanitary products is convenience of MHM and increased mobility
- Girls on average miss 0.40 days due to menstruation over a 180 day school year
- Girls who were menstruating were 3 percentage points less likely to be in school on days they were menstruating.
- Providing menstrual cups resulted in a 0.021 percentage point increase in the likelihood of attendance on school days when girls have their period, equivalent to a gain of 0.5 days of school per year.

LIMITATIONS

- The only limitations addressed were related to measuring self-esteem and empowerment
- No evaluation of whether participants given cups actually used them
- No differentiation of absence, migrated, dropped-out
- Small sample size

The effect of a school-based educational intervention on menstrual health: an intervention study among adolescent girls in Bangladesh
Syed Emdadul Haque, Mosiur Rahman, Kawashima Itsuko, Mahmuda Mutahara, Kayako Sakisaka

DESIGN

- Intervention study comparing change compared with baseline

STUDY POPULATION

- 416 adolescent female students aged 11–16 years, in grade 6–8, in 3 randomly selected schools Bangladesh
- Eligibility of schools – simple random sampling of all schools in area, that were well established older and conveniently located
- Eligibility of participants - (1) they were in grades 6–8, (2) were not critically ill, and (3) had achieved menarche

INTERVENTION

- school-based menstrual education programme 6 months of educational intervention by trained (by an obstetrician and gynaecologist) research assistants (RAs) on menstrual hygiene among school girls.

METHODS

- In-school nurse visit once per month, over 15 months
- Baseline and follow-up surveys
- Monthly diaries self-completed by participating girls

OUTCOME MEASURES

- Change in knowledge and beliefs on menstruation

RESULTS

- significant improvement ($p<0.001$) in 'high knowledge and beliefs' scores compared to baseline (51% vs 82.4%).
- Significant improvement in overall good menstrual practices (28.8% vs 88.9%), including improvements in using sanitary pads (22.4% change after the intervention), frequency of changing pads/cloths per day (68.8%), drying the used absorbent (77.6%), methods of disposing of the used absorbent (25.5%), and cleaning of genitalia (19.2%).
- reported significant improvements in the regularity of their menstrual cycle (94.5% vs 99.5%) and fewer complications during menstruation (78.6% vs 59.6%).

LIMITATIONS

- Baseline v follow-up, no control population; some changes may be due to aging of participants
- Short follow-up time

Menstrual cups and sanitary pads to reduce school attrition, and sexually transmitted and reproductive tract infections: a cluster randomized controlled feasibility study in rural Western Kenya.
Penelope A Phillips-Howard, Elizabeth Nyothach, Feiko O ter Kuile, Jackton Omoto, Duolao Wang, Clement Zeh, Clayton Onyango, Linda Mason, Kelly T Alexander, Frank O Odhiambo, Alie Eleveld, Aisha Mohammed, Anna M van Eijk, Rhiannon Tudor Edwards, John Vulule, Brian Faragher, Kayla F Laserson

DESIGN
- 3-arm open-label, cluster randomized controlled feasibility study in Kenya

STUDY POPULATION
- 751 participating girls aged 14-16 years, in 30 primary schools, (10 schools per arm).
- Eligibility of schools – co-ed or girls day schools; primary schools in the study site (Gem sub-County); schools only selected if latrine: pupil ratio was <70:1, privacy wall in place, water present for handwashing at baseline
- Eligibility of participants - reached menarche, day school student at enrolment, no severe disability precluding participation

INTERVENTION
- (i) menstrual cup, (ii) monthly sanitary pads, and (iii) control. All participants received puberty education preintervention, and hand wash soap termly throughout. Schools received hand wash soap.

METHODS
- Baseline observations through self-report survey
- Nurse follow-up in school of participants termly (nurse and girls questionnaires) for evaluating use, acceptability, safety of interventions
- Pre-intervention v post-intervention *staph aureus* prevalence survey
- Physical observation of use of cups
- Girls self-completed calendars
- Schools register for attendance
- Unannounced longitudinal WASH surveys in school used to evaluate varying WASH, student headcounts
- Endline observations through self-report survey, STI, RTI lab study
- Baseline, midline, endline qualitative studies
- Home checks to differentiate dropout, migration, from absence
- Intent to treat analysis used

OUTCOME MEASURES
- Primary: school attrition (drop-out, absence)
- Secondary: sexually transmitted infection (STI), reproductive tract infection (RTI); safety: toxic shock syndrome, vaginal S. aureus; e coli on menstrual cups.

RESULTS
- Cups or pads did not significantly reduce school dropout rate (control=8.0%, cups=11.2%, pads=10.2%).
- Self-reported absence was rarely reported and not assessable (separate paper evaluating absence reports 5% difference between pads and controls).
- Prevalence of STIs at end-of-study survey was significantly lower among intervention (cups 4.2%; pads 4.5%) v controls 7.7%;
- RTI prevalence was 21.5% cups, 28.5% pads and 26.9% control, 71% of which were bacterial vaginosis.
- Bacterial vaginosis prevalence was lower in the cups (12.9%) compared with pads (20.3%), and control (19.2%)
- Greatest differences were seen after 9 months; illustrating familiarization phase needed for cup users.

LIMITATIONS
- Trial not registered before participant follow-up started

- Frequent nurse follow-ups may have positively influenced participants' attitudes to attending school, affecting school outcomes
- Eligibility of post-menarche in primary school required longer enrollment reducing total time under intervention among later enrolled participants
- Dropout and migration differentiated from absence reducing 'absence' numbers recorded on registers (endpoint analysis only conducted on non-migrations, reducing 751 to 644)
- Familiarity with cup use took ~6months reducing potential effect size (analyses following 9m-12m use had reduced sample size compromising statistical precision)

Menstrual Hygiene Practices, WASH Access and the Risk of Urogenital Infection in Women from Odisha, India
Padma Das, Kelly K. Baker, Ambarish Dutta, Tapoja Swain, Sunita Sahoo, Bhabani Sankar Das, Bijay Panda, Arati Nayak, Mary Bara, Bibiana Bilung, Pravas Ranjan Mishra, Pinaki Panigrahi, Sandy Cairncross, and Belen Torondel

DESIGN

- Hospital-based case-control study to determine the association of MHM practices with urogenital infections, cases and controls recruited using a syndromic approach

STUDY POPULATION

- 486 women in Idosha, India, 228 symptomatic cases and 258 asymptomatic controls.
- Eligibility of participants: women who sought care from the gynecology department for abnormal vaginal symptoms, and women attending the Family welfare department for anti-fertility measures (IUDs)

INTERVENTION

- 4 case groups: participants experiencing one or more symptoms of urogenital disease, participants whose laboratory tests were positive for BV regardless of symptoms, participants whose laboratory tests were positive for UTI regardless of symptoms, and participants with a positive test result for either UTI or BV

METHODS

- Collection of vaginal and urinary specimens
- Female interviewers used a standardized questionnaire to collect socio-economic, MHM practice and hygiene information

OUTCOME MEASURES

- Reusable absorbent pad use, disposable pad use, increased wealth and space for personal hygiene, lower education, interventions that ensure women have access to private facilities with water for MHM and that educate women about safer, low-cost MHM materials that could reduce urogenital disease among women.

RESULTS

- Women who used reusable absorbent pads were more likely to have symptoms of urogenital infection (AdjOR=2.3, 95%CI1.5-3.4) or to be diagnosed with at least one urogenital infection (BV or UTI) (AdjOR=2.8, 95%CI1.7-4.5), than women using disposable pads
- Increased wealth and space for personal hygiene in the household were protective for BV (AdjOR=0.5, 95%CI0.3-0.9 and AdjOR=0.6, 95%CI0.3-0.9 respectively)
- Lower education of the participants was the only factor associated with UTI after adjusting for all the confounders (AdjOR=3.1, 95%CI1.2-7.9)

LIMITATIONS

- Observational study- cannot determine causality
- Residual confounding may have remained when adjusting for self-reported risk factors
- Did not adjust for other possible related factors, such as sexual practices or other infections (STIs and HIV)
- Study was underpowered, 84% of the control group had access to sanitation and 77% of the control group had access to an improved water source.

Menstruation and the Cycle of Poverty: A Cluster Quasi-Randomized Control Trial of Sanitary Pad and Puberty Education Provision in Uganda
Paul Montgomery, Julie Hennegan, Catherine Dolan, Maryalice Wu, Laurel Steinfield, Linda Scott

DESIGN:
- 4-arm cluster quasi-randomized controlled trial

STUDY POPULATION
- 1124 girls in 8 schools aged 10 years and older in Uganda
- Eligibility of schools – 8 schools selected by NGO partner based on reasonable distance, and comparable on varying criteria (distance from services, education quality, toilet facilities, size), based on NGO knowledge
- Eligibility of participants – written parent/girls consent, primary grades 3-5 (aged 10y and above)

INTERVENTION:
- Two schools each received (i) puberty education alone, (ii) reusable pads, (iii) reusable pads with puberty education, (iv) control

METHODS
- Follow-up over 5 months, examining school registers for attendance

OUTCOME MEASURES:
- Primary: school attendance
- Wellbeing during menstruation;
- Psychosocial (strengths and difficulties questionnaire

RESULTS:
- School attendance fell in all groups.
- 55.2% of girls reported that their menstruation caused them to miss school. This did not differ significantly according to condition
- Self-reported missed days during period was 0.93 (0.25) education only; 1.59 (0.43) in pad only, 1.60 (0.43) in pad and education, compared with 2.31(1.82) in control.
- Per protocol analysis used to differentiate a difference between control and pad schools illustrated a 17% difference; intent to treat using imputation ascertained 5% to 25% difference in attendance in best-case and worst case scenarios
- No impact on girls' shame or insecurity during menstruation.
- Girls in the control group were more likely to migrate/dropout compared with intervention condition

LIMITATIONS
- Ages 10-13y enrolled, menstruation not an eligibility criteria
- Trial not registered until after follow-up data
- Intervention fidelity issues around receipt of pads
- 25% of girls completed baseline survey
- Recall bias from self-reported data
- Poor participant retention (57.8% of enrolled)
- No differentiation of absence, migration, drop-out measures
- PP analyses, ITT no effect shown
- Absence change was greatest in girls pre-menarche at follow-up; no difference in pre- and post in girls post-menarche

Assessing the impact of a school-based water treatment, hygiene and sanitation program on pupil absence in Nyanza Province, Kenya: a cluster-randomized trial.
Matthew C Freeman, Leslie E. Greene, Robert Dreibelbis, Shadi Saboori, Richard O. Muga, Babette A. Brumback, Richard D. Rheingans

DESIGN:
- 3-arm cluster randomized controlled trial

STUDY POPULATION
- 138 primary schools, and the school children present
- Eligibility of schools - schools that exceeded the GoK standard for pupil-to-latrine ratio (25:1 for girls and 30:1 for boys) and had a water source within 1 km during the dry season Eligibility of participants – all in selected grades

INTERVENTION
- (i) water treatment and hygiene promotion (WT & HP), (ii) additional sanitation improvement, and (iii) control Schools in the HP & WT intervention arm received a 3-day training of teachers on HP, behavior change and WT methods, regular follow-up visits, handwashing and drinking water containers and a one-time, 1-year supply of chlorine-based point-of-use water disinfectant. Schools in the second intervention arm received the above, in addition to provision of latrines (maximum of seven latrines).

METHODS
- Follow up over 2 years
- Baseline and repeat cross-sectional surveys at follow-up
- Baseline and endline registers of children attending trial schools

OUTCOME MEASURES
- Primary: Knowledge and practice of WASH, school WASH characteristics, pupil reported school absence
- Secondary: improvements in WASH facility access, enrolment and test scores

RESULTS
- Interventions to improve water quality, hygiene behaviors and sanitation in schools reduced absence for girls in two geographical strata (58% reduction in the odds of absence for girls (OR 0.42, CI 0.21–0.85)
- Sanitation improvement in combination with WT and HP resulted in a comparable drop in absence (OR 0.47, 0.21–1.05).
- Boys absence rates did not differ

LIMITATIONS
- Incomplete intervention compliance highlights the challenges of achieving consistent results across all settings.
- Recall bias from self-reported data
- Baseline and endline survey on absence at different times of the year

Cluster Randomized Evaluation of the Nia Project: Study Protocol (*In Progress*)
Muthengi E, Austrian K.

DESIGN
- 4-arm cluster-randomized controlled evaluation trial

STUDY POPULATION
- 3289 girls in class 7 in 140 primary schools in Kenya, aged 10 years and older
- Eligibility of schools – all schools with 25 or more girls in Class 7 were eligible for the study; boarding schools eliminated after review
- Eligibility of participants - all girls in schools with 25 girls in Class 7 were included; if more girls, 25 were randomly selected

INTERVENTION

- 1. control, 2. disposable sanitary pads, 3. reproductive health education, 4. disposable sanitary pad and reproductive health education

METHODS

baseline survey, qualitative data collection, school attendance tracking, and an endline survey at the completion of intervention.

OUTCOME MEASURES

- Short term measures: Education- school attendance, school engagement,
- Short term measures - SRHR knowledge and attitudes, menstrual health knowledge and attitudes, gender norms, self-efficacy
- Long term measures: Education -school retention and school performance
- Long term measures: Experience of unwanted sex, timing of first sex, timing of pregnancy

RESULTS

- Still under follow-up

LIMITATIONS

- Late trial registration
- Study powered to examine short term measures
- Menstruation not an eligibility criteria

Menstrual cups and cash transfer to reduce sexual and reproductive harm and school dropout in adolescent schoolgirls: protocol of a cluster-randomised controlled trial in western Kenya (*In Progress*)
Garazi Zulaika, Daniel Kwaro, Elizabeth Nyothach, Duolao Wang, Emily Zielinski-Gutierrez Linda Mason, Alie Eleveld, Tao Chen, Emily Kerubo, Annemieke van Eijk, Cheryl Pace, David Obor, Jane Juma, Boaz Oyaro, Louis Niessen, Godfrey Bigogo, Isaac Ngere, Carl Henry, Maxwell Majiwa, Clayton O. Onyango, Feiko O. ter Kuile, Penelope A. Phillips-Howard

DESIGN

- 4-arm open label cluster randomized controlled trial

STUDY POPULATION

- 3984 school girls in 96 secondary schools in rural Kenya (24 school clusters per arm);
- Eligibility of schools – co-ed or girls day schools; secondary schools in the study site (Siaya County)
- Eligibility of participants - reached menarche, day school student at enrolment, no severe disability precluding participation,

INTERVENTION

- (i) menstrual cup and training on safe effective use, (ii) cash transfer and literacy training on safe effective use, (iii) menstrual cup and cash transfer, with training on safe effective use of both interventions, (iv) control. All girls receive puberty and hygiene education. Girls in cup and control arms receive hand wash soap. Schools receive handwash soap.

METHODS

- Baseline quantitative survey, HIV, HSV-2 screening
- Baseline qualitative studies (FGD boys, girls, parents, teachers)
- School registers data, termly evaluation
- Annual WASH and survey of school / community programmes to document possible contamination
- Home visits to differentiate dropout, migration, absence
- Cup contamination study half-yearly (longitudinal)
- Rolling surveys on sub-sample on acceptability and use of interventions
- Pharmacovigilance
- Mid-line and endline quantitative self-reported survey, HIV and HSV2 screening
- Mid-line and endline FGD boys, girls, parents, teachers
- Endline F4 exam attendance, results

OUTCOME MEASURES

- Primary: Composite of incident HIV, HSV-2, and/or school dropout
- Secondary: Disaggregate of composite (incident HIV, HSV-2, school dropout); sexual and reproductive health indices (sexually active, age first sex, number sexual partners, current and first age-concordancy of partner, current, 6m, 12m sexual risk behaviors, transactional sex), cash behaviors (work, savings, use of cash), school indices (grade repetition, absence, exam outcomes), menstrual behaviors (type of materials used, hygiene practices).

RESULTS

- pending

LIMITATIONS

- Area of 2500 sq km, travel / communication difficulties
- Increase in cluster sample after lower number of girls non-boarding at enrolment, lower than predicated HIV, HSV-2 at baseline
- nable to conduct large-scale activities in 3rd term each year due to school exams

Does Menstrual Hygiene Matter: Investigating the Impact of a Menstrual Hygiene Program (Reusable Sanitary Pad and Menstrual Health Education) on Rural Ugandan Girls' School Absenteeism (*In Progress*)
Emily Wilson, Calum Smith, Steven Julious, Laura Flight, Josie Reynolds

DESIGN

- Step wedge, 2 arm cluster randomised controlled trial

STUDY POPULATION

- 1200 school girls aged 14-18.in 40 schools in rural Uganda
- Eligibility of schools - selected if a predefined distance apart
- Eligibility of participants – girls not previously in an MHM programme, aged 14-18 years in the selected schools

INTERVENTION

- Schools will receive a menstrual hygiene program, including the distribution of reusable sanitary pads and menstrual health education. Intervention group will be followed up for 12 months. Second group will act as control for the first 6 months then receive intervention at 6 months.

OUTCOME MEASURES

- Primary: number of days of school missed per month (absenteeism)
- Secondary: Knowledge of menstrual hygiene, Impact of menstruation on activities of daily living, self-esteem during menstruation

RESULTS

- pending

LIMITATIONS

- pending

Menstrual hygiene management among Bangladeshi adolescent schoolgirls and risk factors affecting school absence: results from a cross-sectional survey
Mahbub-Ul Alam, Stephen P Luby, Amal K Halder, Khairul Islam, Aftab Ope, Abul K Shoab, Probir K Ghosh, Mahbubur Rahman, Therese Mahon, Leanne Unicomb,

DESIGN

- Cross-sectional study

STUDY POPULATION

- 2332 Bangladeshi schoolgirls 11 to 17 years old in 700 schools from 50 urban and 50 rural clusters.
- Eligibility of schools –sampled the seven government and non-government schools, at both primary and secondary level, nearest to the midpoint of each cluster, no eligibility noted

- Eligibility of participants – reached menarche, in grades 2 - 9

METHODS

- Interviewed schoolgirls using computer-based tablet structured questionnaire in cross sectional survey
- Conducted spot checks in each school for menstrual hygiene facilities schools from March to June 2013

OUTCOME MEASURES

- Girls' attitude and knowledge about menstruation, reason for school absence during menstruation, practices related to menstruation
- Schools having soap and water at handwashing location
- School absence, average number of school absence days in the last three menstrual cycles

RESULTS

- 41% (931) of girls reported missing school, ~2.8 missed days per menstrual cycle.
- Students who felt uncomfortable at school during menstruation (99% vs 32%) and who believed menstruation interferes with school performance (64% vs 30%) were more likely to miss school during menstruation.
- School absence during menstruation was less common among girls attending schools with unlocked or gender separated toilet for girls (35% vs 43%).
- School absence was more common among girls who were forbidden from any activities during menstruation.
- Risk factors for school absence included girl's attitude, misconceptions about menstruation, insufficient and inadequate facilities at school, and family restriction.

LIMITATIONS

- Difficulty measuring attendance, sampling bias possible as selected by teachers

Measuring the prevalence and impact of poor menstrual hygiene management: a quantitative survey of schoolgirls in rural Uganda
Julie Hennegan, Catherine Dolan, Maryalice Wu, Linda Scott, Paul Montgomery

DESIGN

- Nested survey within a randomized controlled trial (see Montgomery, above)

STUDY POPULATION

- 205 menstruating schoolgirls (10–19 years) in 8 study sites in Uganda.
- Eligibility of schools – see Montgomery above
- Eligibility of participants – self reported they reached menarche

METHODS

- Secondary analysis of survey data collected as part of the final follow-up from a controlled trial of reusable sanitary pad and puberty education provision
- Cross-sectional survey of girls' MHM practices using reusable pads.

OUTCOME MEASURES

- The prevalence of adequate MHM: estimated using dimensions of absorbent used, frequency of absorbent change, washing and drying procedures, disposal and privacy.
- Self-reported health, education (school attendance and engagement) and psychosocial (shame, insecurity, embarrassment) outcomes.

RESULTS

- 90.5% of girls failed to meet available criteria for adequate MHM
- High rates of poor MHM
- High rates of negative outcomes, girls reported genital irritation, discharge and concerns about odor.
- There were few associations between aspects of MHM and health symptoms.
- Reporting individual aspects of MHM alone underestimates the extent of deprivation.
- Less than 20% of girls stated that menstruation caused them to miss school, although over half reported not standing in class to answer questions and finding it difficult to concentrate when menstruating.

LIMITATIONS

- Self-reported measures are vulnerable to biases
- Cross-sectional nature of the study limits causal inference,
- Analyses limited by the lack of ability to adjust for potential sociodemographic confounds, the small sample size and lack of existing literature on which to base power analyses.

An exploratory study into Menstrual Hygiene Management amongst rural, primary schoolgirls in Uganda: what implications does menstrual related absenteeism have for future interventions?
Georgina Preswich and IRIS group

DESIGN:

- Formative study with cross sectional survey

STUDY POPULATION

- 140 schoolgirls in six rural primary schools in the Rukungiri District of South-West Uganda
- Eligibility of schools – convenience sample
- Eligibility of participants – all girls (questionnaires); teacher selection for FGD

METHODS

- Self-administered questionnaires
- Focus groups with six girls from each school where the girls were asked to rank solutions to girls' menstrual problems.

OUTCOME MEASURES:

- knowledge, product use, disposable sanitary pads, absenteeism and reasons for menstrual related absenteeism.

RESULTS

- two thirds of girls (61.7%) reported normally missing some days of school in a month because of menstruation with the mean number of days missed at 1.64
- The highest reported reason (63.8%) girls missed school during menstruation was the lack of a private place to wash and change at school
- Knowledge on menstruation was poor amongst the schoolgirls with 36.3% of girls believing that menstruation was a disease

LIMITATIONS

- Language barrier, conceptual barriers with the questionnaire, girls hesitant to talk about topic;
- Cross sectional survey

Menstrual health and school absenteeism among adolescent girls in Uganda (MENISCUS): a feasibility study
George Miiro, Rwamahe Rutakumwa, , Jessica Nakiyingi-Miiro, Kevin Nakuya, Saidat Musoke, Juliet Namakula, Suzanna Francis, Belen Torondel, Lorna J. Gibson, David A. Ross and Helen A. Weiss, *2018*

DESIGN

- Observational formative study

STUDY POPULATION

- Girls and boys aged 14–17 years in 4 secondary schools in Uganda
- Eligibility of schools – purposive sampling of one public Universal Secondary Education one public non-USE;
  two private schools (one high socio-economic status (SES), and one low SES).
- Eligibility of participants – in Forms 2 and 3 in the selected schools

INTERVENTIONS

- Nil

METHODS

- Group and in-depth interviews

- Cross-sectional questionnaire
- Self-completed menstrual diary
- Informant interviews with policy makers,
- Observations of school water, sanitation and hygiene facilities.

<u>OUTCOME MEASURES</u>

- School absenteeism, menstrual knowledge, protection used during last period, times changed protection per 24 h during last period, ever had menstrual accident with blood leaking to clothes, reusable/washable protection, disposable protection, reason for choosing disposable pads, factors associated with missing at least one day of school due to menstruation in the past month, adequate MHM at their last period

<u>RESULTS:</u>

- Reported association between school attendance and menstruation (19.7% reported missing at least 1 day of school during their most recent period) - in the diary sub-study in 40 girls, school absence was reported on 28% of period-days, compared with 7% of non-period days.
- 18.7% of girls reported having stained their clothes
- Missing school during their period was associated with physical symptoms (headache, stomach pain, back pain and with changing protection 4 or more times per 24 h period.
- Recommended a menstrual management intervention that address both psychosocial (e.g. self-confidence, attitudes) and physical (e.g. management of pain, use of adequate menstrual hygiene materials, improved water and sanitation facilities) aspects of menstruation is needed.

<u>LIMITATIONS</u>

- Questions to address all components of the standard definition of adequate MHM not included
- School engagement and performance not captured
- Small sample size within schools
- Not randomized; unclear if only among menstruating girls

SYSTEMATIC REVIEWS: SUMMARY TABLE

**Citation**	Sumpter, C., & Torondel, B. (2013). A Systematic Review of the Health and Social Effects of Menstrual Hygiene Management. PLoS ONE, 8(4). doi:10.1371/journal.pone.0062004	Hennegan, J., & Montgomery, P. (2016). Do Menstrual Hygiene Management Interventions Improve Education and Psychosocial Outcomes for Women and Girls in Low and Middle Income Countries? A Systematic Review. Plos One, 11(2). doi:10.1371/journal.pone.0146985	Eijk, A. M., Sivakami, M., Thakkar, M. B., Bauman, A., Laserson, K. F., Coates, S., & Phillips-Howard, P. A. (2016). Menstrual hygiene management among adolescent girls in India: A systematic review and meta-analysis. *BMJ Open*, *6*(3). doi:10.1136/bmjopen-2015-010290
**Primary outcomes**	Health outcomes: RTIs (bacterial vaginosis and vulvo-vaginal candidiasis), other reproductive infections (secondary infertility), urinary tract infections and anemia	School attendance, employment attendance, Psychosocial outcomes: anxiety, confusion, embarrassment, self-confidence, shame, stigma, self-imposed withdrawal from activities	Information on menarche awareness, type of absorbent used, disposal, hygiene, restrictions, school absenteeism was extracted from eligible materials
**Secondary outcomes**	Social outcomes: limiting diet or interactions during menstruation, school absenteeism	Academic achievement and school engagement, employment productivity and engagement, menstrual knowledge & management	N/A
**Methods**	14 articles were identified which looked at health outcomes, primarily reproductive tract infections	6 studies included assessment of education-only interventions, and 3 provided assessment of the provision of different types of sanitary products	138 studies involving 193 subpopulations and 97 070 girls
**Measures/ Indicators**	'Good' MHM practice, 'Poor' MHM practice (there was no consistency of definitions for 'good and 'bad'), self reported or clinically diagnosed health outcome	Menstrual knowledge, menstrual attitude (debilitating, bothersome, etc), feelings at menarche (confusion, scared, uncomfortable), hygiene practices, menstrual practices, school attendance	Premenarch awareness, knowledge and perception of menstruation and knowledge source, types of menstrual absorbents used, disposal of absorbents, hygiene during menstruation, restrictions, school absenteeism
**Results**	-MHM is associated w/ RTI<br>-Educational interventions can improve MHM practices/reduce social restrictions<br>-No quantitative evidence that improvements in management methods reduce school absenteeism. The effect of poor MHM remains unclear.	-Trials of education interventions reported positive impacts on menstrual knowledge and practices.<br>-Insufficient evidence to establish the effectiveness of menstruation management interventions<br>-Further research is needed to establish the role of MHM in education performance, employment and other psychosocial outcomes	-'menstruation poses a huge physical and psychological burden'. Societal beliefs and restrictions add to these negative experiences. Education could dispel some restrictions (foods or activities), beliefs/negative attitudes are deeply entrenched within Hindu and other religions. Innovative cultural and societal approaches will be required
**Validation**	-Methodologies varied greatly and overall quality was low.<br>-Gaps the evidence for randomized intervention studies which combine hardware and software intervention for understanding the effect improving MHM has on girls' attendance at school.	-Lack of appropriate outcome assessment limits the conclusions.<br>-Unclear outcome measures and self-report biases mean results should be interpreted with caution	-High heterogeneity between studies.<br>-Not all studies reached scientific rigor. -Studies were conducted by self-administered questionnaires or interviews by study staff; may have led to a desirability effect.<br>-Poor quality of studies and lack of information on school sanitation

SYSTEMATIC REVIEWS: SUMMARY TABLE

**Citation**	Wilbur, J., Torondel, B., Hameed, S., Mahon, T., & Kuper, H. (2019). Systematic review of menstrual hygiene management requirements, its barriers and strategies for disabled people. Plos One, 14(2). doi:10.1371/journal.pone.0210974	Birdthistle I, Dickson K, Freeman M, Javidi L(2011) What impact does the provision of separate toilets for girls at schools have on their primary and secondary school enrolment, attendance and completion?: A systematic reviewof the evidence. London: EPPI-Centre, Social Science Research Unit, Institute of Education, University of London. ISBN: 978-1-907345-17-3
**Primary outcomes**	Choice/preference of menstrual management material, ability to manage MH; challenges experienced during menstruation and coping strategies; behavior changes & management.	Primary and secondary school enrollment, attendance and completion
**Secondary outcomes**	N/A	Health (Infectious/vector-borne diseases, sexual health, MHM), social/behavioral issues (bullying and harassment in schools, privacy, humiliation and embarrassment) as casual pathways to primary educational outcomes
**Methods**	22 studies gathered across all countries incorporating analyses of menstruating disabled people and/or their carer support	Identified 5,082 studies through databases or handsearching, 406 were screen on full-text, 73 were coded. No studies were identified that assessed the impact of separate-sex toilets on educational or health outcomes. 12 studies assessed the impact of any school WASH programmes on girls educational outcomes.
**Measures/ Indicators**	Menstrual product acceptability for people with physical and intellectual impairments, MHM training and support for people with intellectual impairments and carers, menstrual suppression	Separate-sex toilets for girls, # of toilets, toilet conditions, hygiene education related to toilets, “girl friendly” toilets with menstrual supplies, handwashing stations, water treatment solutions, teacher training, hygiene education, hardware/software inputs together
**Results**	-Societal beliefs and taboos around menstruation and disability means the issue is shrouded in silence, and that it lacks attention and resources. Without rigorous evidence from different contexts, it is difficult to advocate for greater attention and resourcing to meet the MHM requirements of the disabled.	-Due to a lack of identified studies that assess the impact of separate toilets for girls on their educational outcome, there is no evidence either for or against the impact of separate toilets for girls on their educational outcomes.<br>-Sex-separated toilets are likely not sufficient enough if the toilets are not secure, clean, functional, or private.
**Validation**	-No standardized measurement of MHM outcomes, so outcome assessment across the studies was difficult.<br>-Few studies with consistent methods for a meta-analysis.<br>-Limited number of peer review studies conducted	-Limited time and resources prevented studies in a language other than English from being obtained. Important studies may have been missed as a result
