# Supplementary material for: How addressing menstrual health and hygiene may enable progress across the Sustainable Development Goals
Source: Glob Health Action. 2021 Jun 30;14(1):1920315. doi: 10.1080/16549716.2021.1920315 (PMC8253211; doi:10.1080/16549716.2021.1920315)
Supplement: Supplemental Material [file ZGHA_A_1920315_SM8296.zip › Supplementary files/Supplementary.pdf]

Monitoring Menstrual Health and Hygiene

Foundational presentations from Day One of meeting, Geneva 2019

Suggested Citation: Sommer, M., Phillips-Howard, PA, Caruso, BA, Torondel, B., Haver, J., Gruer, C., Mahon, T. (2019) Monitoring menstrual health and hygiene meeting: Foundational presentations. New York and Geneva: Columbia University and WSSCC. *Supplemental Materials.*

Monitoring Menstrual Health and Hygiene

Measuring Progress on Menstruation

Day One: Foundational Presentations

GENEVA, SWITZERLAND

March 11-13, 2019

Huge thanks

- **WSSCC for travel logistics and support for this meeting**
- **Scientific Technical Advisory Group for planning the agenda**

  Bethany Caruso, Caitlin Gruer, Jackie Haver, Therese Mahon, Penelope Phillips-Howard, Belen Torondel
- **MHM Measures Global Advisory Group for inputs**
- **Document preparation, administrative and logistical support**:

  Caitlin Gruer, Aja Weston, Maggie Schmitt, Garazi Zulaika

Monitoring Menstrual Hygiene Management setting the scene

Thérèse Mahon – WaterAid
Bethany Caruso – Emory University

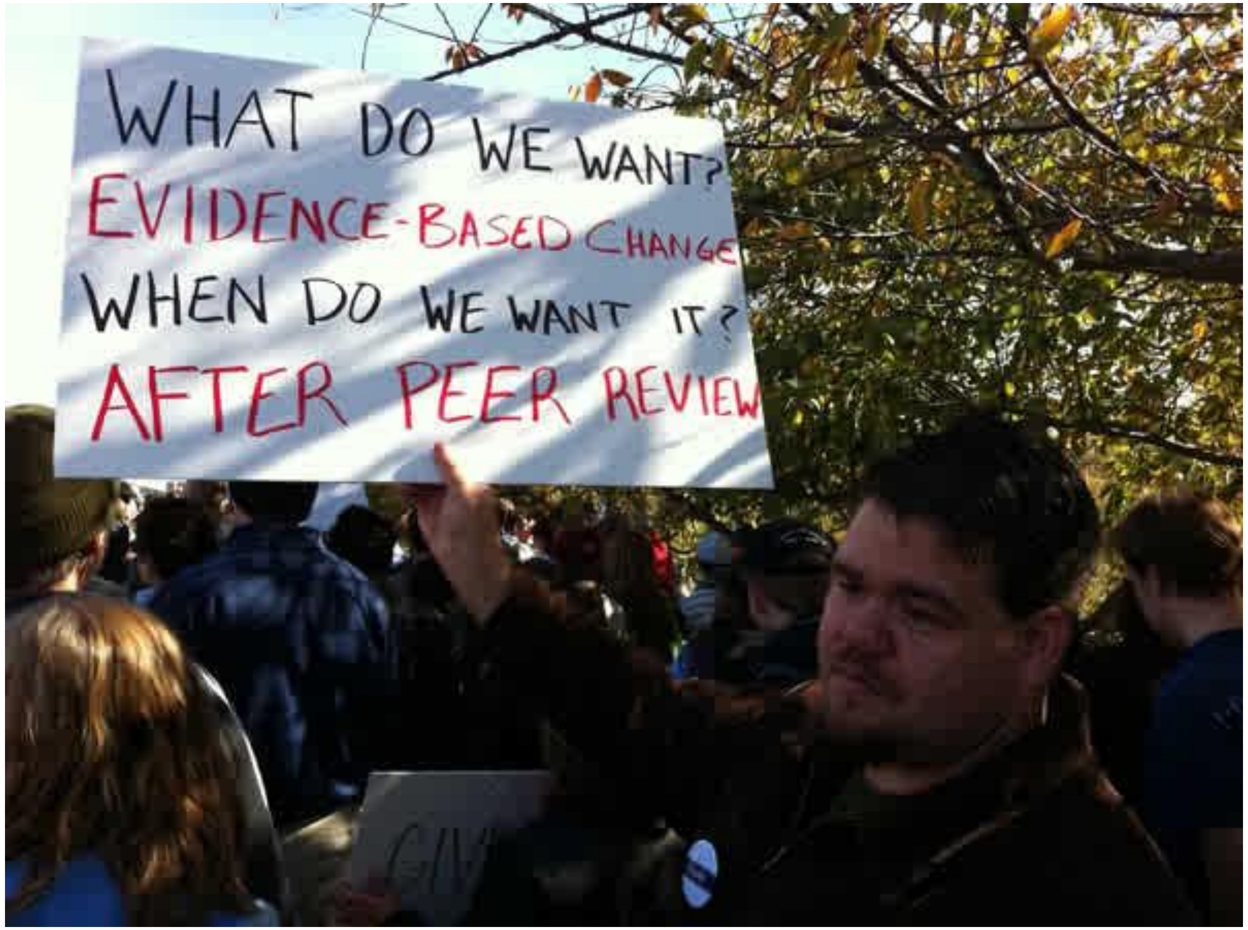

Why Monitor

- What gets monitored gets done
- To track progress (accountability)
- To target resources (efficiency)
- To generate evidence of outcomes and impacts
- To understand what works and what doesn't
- To influence practice, policy and resource allocation

WaterAid

What can be measured gets monitored

A WASH definition of Menstrual Hygiene Management

Women and adolescent girls are using a clean menstrual management material to absorb or collect menstrual blood, that can be changed in privacy as often as necessary for the duration of a menstrual period, using soap and water for washing the body as required, and having access to safe and convenient facilities to dispose of used menstrual management materials.

They understand the basic facts linked to the menstrual cycle and how to manage it with dignity and without discomfort or fear.

Drafted by Joint Monitoring Programme UNICEF/WHO - Hygiene Working Group, 2012

WaterAid

Positioning the WASH definition of MHM

- Advocating for MHM to be included in SDG targets for water and sanitation – as an essential component for gender equality
- Needed a definition that could be monitored at national level and aggregated globally
- Learning from handwashing – focused on the behaviours for menstrual hygiene that could be contextualised
- Could be measured by proxy indicators such as presence of facilities and consumables as well as surveys about practices
- Included knowledge aspect as this was identified as a priority due to taboos and cultural norms

Going beyond MHM definition for monitoring

Limitations:

- Frames menstruation as a hygiene issue that may reinforce taboos around menstruation as a polluting and dirty process – causing stigma and shame
- Does not recognise multiple dimensions of menstruation – sexual and reproductive health, gender, psycho-social health, education
- Is focused at output level – does not consider outcomes / impacts

WaterAid

MHM linkage to SDGs

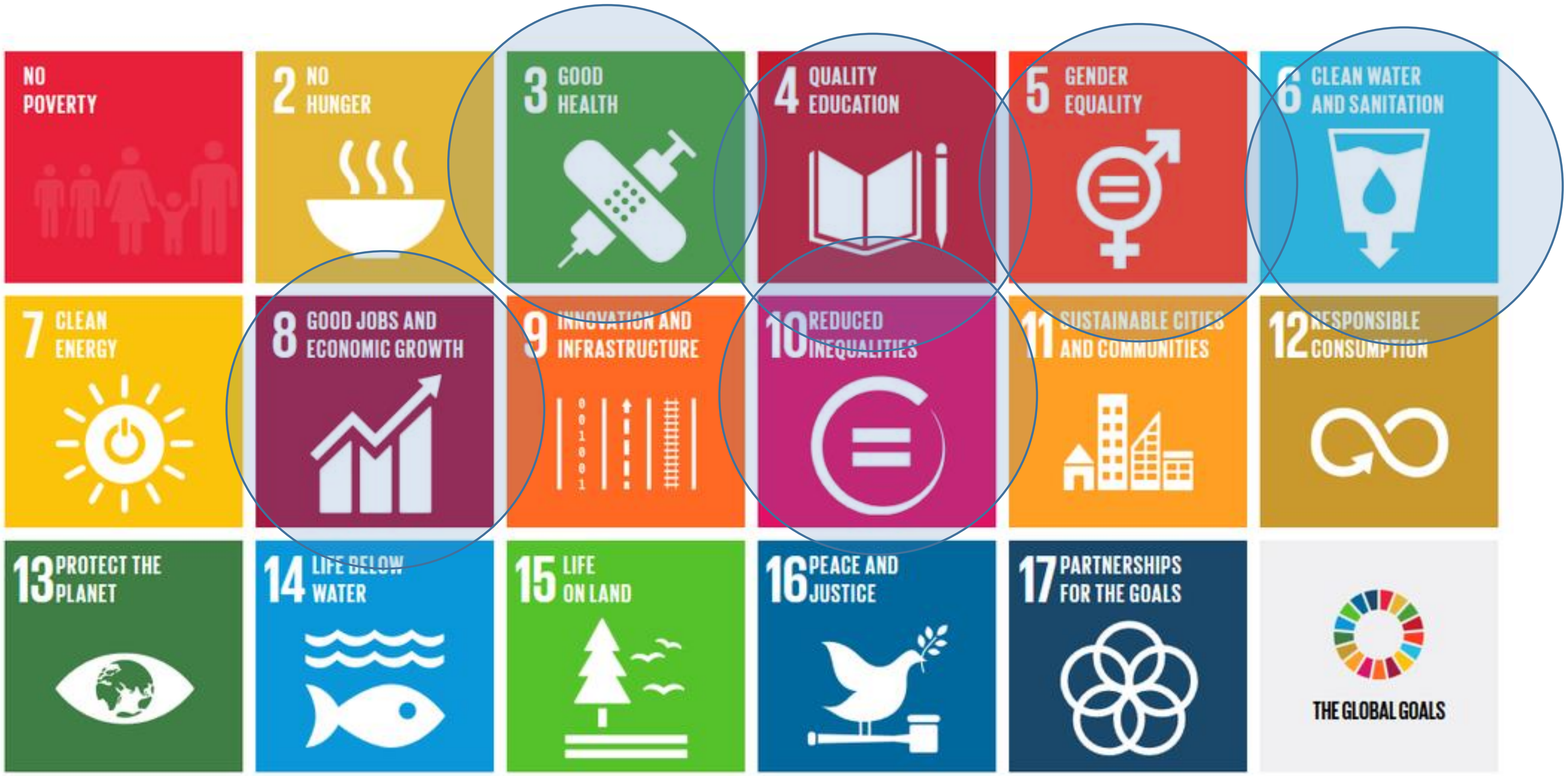

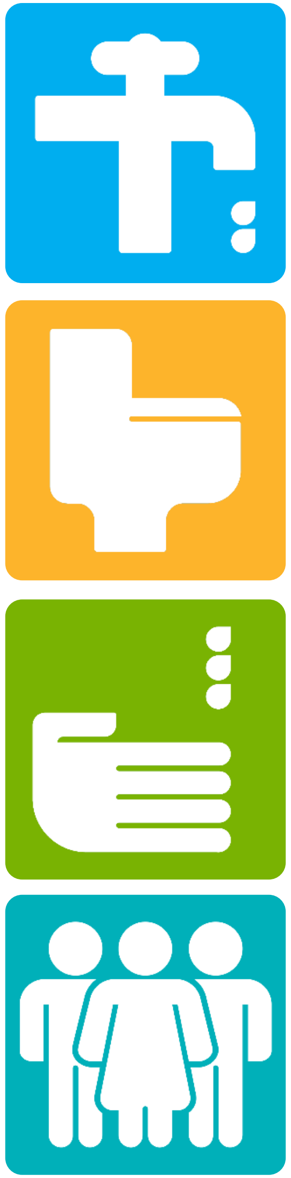

WaterAid

Priority to develop measures for MHM

- Research, programming and rights based advocacy has generated momentum on MHM
- MHM included in SDG 4 (education) and 6 (WASH) – links to more
- Need for definitions that include **SRHR, gender** etc.
- Scaling up MHM and integrating with quality is now a priority
- How can MHM be monitored for tracking progress, quality assurance and demonstrating outcomes – for influencing
- What to monitor? Inputs, outputs, outcomes, impacts

WaterAid

Monitoring MHM:
Measuring Progress on MHM Meeting

On the Same Page:
Terminology & Frameworks

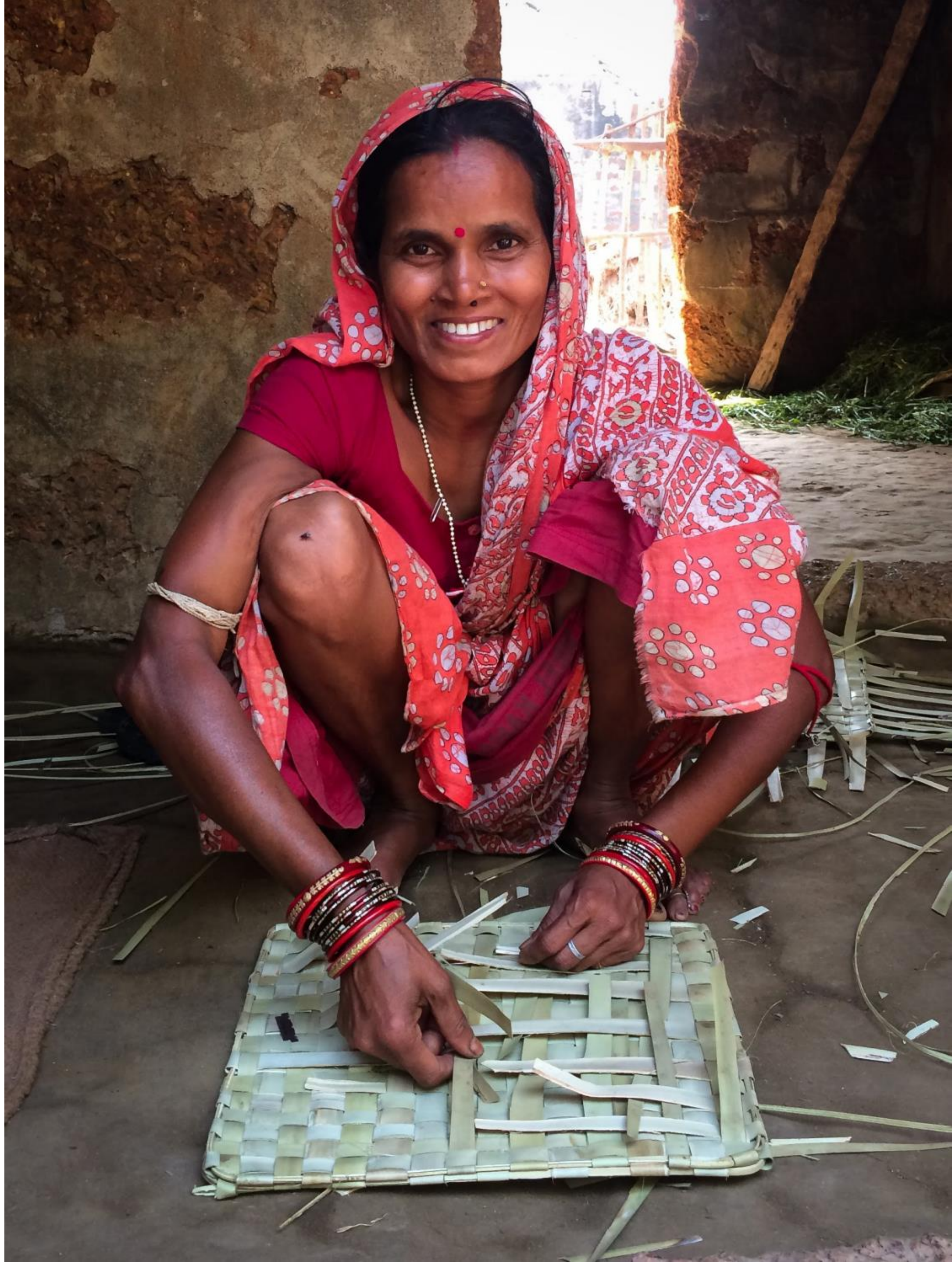

Monitoring MHM:
Measuring Progress on MHM Meeting

Inputs

Specific resources used

Resources

Funds,

Capacity and Expertise,

Staff time

Monitoring MHM:
Measuring Progress on MHM Meeting

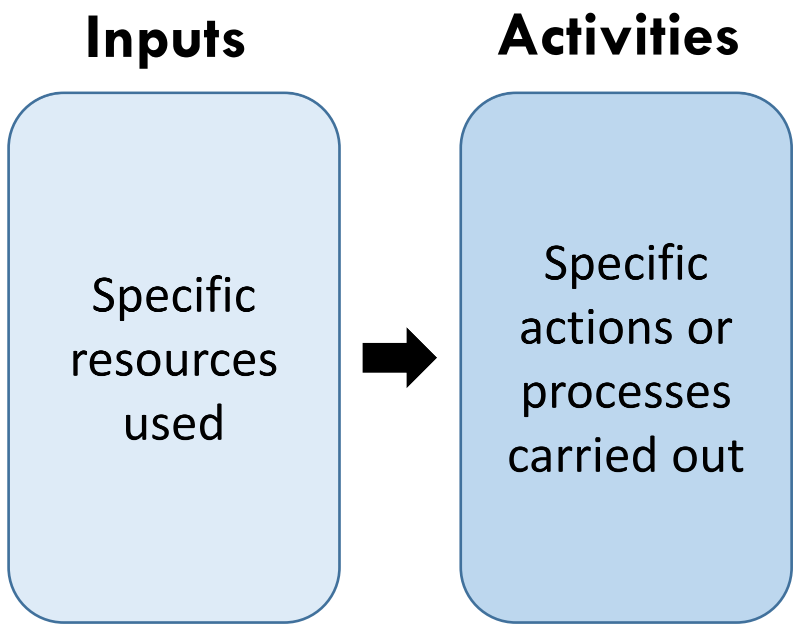

Examples

- *Delivery of MHM trainings and books for girls*
- *Delivery of teacher trainings*
- *Implementation of media campaigns to change norms*
- *Provision of MHM materials*
- *Construction of girl-friendly facilities for MHM*

Monitoring MHM:
Measuring Progress on MHM Meeting

Inputs

Specific resources used

→

Activities

Specific actions or processes carried out

→

Outputs

Direct, tangible results of activities

What project delivers

<u>Examples</u>

- *Increased practical MHM knowledge*
- *Use of facilities to manage menstruation*
- *Use of supplies to manage menstruation*

Monitoring MHM:
Measuring Progress on MHM Meeting

Inputs

Specific resources used

→

Activities

Specific actions or processes carried out

→

Outputs

Direct, tangible results of activities

What project delivers

→

Outcomes

Key Intermediate Change

What Programs hope to see

__Examples__

- *Short-term: Increased self-efficacy managing menstruation at school*
- *Long-term: Improved educational engagement*

Monitoring MHM:
Measuring Progress on MHM Meeting

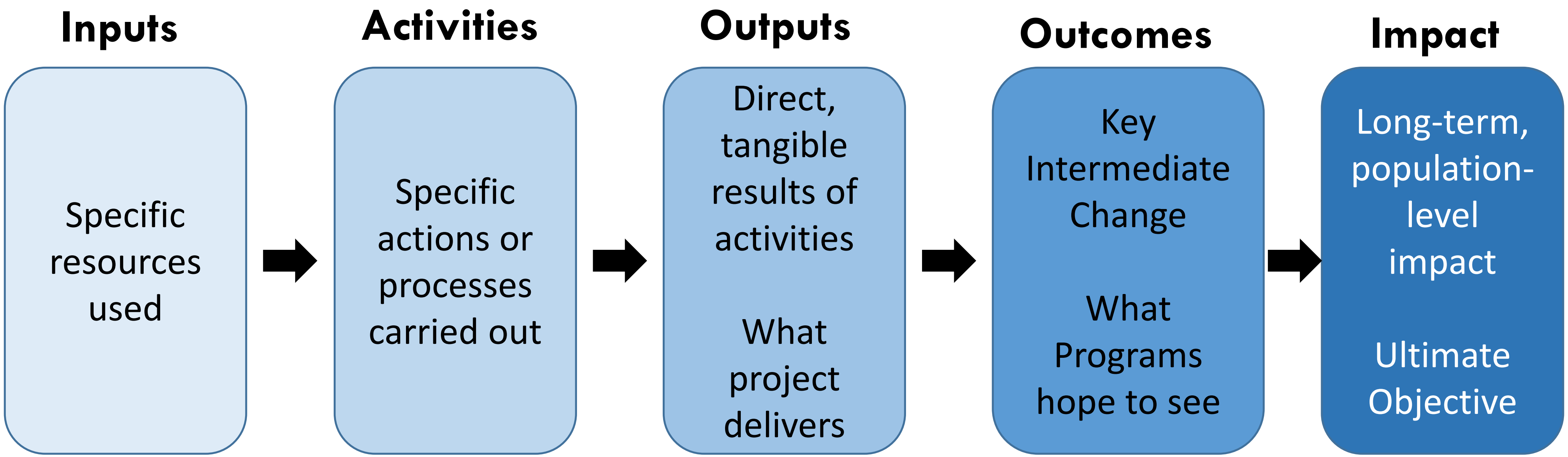

Examples

- *Improved educational equity; increased advancement, reduced dropout*

Monitoring MHM:
Measuring Progress on MHM Meeting

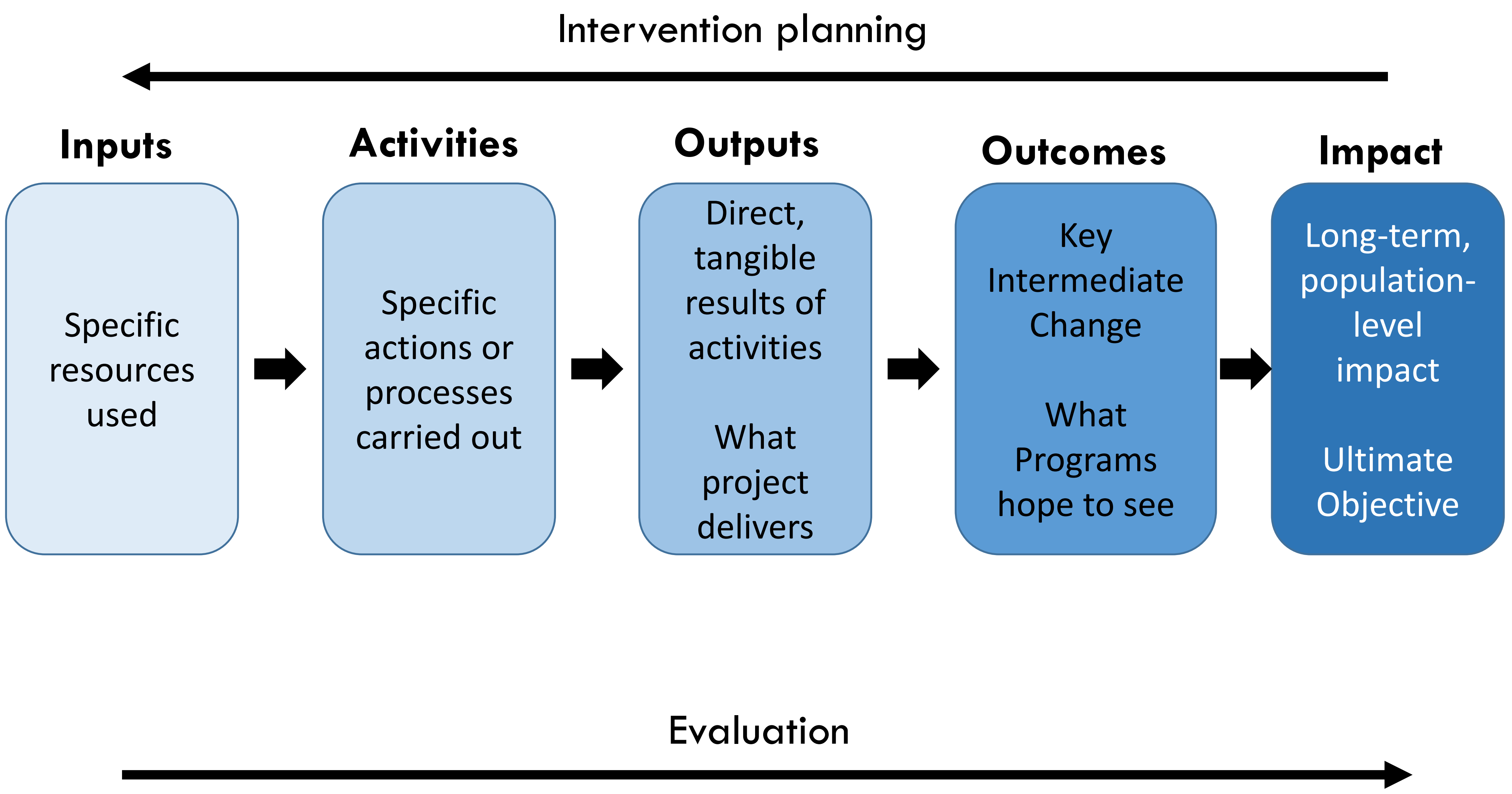

Monitoring MHM:
Measuring Progress on MHM Meeting

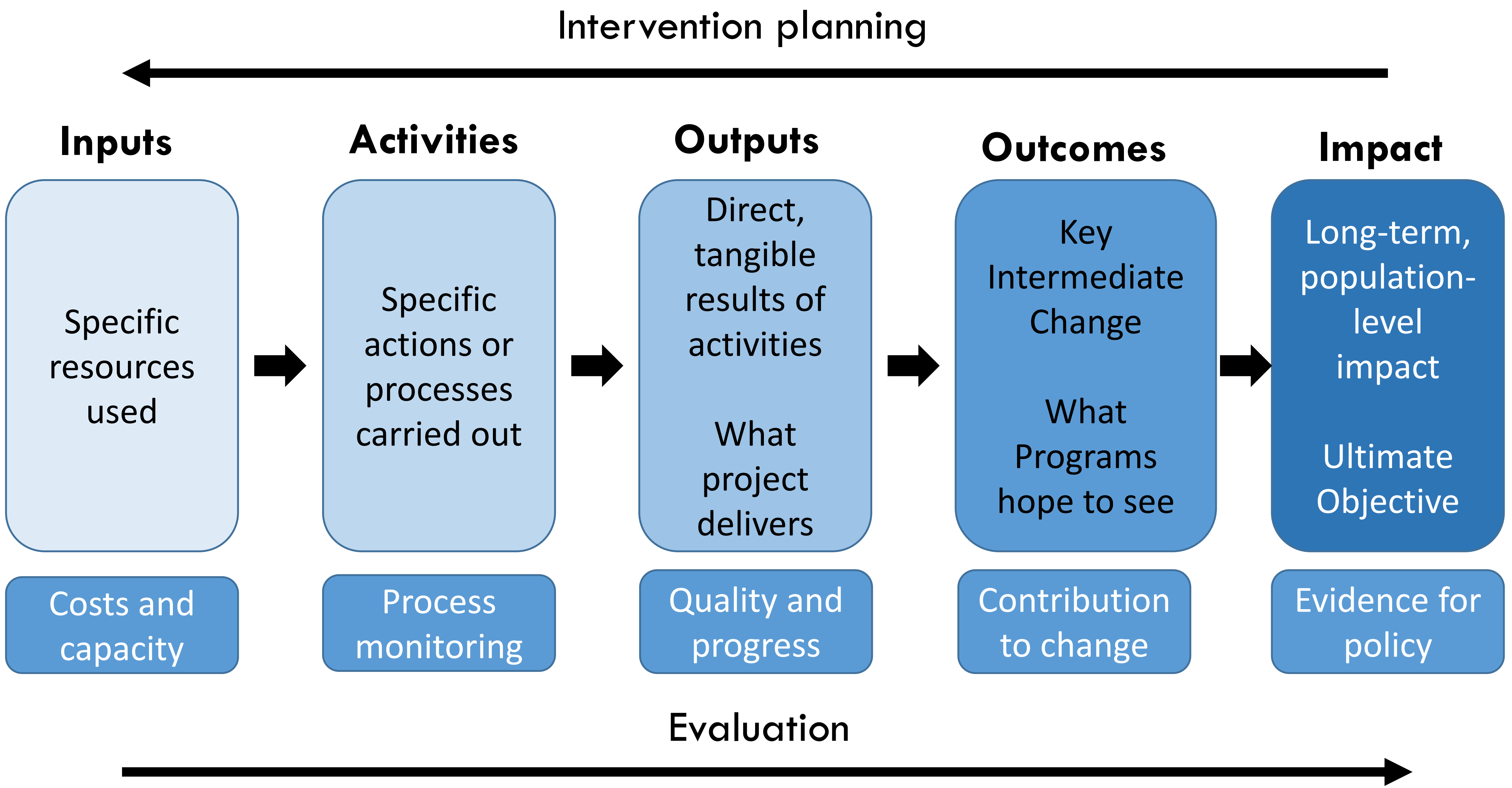

Monitoring MHM:
Measuring Progress on MHM Meeting

Illustrative Example

Multiple activities may work in concert across multiple levels toward a specific goal.

A 'package' of activities may be necessary for achieving outcomes and impact

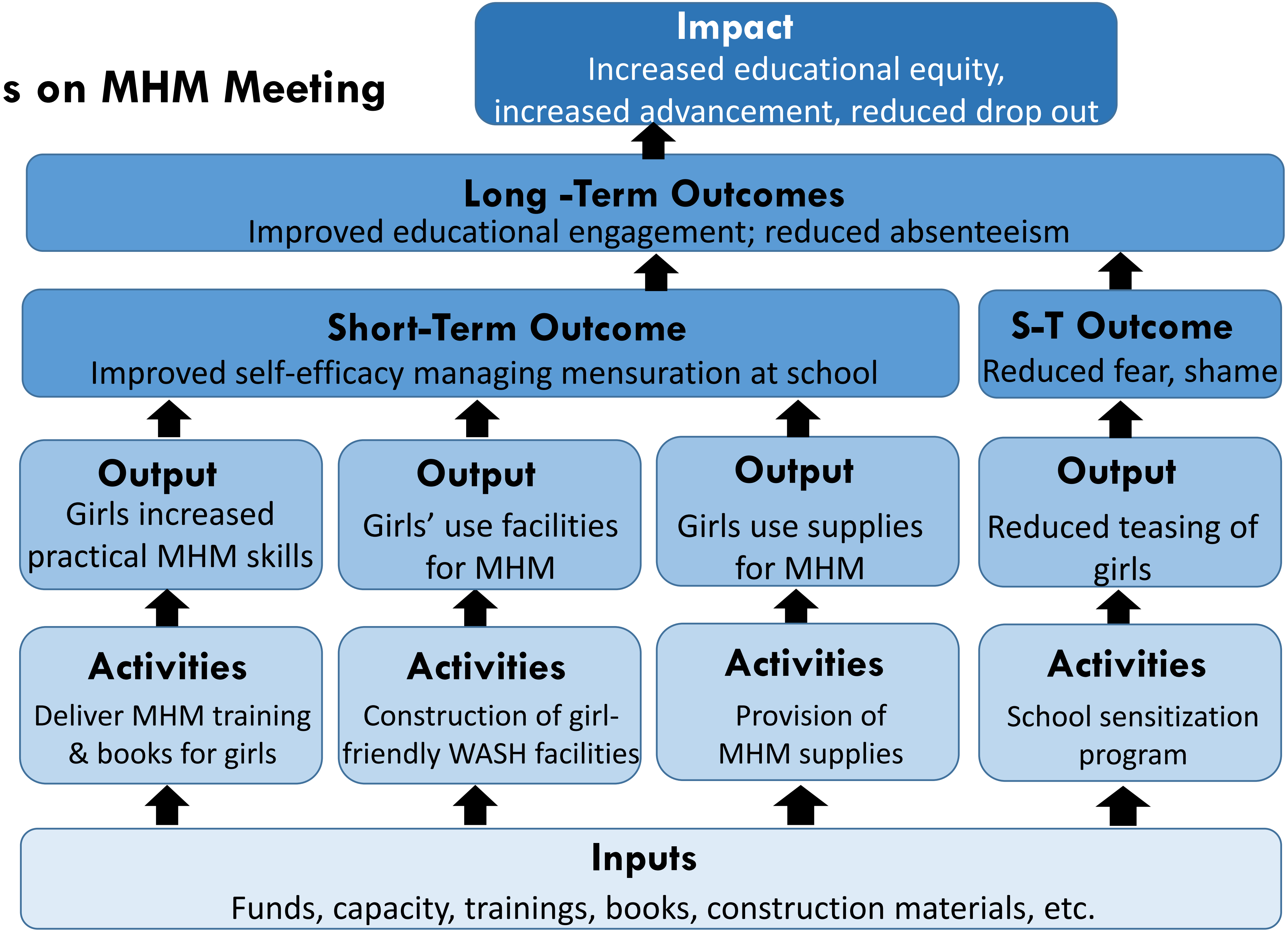

MONITORING MHM: MEASURES PROGRESS ON MHM

FLOW OF SESSIONS

1	Scene setting + understanding and linking perspectives	Identifying key area priorities and indicators	Alignment of measures from key areas with MHM
2	Review of MHM measurements and gaps	Systematic MHM measures analysis	Exploring fit for purpose and what's missing
3	Taking forward - Linking to national and global monitoring	Existing national monitoring of MHM and challenges	Next steps and dissemination

Foundational Presentation: WASH

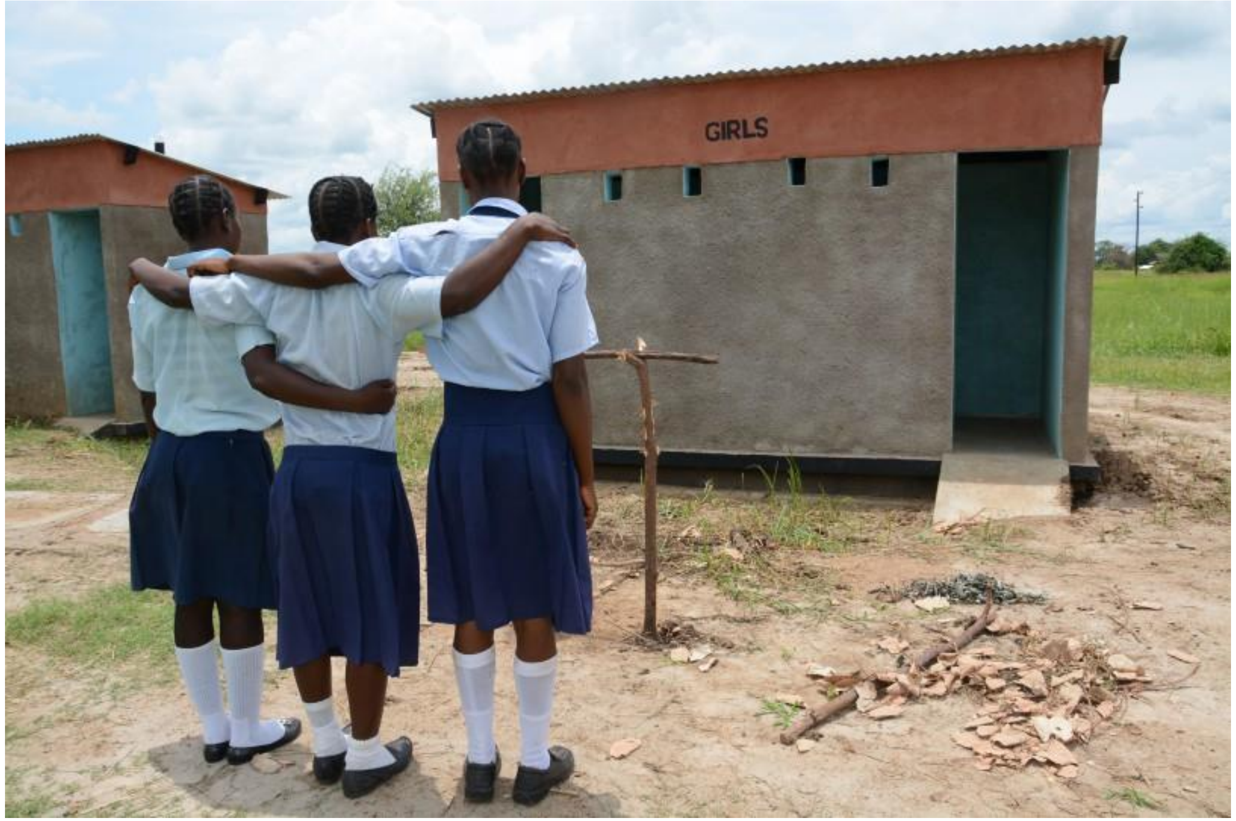

Belen Torondel 11/3/2019

LONDON SCHOOL of HYGIENE & TROPICAL MEDICINE

WASH and MHM (Enabling environment)

Water

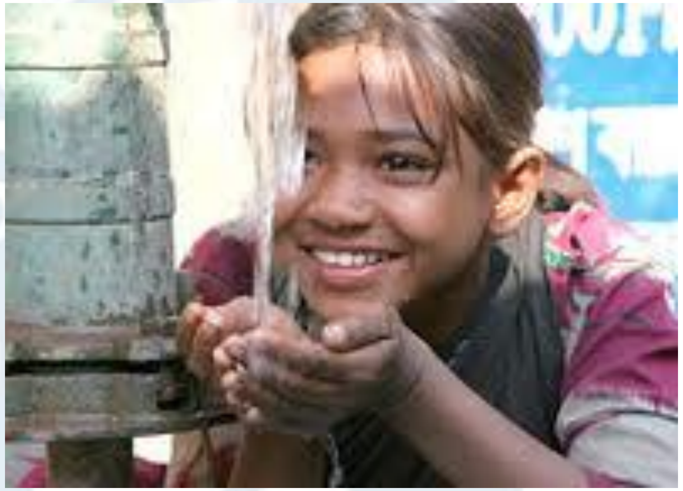

Sanitation

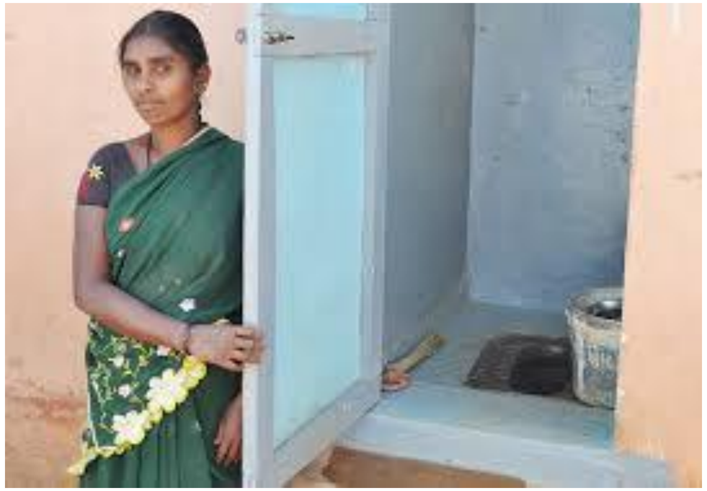

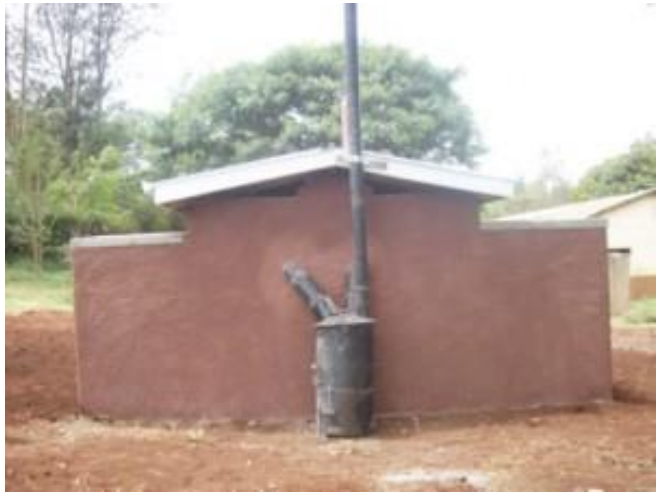

Hand-washing with soap

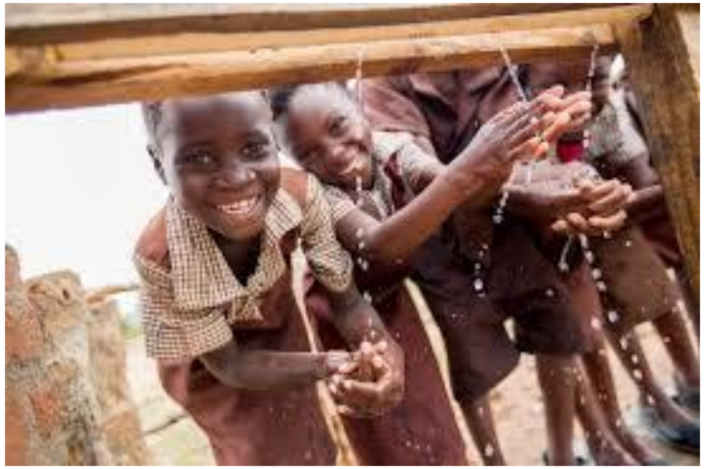

LONDON SCHOOL of HYGIENE &TROPICAL MEDICINE

Overview of the linkages of MHM with WASH:

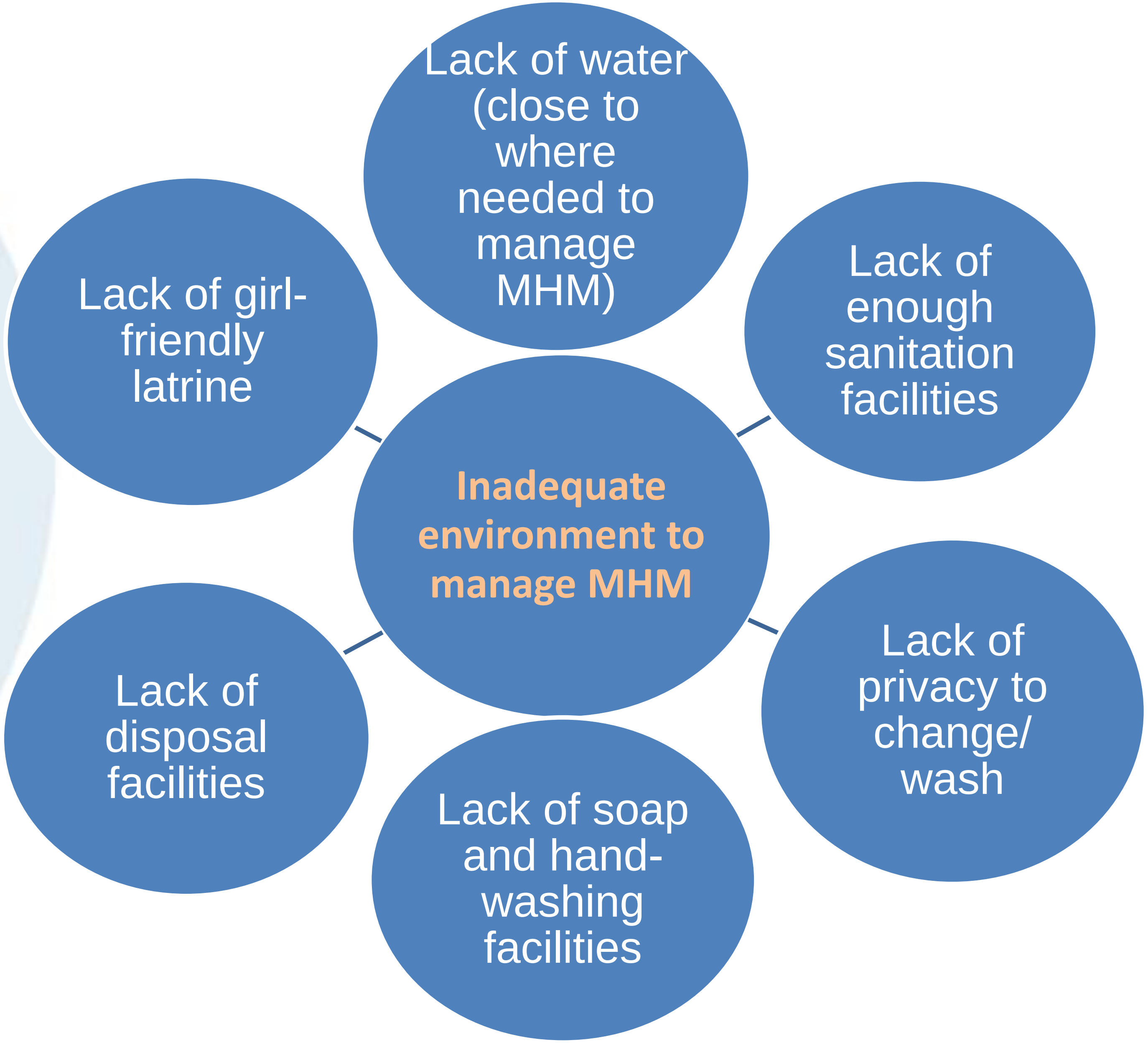

LONDON SCHOOL of HYGIENE &TROPICAL MEDICINE

Overview of the linkages of MHM with WASH

Poor WASH environment

- Education /labor participation
- Increased workload
- Urogenital infections
- Blockage of Sanitation systems
- Reduced Safety

LONDON SCHOOL of HYGIENE &TROPICAL MEDICINE

Example 1:

"Let's always use toilet: When we did not have toilet, we had to go to the ground. We felt difficulty in going to the ground. Elderly people would feel more difficulty to go to the ground. It would be more difficult if one had to go to toilet at night. Snake could sting at night. One could fall in ditch due to darkness as well. Thieves may also attack at night. That is why we have to build toilet in our houses and use them".

Ranjita - Siraha, Nepal

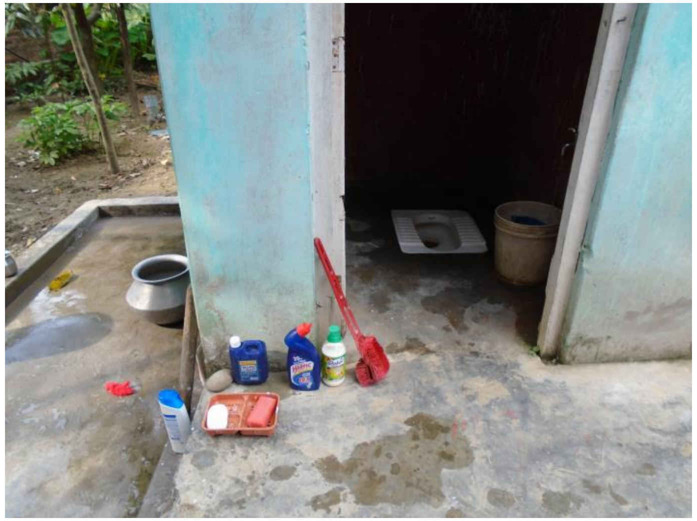

WaterAid/Ranjita

Activities	Outputs	Outcomes	Impact
Programmes to build latrine infrastructure at household level	Latrines constructed with light, locks and access to water and disposal facility	Safe and private place to manage menstruation	Reduce Risk of assault and sexual harassment

LONDON SCHOOL of HYGIENE & TROPICAL MEDICINE

Example 2:

"This is the girl's toilet of our school. We are in urgent need of MHM friendly toilet. The one we use doesn't lock properly. If someone is inside, other person has to wait outside pushing the door for her. Because of lack of latrines in our school, we have to wait in the long line." Sushma - Sindhuli, Nepal

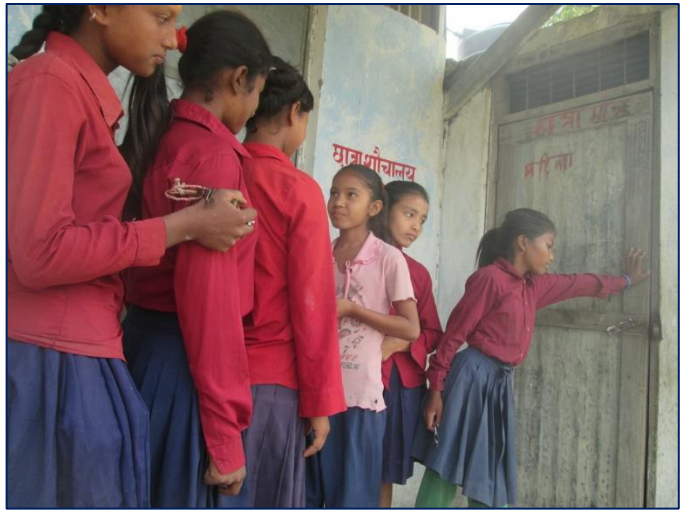

WaterAid/ Sushma

Activities

Programmes to provide WASH infrastructure to schools

Outputs

Adequate latrines with access to water, locks, disposal bins, gender separated latrines

Outcomes

Comfortable and private place to manage menstruation whilst in school

Increased self-efficacy managing menses

Impact

Increase school attendance

Increase school engagement

LONDON SCHOOL of HYGIENE &TROPICAL MEDICINE

Example 3:

"Rubbish is not seen here and there. We do not know where the rubbish is thrown. After throwing in the (rubbish collection) vehicle, those pads are not at home, and I feel relaxed. There is no need to dig a pit, bury or set fire to it…there will not be any infection…"

Morrison et al 2016 (WaterAid)

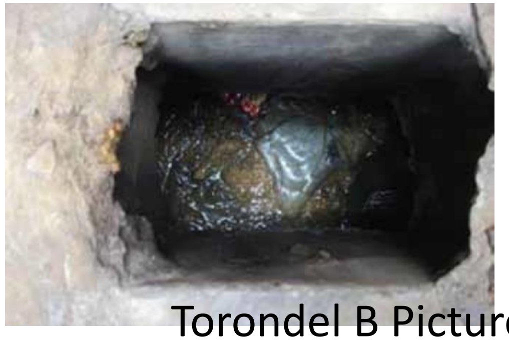

Torondel B Picture

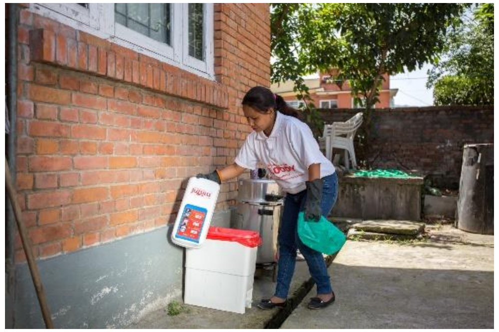

WaterAid Picture

Activities

Programmes to provide disposal facilities and services

Outputs

Appropriate disposal services for menstrual products (bins, incinerators, waste collection and treatment)

Outcomes

Reduce fill up and blockage of sanitation systems

Women and girls use disposal bins

Impact

Reduce expenses to empty sanitation systems

Women and girls experience less stress

LONDON SCHOOL of HYGIENE & TROPICAL MEDICINE

Example 4:

"During menstruation there is more use of water.. But, there is a greater tragedy that we are not allowed to touch water if we are in our menstrual cycle and the other person is fetching water. Because of which we have to wait in the line waiting for my turn until everybody's done. By the time we reach home it's already dark and sometimes it even difficult to complete our home works in the darkness." Manisha – Sindhuli Nepal

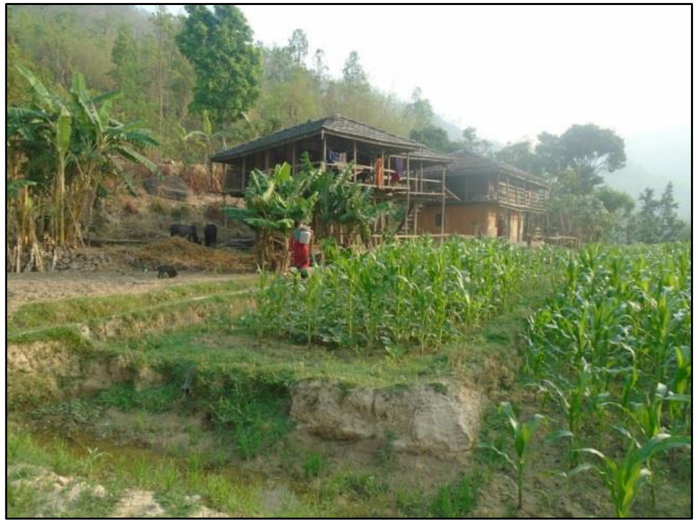

WaterAid/ Manisha Karki

Activities	Outputs	Outcomes	Impact
Programmes to provide water close to the latrines	Water close to the latrine	Reduce workload of girls and women to collect water and bring close to latrines	Increased time for other activities

LONDON SCHOOL of HYGIENE &TROPICAL MEDICINE

Girls with disability can face more barriers to access WASH during menstruation

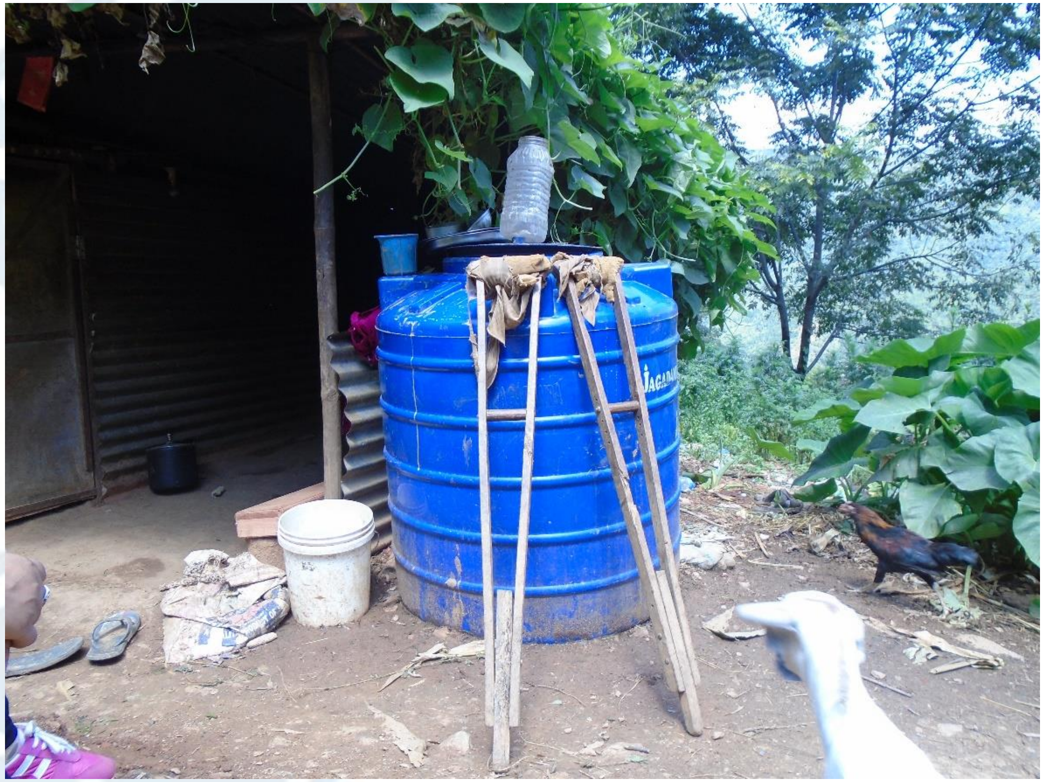

Photo credit: Sharmila Tamang

"Water issue is also there, I would have to carry water, which is difficult.

"That's my crutches and water tank. I need to use crutches to walk thus can't carry water. It's either me carrying my two legs or water... [When we don't have water] I do nothing... I just keep the [menstrual] cloth.. under my bed when I can't wash it..."

LONDON SCHOOL of HYGIENE & TROPICAL MEDICINE

Results chain (examples): WASH and MHM

Interventions	Outputs	Outcomes	Impact
Provision of WASH hardware<br><br>Provision of behaviour change intervention (i.e. to change use, maintenance practices of WASH systems)	Facilities constructed (girl friendly, sex segregated toilets; hand-washing structures; access to water)<br><br>Facilities and consumables provided (bins, water carriers systems, soap, incinerators)<br><br>Behaviour change programs delivered (for changing practices or for management and maintenance of facilities)	Increase use and maintenance of facilities<br><br>Increase frequency of menstrual material change<br><br>Increase frequency of appropriate body washing practices<br><br>Increase of appropriate cloth cleaning/ drying/ disposal practices	Improve comfort and satisfaction when managing menstruation<br><br>Improve safety<br><br>Reduce urogenital infections<br><br>Self-efficacy managing menstruation at school<br><br>Improve education broadly<br><br>Increase sustainability of WASH services

Thanks!

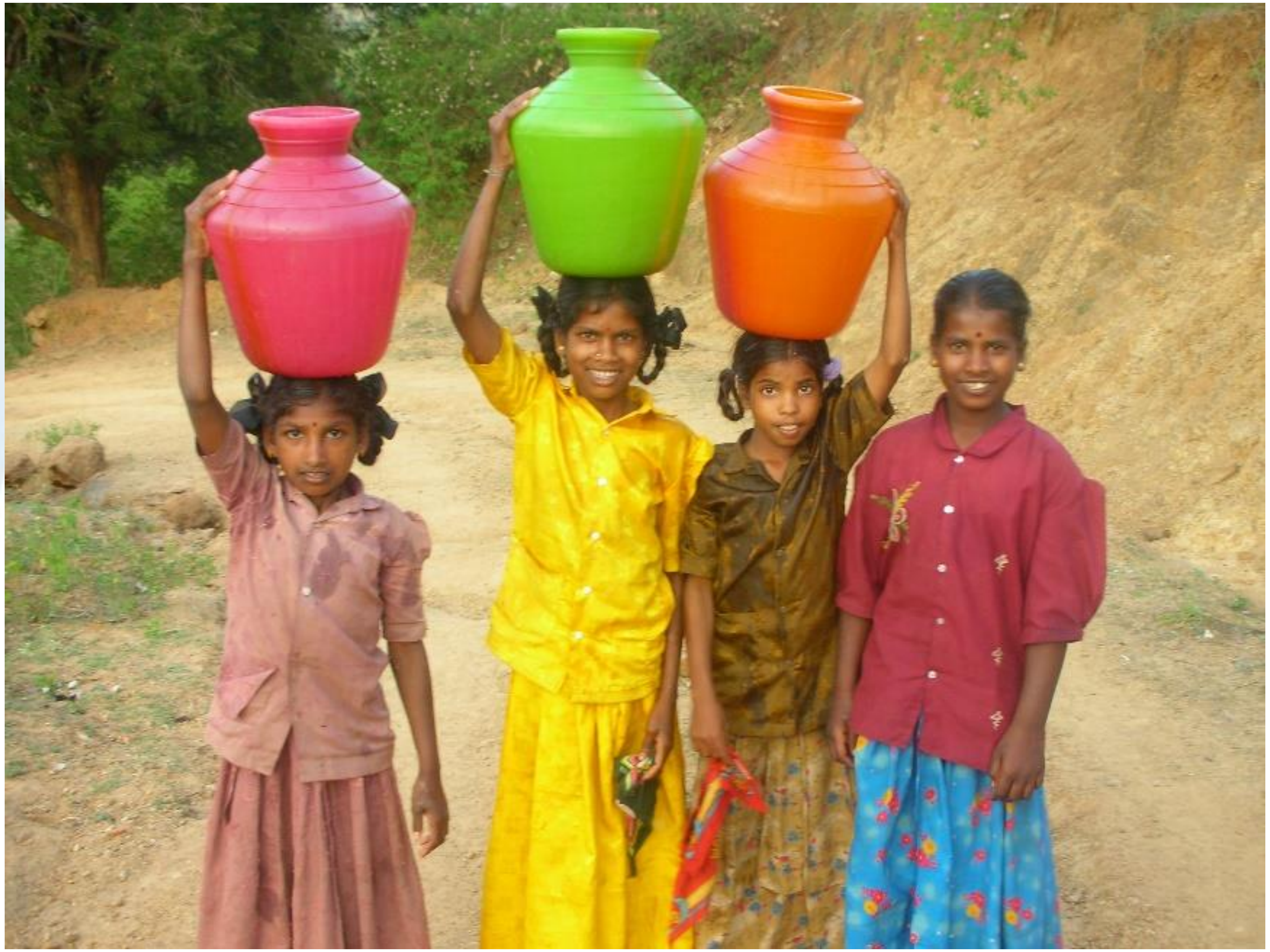

Foundational Presentation : Education

Jacquelyn Haver
March 11, 2019

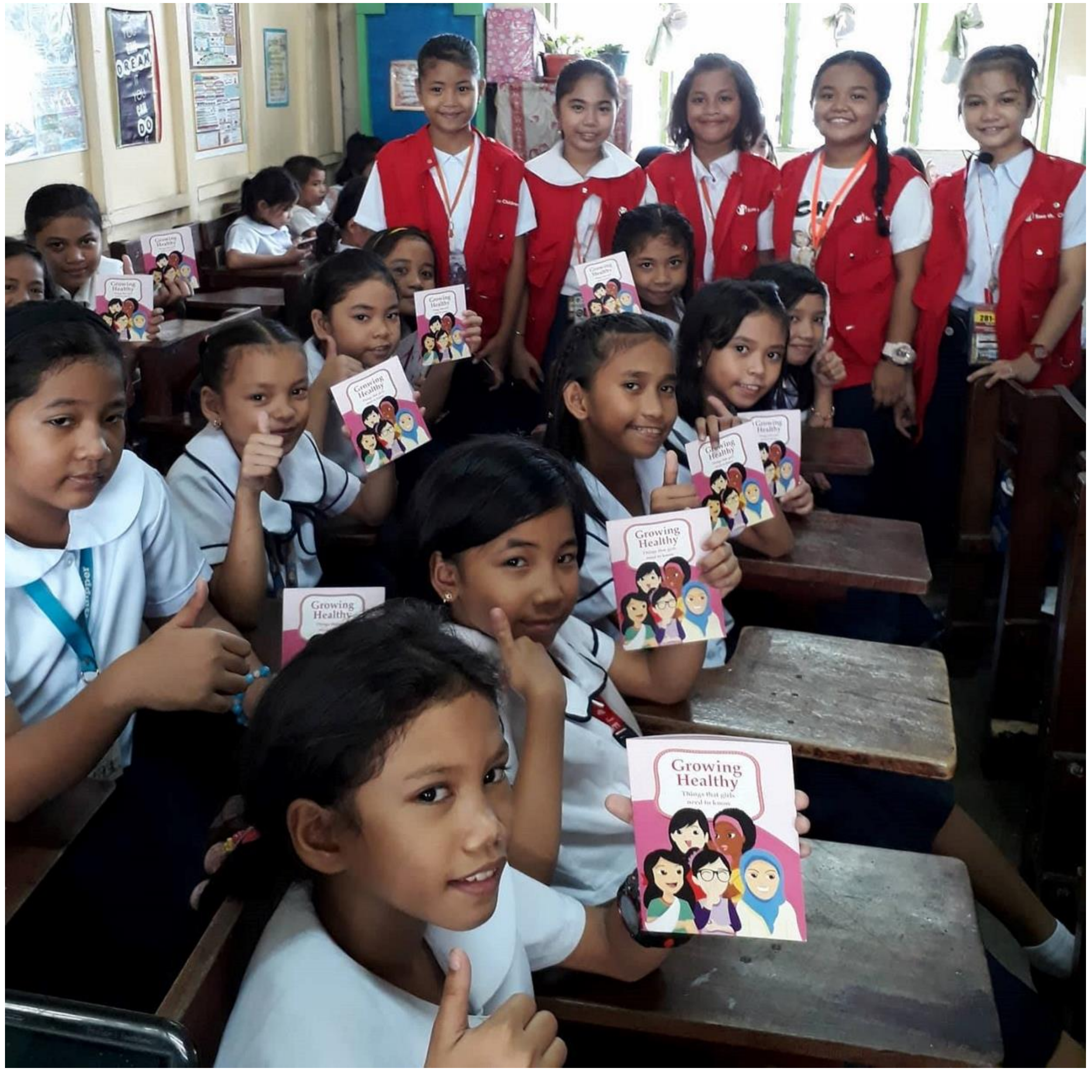

Overview of the linkages of MHM with Education:

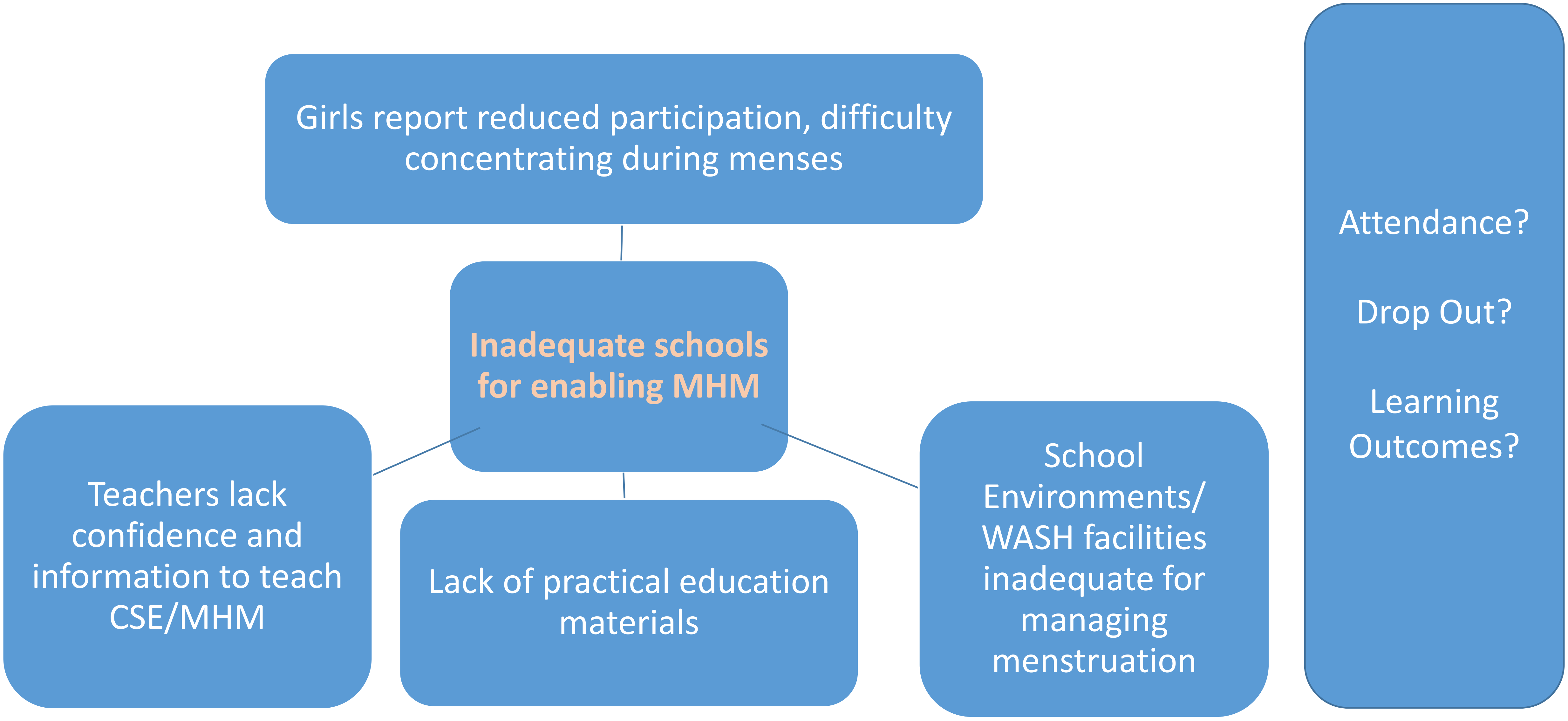

MHM and Education

What about Attendance?

- In Bangladesh girls were more likely to miss school during menstruation if they believed that their menstrual problems impacted their performance in school, attended schools that had locked toilets, or faced restrictions during menstruation (Alam et al., 2017).
- In rural Malawi, 1/3 of girls interviewed missed at least one day of school during their last menstrual period (Grant et al., 2013).
- In Uganda, high school girls and boys missed class equally, but girls were more likely to miss school during their menstrual period (Miiro et al., 2018)
- Studies evaluating the impact of material and education provision on school attendance have yielded mixed results (Oster et al., 2011; Montgomery et al., 2012; Montgomery et al., 2016; Philipps-Howard et al., 2016).

MHM IMPACTS MORE THAN SCHOOL ATTENDANCE

‘‘They do not participate – they feel tired – and their academic performance goes down. The teachers do not help the girls – if a girl has an accident or bad cramps, she will go home – the teachers will not know she has left.”

~rural schoolgirl (Sommer 2010)

MHM IMPACTS MORE THAN SCHOOL ATTENDANCE

- Northern Tanzania: the onset of menses restricts girls' ability to **participate** in social and academic activities…They often experience **shame, confusion, and fear,** as a result (Sommer, 2009).
- Bolivia, Philippines & Sierra Leone: Poor MHM negatively impacts **girls' concentration and participation** at school (UNICEF, Emory University, Save the Children, 2013)
- Kenya: girls described **difficulties engaging** in class, due to **fear** of smelling and leakage, and subsequent teasing (Mason et al, 2013)
- Reaching menarche in ignorance and in fear may weaken girls' sense of **self-confidence and competence** (Williams & Currie, 2000; Short & Rosenthal (2008); Ruble & Brooks-Gunn (1982)

Theory of change: Education

Interventions	Outputs	Outcomes	Impact
Provision of practical MHM learning materials through CSE<br><br>Provision of training curricula on MHM including sensitivity approaches (CSE link)<br><br>Provision of teacher training on MHM	Number of<br>- MHM learning materials distributed<br>- MHM trainer materials & resources developed<br>- Quality trainings provided to teachers on MHM<br>- Trainings provided to students on MHM	Increased knowledge about MHM<br><br>Reduced Stigma & misconceptions about MHM<br><br>Reduced stress	Reduced embarrassment<br><br>Reduced teasing<br><br>Improved Attendance completion promotion<br><br>Improved education learning outcomes

Example 1: Classroom Engagement/Participation

Challenges:

- Girls report challenges with concentration/participation in the classroom during menses
- Worried about being teased about stains
- Experiencing menstrual pain

"She pays no attention to lessons. She is different. She is thinking other things in class….we think about leaking, stains, when we sit in the chair one hour until the lesson is over… hoping that the teacher will not ask us to stand up. We sit without understanding, and if a teacher tells us to stand up and repeat what she has just explained, we do not know what to answer."

Girl Student, Kyrgyzstan

Inputs

Emergency menstrual product supplies available in schools

→

Activities

Schools develop system for girls to access menstrual products

→

Outputs

Girls access & use emergency menstrual product supplies at schools

→

Outcomes

Support provided through schools

Increased participation

→

Impact

Increased attendance / completion / promotion?

Improved education outcomes (reading/literacy numeracy, educational engagement) ?

Reduced drop out?

Example 2: MHM Information/Knowledge Gap

Challenges:

- Girls & boys lack practical & accurate MHM education/information
- Teachers lack knowledge, confidence, sensitivity & training to teach MHM/CSE

"For me as a male teacher that's the most difficult. How can you teach that? In our society, it's difficult to just tell them 'wash your...' For me, it's awkward if a male teacher will say that. Even for the students. It's because in our culture, we're embarrassed, unless there is professional training for the male teachers. It will turn into laughter."
Male Teacher, Philippines

Inputs	Activities	Outputs	Outcomes	Impact
MHM Education for girls & boys	MHM education materials & teacher training developed for schools	Delivery of Teacher training<br><br>Delivery of MHM education	-Girls utilize MHM practical guidance & knowledge<br>-Reduced misconceptions about menstruation<br>-Increased participation<br>-improved/changed boys behavior to MHM	Increased attendance / completion / promotion?<br><br>Improved education outcomes (reading/lit numeracy, educational engagement) ?<br><br>Reduced drop out?

Example 3: Emotional School Environment & MHM

Challenges:

- Lack of social support from teachers & peers
- Lacking empathy/awareness for girls' challenges with menstruation
- Boys & girls report teasing girls about menstruation

"Maybe because they don't know what girls are feeling, that's why they tease them, and sometimes because of their smell." *Boy, Philippines*

"…One boy noticed… and took the pad from her bag and started to show everyone. The girl cried, and he was scolded by the teacher. After that, we're afraid to bring …[pads] to our school, because they might tease us."
Girl, Kyrgyzstan

**Inputs**		**Activities**		**Outputs**		**Outcomes**		**Impact**
Sensitivity training for teachers on MHM<br><br>Peer & teacher support for girls	➡	School child clubs include MHM<br><br>School rules: teasing/ bullying	➡	-Delivery of sensitivity trainings for teachers<br>-Child clubs<br>-School rules includes MHM	➡	Support provided at school (e.g. social/teachers)<br><br>Girls use clubs to access support<br><br>Increased self efficacy<br><br>Reduced stress	➡	Improved social and emotional environment for menstruating girls<br><br>Increased attendance / completion / promotion?

Example 4: Physical School Environment Inhibits MHM

Challenges:

- WASH facilities in schools lack privacy, functionality & disposal
- School budget for managing school WASH insufficient
- Lack of school governance for managing school WASH

"Older girls report that school latrines are not sanitary for menstrual hygiene. There is no water source in or near latrines with which to clean adequately, no disposal for sanitary napkins, and the lack of privacy makes them 'ashamed' to engage in proper menstrual hygiene."
Girl Student, Kyrgyzstan

Inputs

Budgeted WinS

WinS O&M

→

Activities

Training on WinS budgeting

Training on WinS O&M

→

Outputs

Delivery of WinS budget trainings

Delivery of WinS O&M training

→

Outcomes

Sustainability of services (e.g. infrastructure operations & maintenance, soap)

User uptake & satisfaction with facilities

→

Impact

Improved self-efficacy to manage menses at school (for teachers & students)

Increased attendance?

Foundational Presentation : Psychosocial

Social and psychological interventions are…

"actions intended to modify processes and systems that are social and psychological in nature (such as cognitions, emotions, behaviours, norms, relationships, and environments) and are hypothesised to influence outcomes of interest."

Grant et al. 2018 (CONSORT-SPI extension)

The biopsychosocial model (of health) (Engel, 1977)

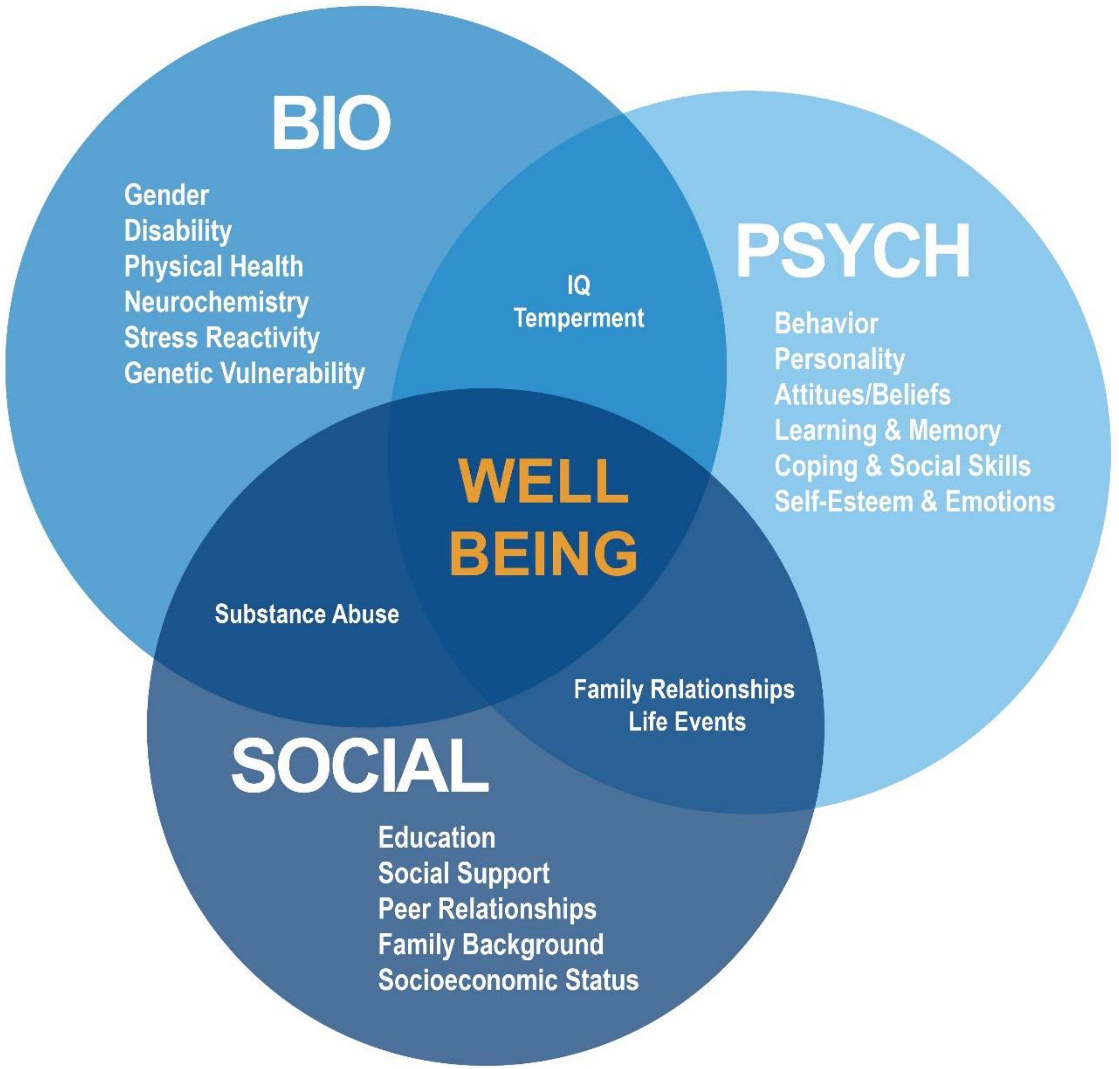

Socio-ecological framework (Bronfenbrenner, 1979)

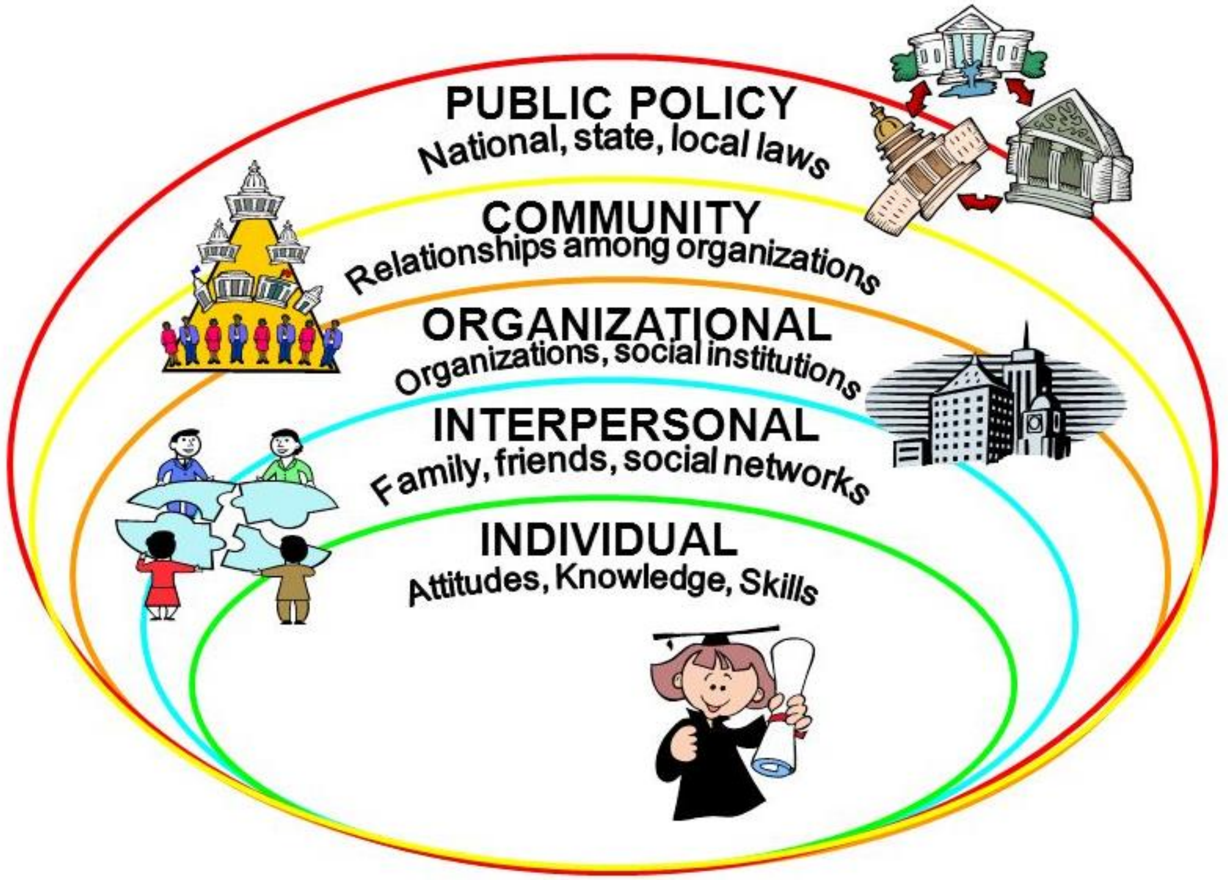

Identified psychosocial determinants include

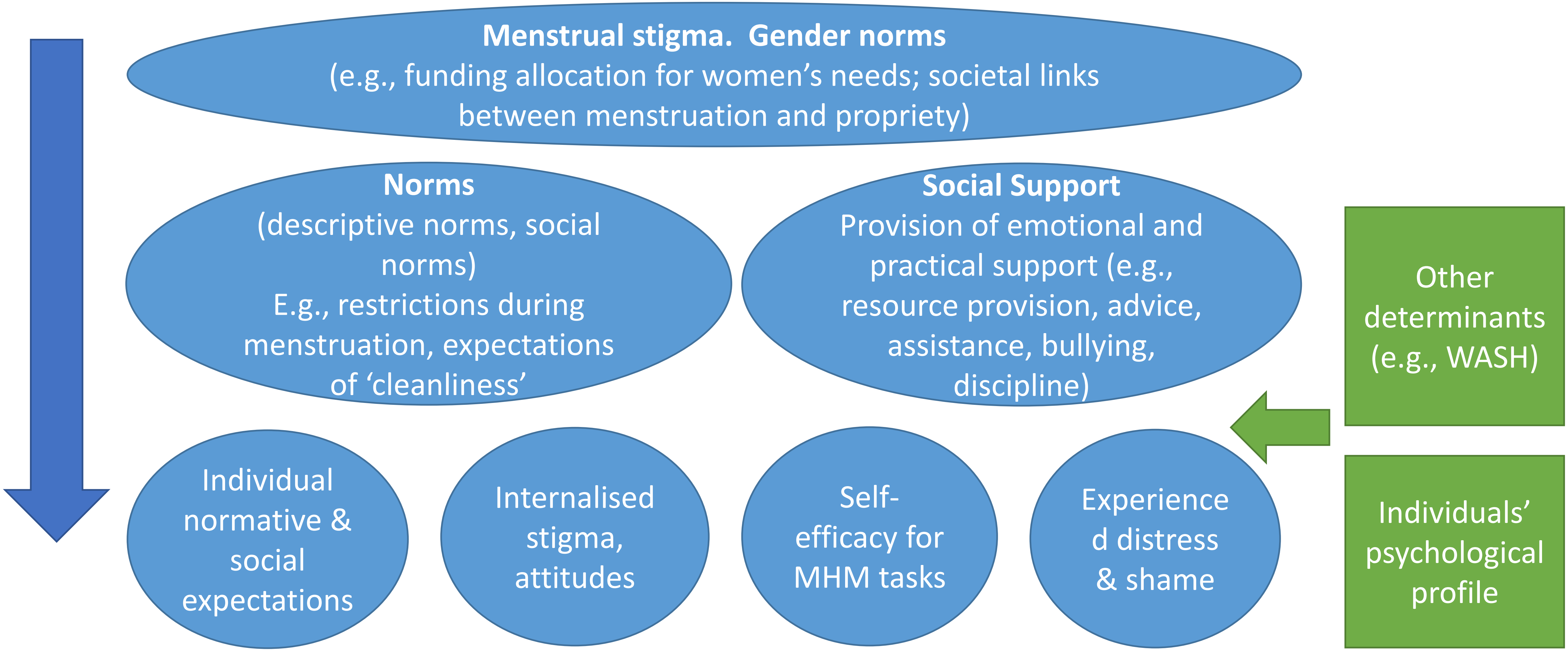

Impacts on health & participation

Psychosocial outcomes and impacts:

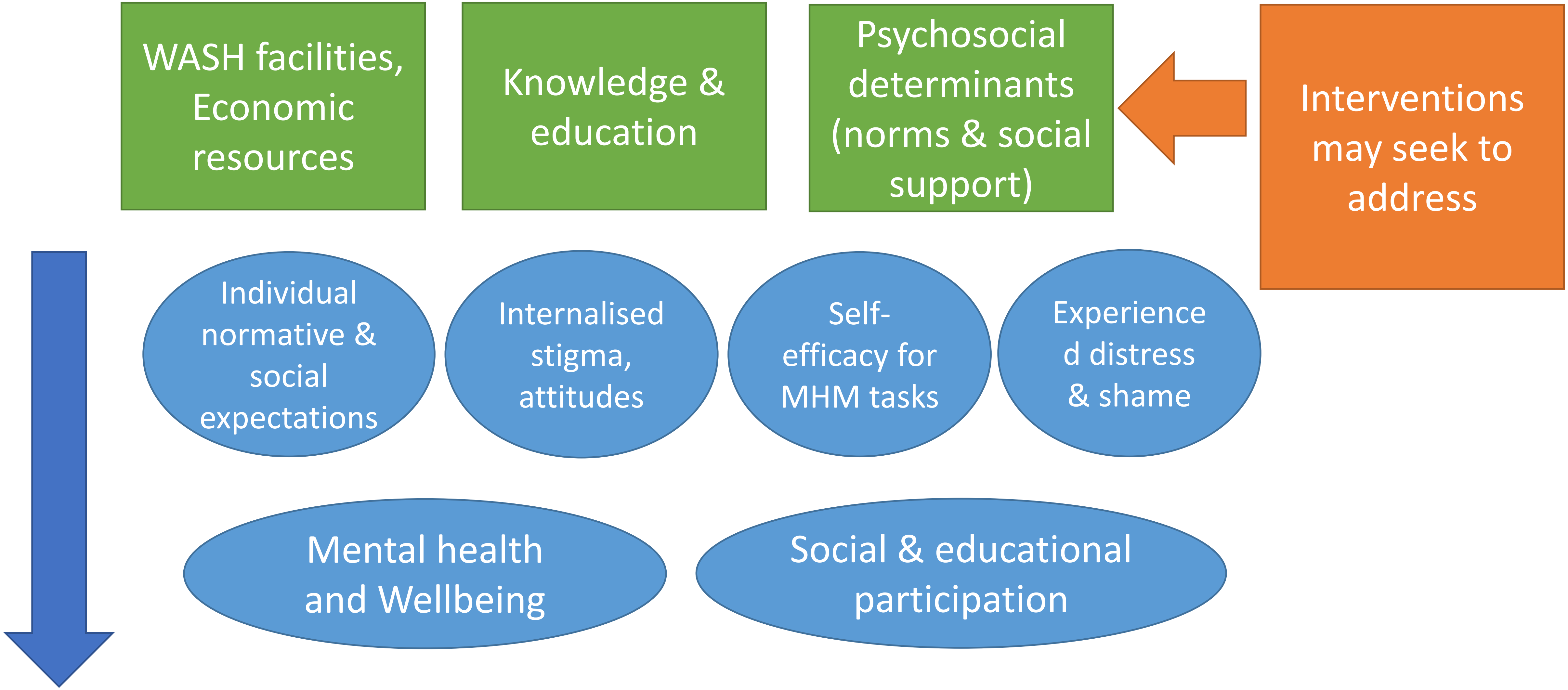

Example 1: Social Support – peers, parents, teachers

"[I talk to] friends, [and] they help to explain about how to use sanitary pad properly, product of hygiene, and give sanitary pad because they understand about the issue and consider me their best friend." (Daniels, 2016)

"A teacher might be teaching and then a friend stands up when she has soiled her clothes, you advise her to go out she leads the way and you follow to shadow her up. You get her other clothes to put on." (Hennegan, 2017)

"You know, especially the male teachers, I don't think they understand that when you mess yourself up it is by accident." (Lahme, 2016)

Girls are able to seek information and support from parents, peers, teachers

→

Support sources provide: information, protection, resources, emotional support

→

Increased transmission of information and support

Girls more willing to seek assistance

→

Increased self-efficacy

Reduced anxiety around menstruation

Increased capacity to enact hygiene behaviours

→

Increased attendance / completion / promotion at school

Increased self-esteem and reduced anxiety

Example 2: Menstrual stigma (norms of silence)

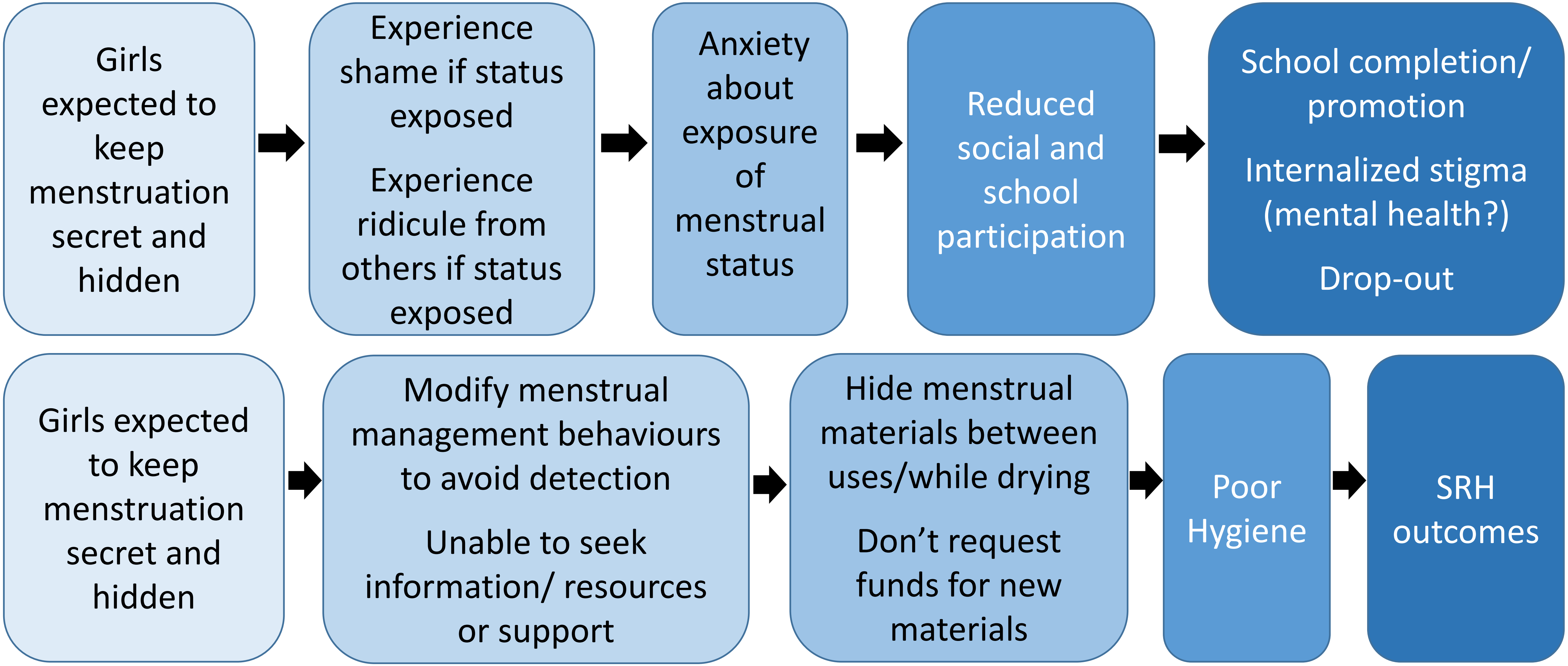

Example 3: Explicit behavioural restrictions

Restrictions may influence:

- Materials and disposal
- Fears of exposure of menstrual blood
- Religious and social participation
- Food
- Household tasks
- Interactions with males

"I have heard from nearly everyone; teacher, mother, aunt and grandmother, that during my period, I should not eat cold foods, foods that produce gas, pickles, yogurt and milk because they cause stomach-ache. But I have not experienced it because I have not eaten them. In other words, I have always observed these suggestions. Well (smiling), if my mother sees me eat these things during my period, she will quarrel with me" (Morowatisharifabad, 2018)

Girls restricted from foods, behaviours during menstruation

→

Restrict food intake

Embarrassment from avoiding religious activities in school

Hiding menstrual blood or modified disposal practices

→

Fatigue

Negative attitudes towards menstruation and conceptualization of the self as 'dirty'

→

Reduced social and educational participation

Punishments for deviations

→

Attendance / completion / promotion at school

Self-esteem and [generalized] anxiety?

MHM and Mental Health

Participants used language like *"feeling bad,"* feeling *"stressed",* or *"fearful"* and *"wanting to cry"* to describe the emotional distress they experienced. *(Crichton 2013)*

While being secluded, girls reported being bored, lonely and depressed: *"I was bored sitting there alone. I felt like no one loved me…I felt like crying…"* (Morrison 2016)

"During my first menstruation I was shocked and embarrassed. Generally, whenever I have it, I think that I am below humans, depressed, eh … I hate being female; I assumed it as a disease....." (Tegegne 2014)

"I always feel, you are lonely all the times, you feel the blood coming out rapidly in drops. During that time you become tired, you feel headache and you become restless and you cannot work, even if you do some chores, you are just dull." (Mason 2013).

- Mental health (assoc. risk behaviours)
- Psychosocial wellbeing
- Premenstrual dysphoric disorder (PMDD) – DSM-5

Methods Available

- Questionnaire and survey methods
  - Established and adapted mental health and wellbeing measures
  - Specific measures for menstrual stigma, stress, self-efficacy, attitudes
- Diagnostic interviewing
- Bio-indicators for stress (e.g., hair cortisol)
- Hospital/health records: self-harm, violence

Results chain (examples): Psychosocial approaches

Interventions	Outputs	Outcomes	Impact
Parental and community education (incl. males)<br><br>Social norms approaches<br><br>Behaviour change interventions<br><br>Mothers and girls' clubs aimed at increasing communication and social support	Number of:<br>- Support groups established<br>- Informational/ attitude campaigns at community/ social group level<br>- Supportive school policies (toilet use during class, provision of materials, uniforms)	Increased social support (peers, teachers, parents)<br><br>Reduced distress<br><br>Improved self-efficacy for MHM tasks<br><br>Changed social norms/ expectations of behaviour during menstruation<br><br>Reduced bullying/shaming	Improved mental health?<br><br>Improved self-esteem?<br><br>Participation in social activities and education<br><br>Reproductive health decision making/ help-seeking

Foundational Presentations:

Sexual and Reproductive Health

Penelope Phillips-Howard
Liverpool School of Tropical Medicine

Monitoring MHM: Measuring Progress on Menstruation

Geneva, March 11th 2019

Sexual and reproductive health linkages: WHO framework 2017

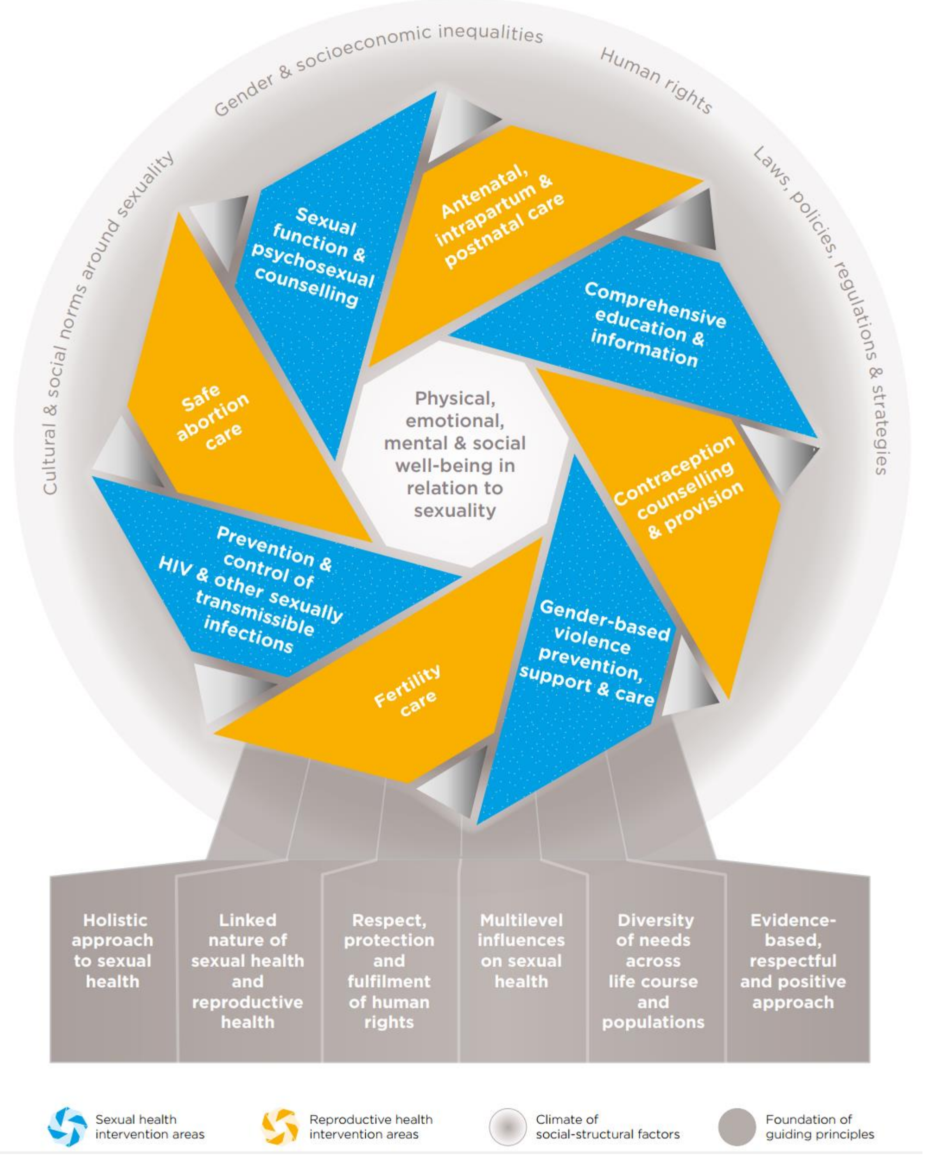

Blue ribbon: Sexual Health

- Comprehensive information/education
- Gender-based violence prevention, support and care
- Prevention and control HIV, STI
- Sexual function, psychosexual counselling

Orange ribbon: Reproductive Health

- Antenatal, intrapartum, postnatal care
- Contraception counselling, provision
- Fertility care
- Safe abortion care

Grey: social structural factors

- Cultural and social norms
- Gender and socioeconomic inequalities
- Human rights
- Laws, policies, regulations, strategies

Overview

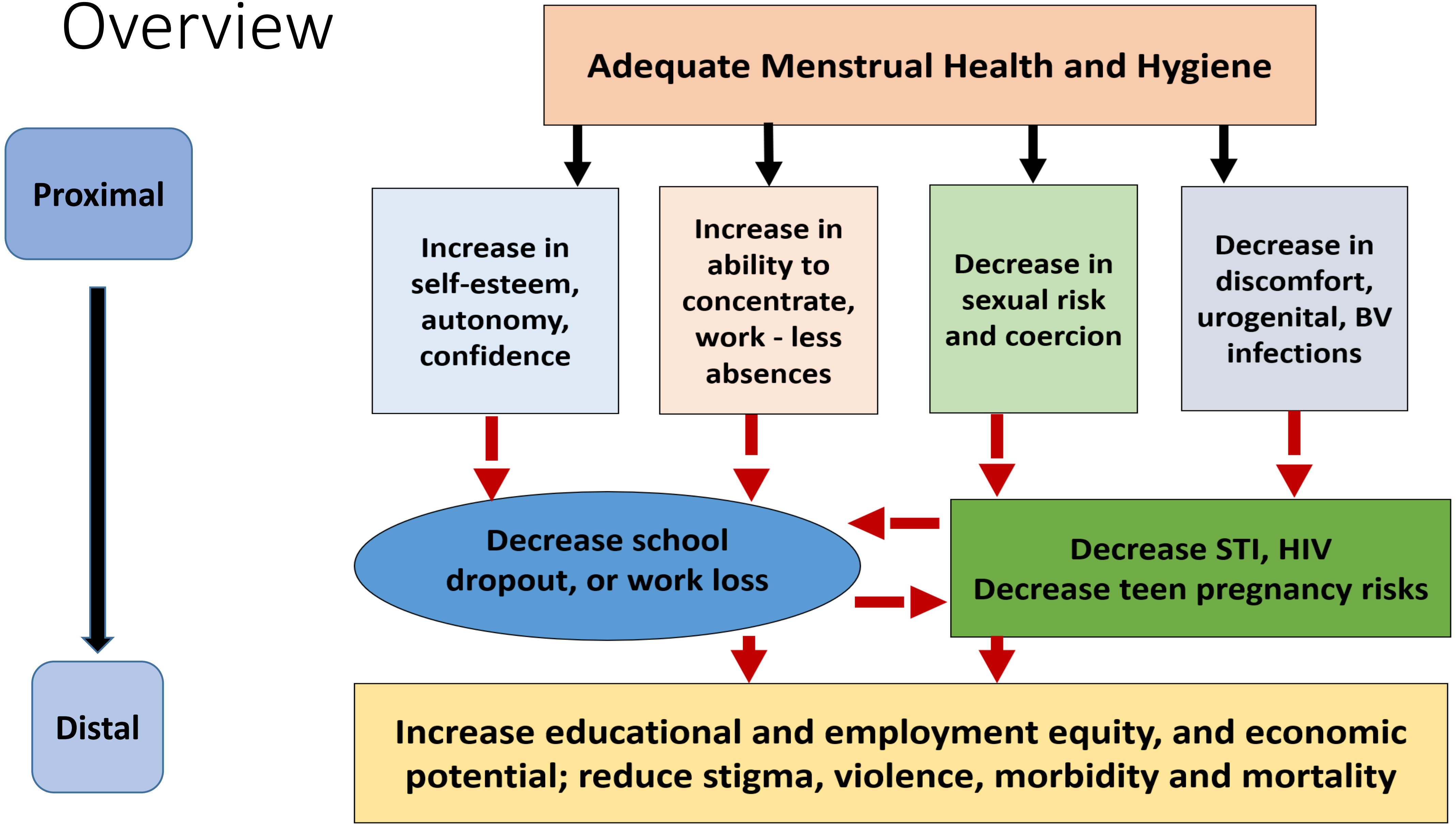

Proximal
Distal
Adequate Menstrual Health and Hygiene
Increase in self-esteem, autonomy, confidence
Increase in ability to concentrate, work - less absences
Decrease in sexual risk and coercion
Decrease in discomfort, urogenital, BV infections
Decrease school dropout, or work loss
Decrease STI, HIV
Decrease teen pregnancy risks
Increase educational and employment equity, and economic potential; reduce stigma, violence, morbidity and mortality

Example 1: Puberty knowledge and SRH

LSTM
LIVERPOOL SCHOOL OF TROPICAL MEDICINE

No CSE / SRH education
Mis-information
Unable to negotiate sex
Social norms, peer pressure

Earlier age sexual debut
More frequent sex
Increase number partners
Psychosocial / stress

Lack knowledge → Poor decision making → Increased sexual risk → STI or pregnancy → Drops out

Maternal complications
Neonatal risks
High fertility
Early marriage
IPV risks
HIV-STI

Schoolgirls:
He [uncle] told me that people start menstruating at the age of eight.
Then he also told me not to allow myself to be deceived by men because I can get pregnant at that time' **(girls of) *'a tender age they do not know when they do it [sex]'.***
(If use contraceptive)*'will not be able to give birth.'*

Example 2: Pads for sex and SRH

Type of materials used
Leakage, odor, fear
Teasing / humiliation
Psychosocial / stress

Age discordant partners
More frequent sex
Increase number partners
Unable to negotiate contraception

Poverty → Stress / pain using impromptu materials → Sex for pads → Pregnant → Difficult to stay in school → Drops out

Maternal complications
Neonatal risks
High fertility
Early marriage
IPV risks
HIV-STI

Mother of schoolgirl:
'because of lack of pads, because their parents cannot manage to buy them what to use, they go to other people like boyfriends to buy them, sometimes they have sex; by that time she is going to drop out due to pregnancy.'

Example 3: Pads for sex and SRH

Type of materials used
Leakage, odor, fear
Teasing / humiliation
Psychosocial / stress

Age discordant partners
More frequent sex
Increase number partners
Unable to negotiate safe sex

Poverty → Stress / pain using impromptu materials → Sex for pads → Increase sexual exposures → Increase risk STI → Increase risk HIV → Chronic ill health Lower employment opportunities

Schoolgirls:
'You pay with your vagina.'
'You need help, you just engage yourself into sex.'

Example 4: Poor quality materials and SRH

LSTM
LIVERPOOL SCHOOL OF TROPICAL MEDICINE

Type of materials used
Leakage, odor, fear
Teasing / humiliation
Psychosocial / stress

Poor menstrual hygiene
Water quality poor
Unable to clean/dry materials
Symptoms under-reported

Poverty → Poor hygiene with impromptu materials → Sores micro-trauma vag pH → Change in microflora → Increase risk bacterial vaginosis →

Maternal complications
Neonatal complications
STI risk
HIV risk

Schoolgirls:
'Your thighs get irritated, you can't even walk.'
'you start itching it turns to a wound.'
'You can develop a stench…when there is no soap, you become stressed up because you don't know how you will wash them.'

Results chain (examples): Sexual and reproductive health

Interventions	Outputs	Outcomes	Impact
SRH / hygiene education<br>Access to cheap effective products<br>Safe and hygienic WASH and products<br>Social norms approaches<br>Behaviour change interventions<br>School environs/rules gendered, rights driven	- High quality edu; improved SRH knowledge<br>- Improved WASH access, privacy, facilities<br>- Access to cheap, effective products<br>- Safely use products<br>- Access quality WASH<br>- Supportive school policies<br>- Pregnant girls protected rights	Reduced distress<br>Reduced bullying/shaming<br>Improved ability to negotiate safe sex<br>Reduced transactional sex; greater age-concordant partners; reduced # partners<br>Reduced absenteeism; grade repetition; improved performance	Improved wellbeing / QoL<br>Reduced dropout<br>Increased employment opportunities<br>Reduced pregnancies<br>Reduced maternal morbidity/mortality<br>Reduced STI/HIV<br>Reduced child marriage<br>Reduced poor mental health outcomes (self harm; violence, suicide)

Foundational Presentation:

Gender

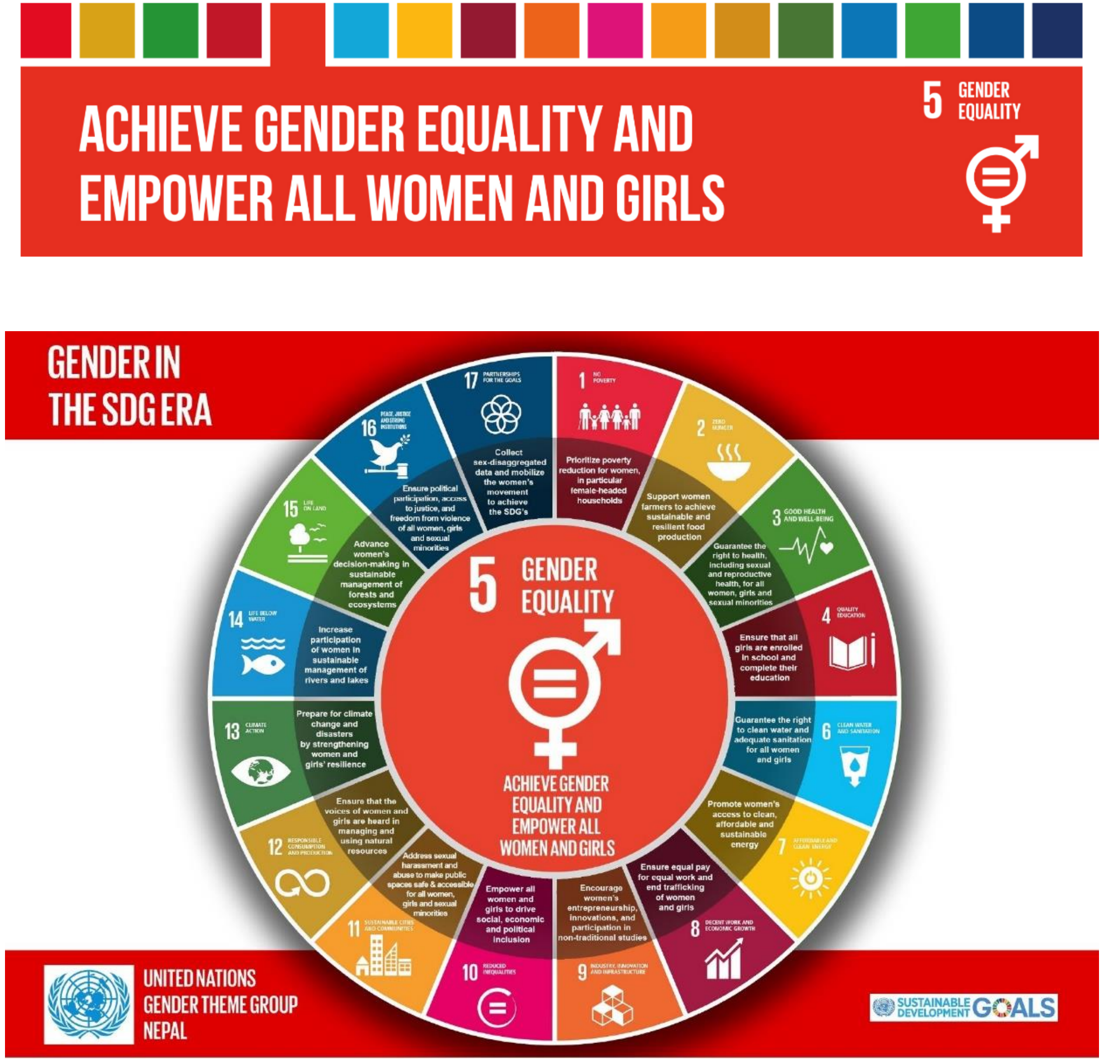

https://www.who.int/gender-equity-rights/understanding/gender-definition/en/

Gender

Refers to **the socially constructed** characteristics of women and men (e.g. norms, roles & relationships).

It varies from society to society and **can be changed**.

While most people are born either male or female, they are **taught appropriate norms and behaviours**.

When individuals do not "fit" established gender norms they often face **stigma, discriminatory practices or social exclusion, which adversely affect health.**

https://www.who.int/gender-equity-rights/understanding/gender-definition/en/

Empowerment

of women and girls is the expansion of choice and strengthening of voice through the transformation of power relations, so women and girls have more control over their lives and futures.

It is both a process and an outcome.

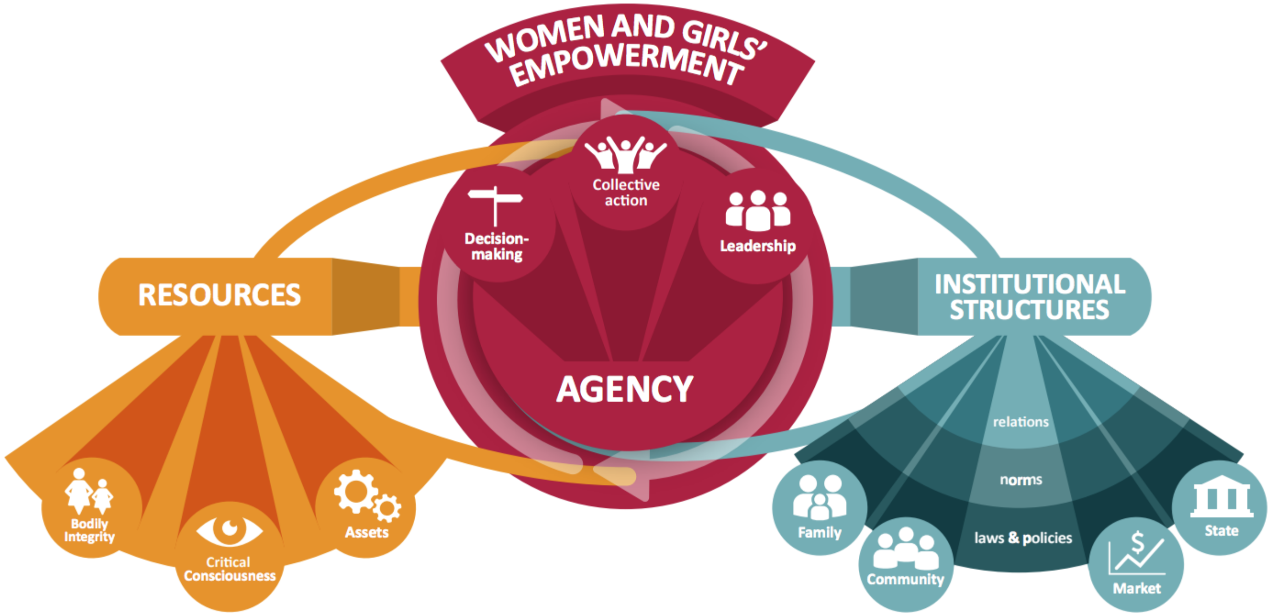

Anouka van Eerdewijk, Franz Wong, Chloe Vaast, Julie Newton, Marcelo Tyszler & Amy Pennington (2017). White Paper: A Conceptual Model of Women and Girls' Empowerment. Amsterdam: Royal Tropical Institute (KIT).

Empowerment

of women and girls is the expansion of choice and strengthening of voice through the transformation of power relations, so women and girls have more control over their lives and futures.

It is both a process and an outcome.

Disempowerment and gender inequality

manifest themselves in **unequal distribution of resources** and

women and girls' **lack of control over their bodies and low self-esteem**,

combined with **biased laws and policies** and **discriminatory gender norms and practices.**

Women and girls' lack of control over their lives arises as a result of **gender relations of power** that are based in patriarchal hierarchies and gender ideologies of male dominance and privilege.

Anouka van Eerdewijk, Franz Wong, Chloe Vaast, Julie Newton, Marcelo Tyszler & Amy Pennington (2017). White Paper: A Conceptual Model of Women and Girls' Empowerment. Amsterdam: Royal Tropical Institute (KIT).

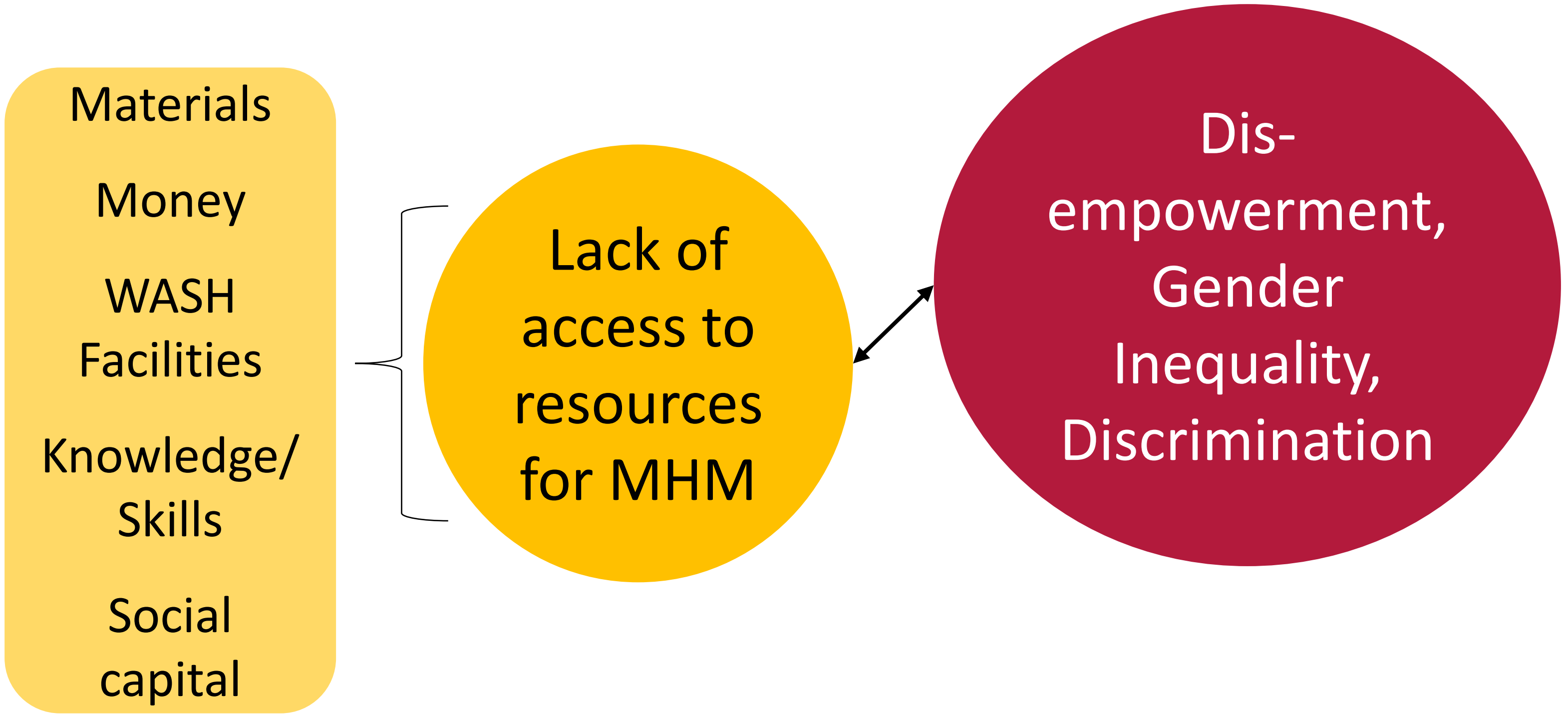
Materials
Money
WASH Facilities
Knowledge/ Skills
Social capital
Lack of access to resources for MHM
Dis-empowerment, Gender Inequality, Discrimination

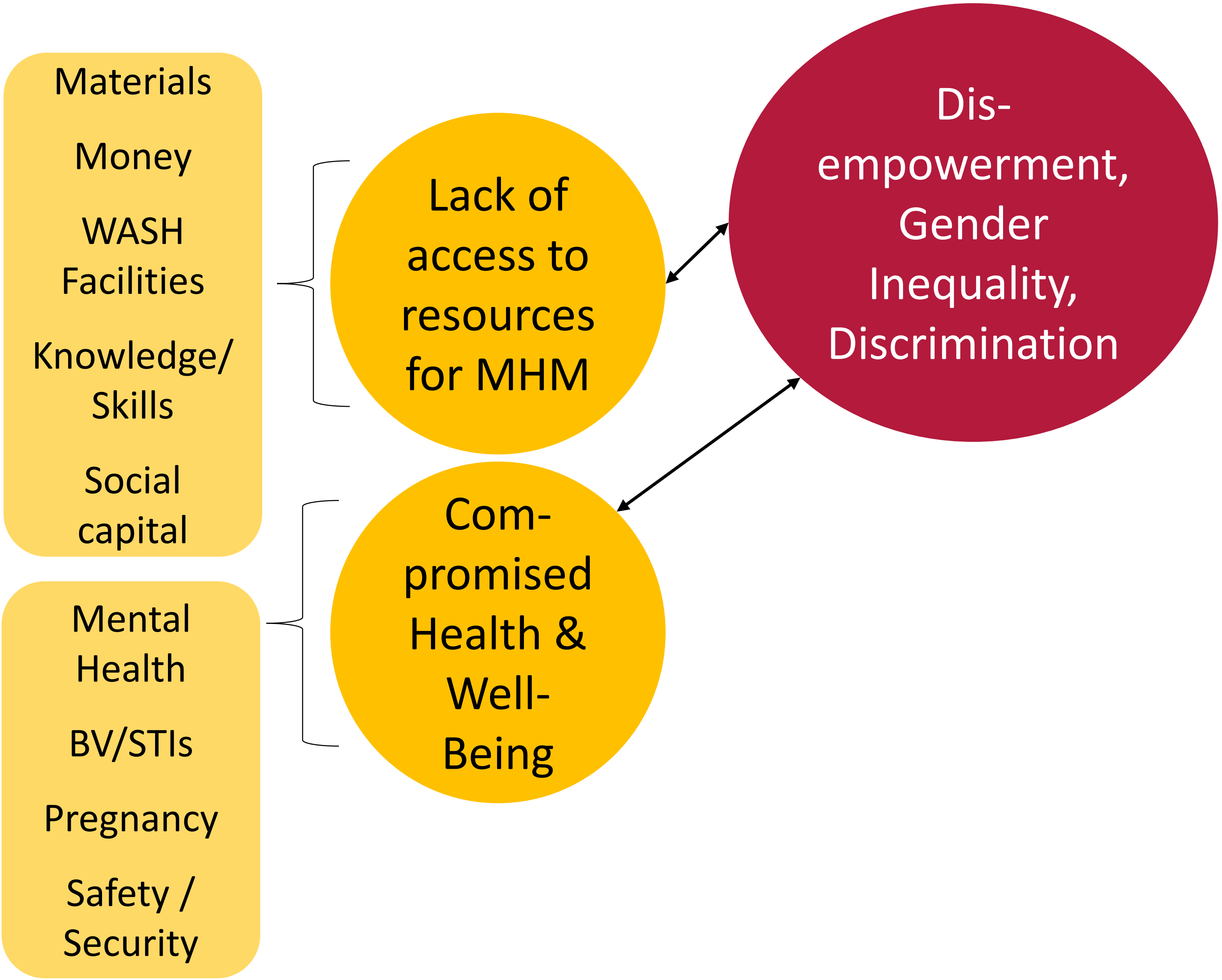
Materials
Money
WASH Facilities
Knowledge/ Skills
Social capital
Mental Health
BV/STIs
Pregnancy
Safety / Security
Lack of access to resources for MHM
Com-promised Health & Well-Being
Dis-empowerment, Gender Inequality, Discrimination

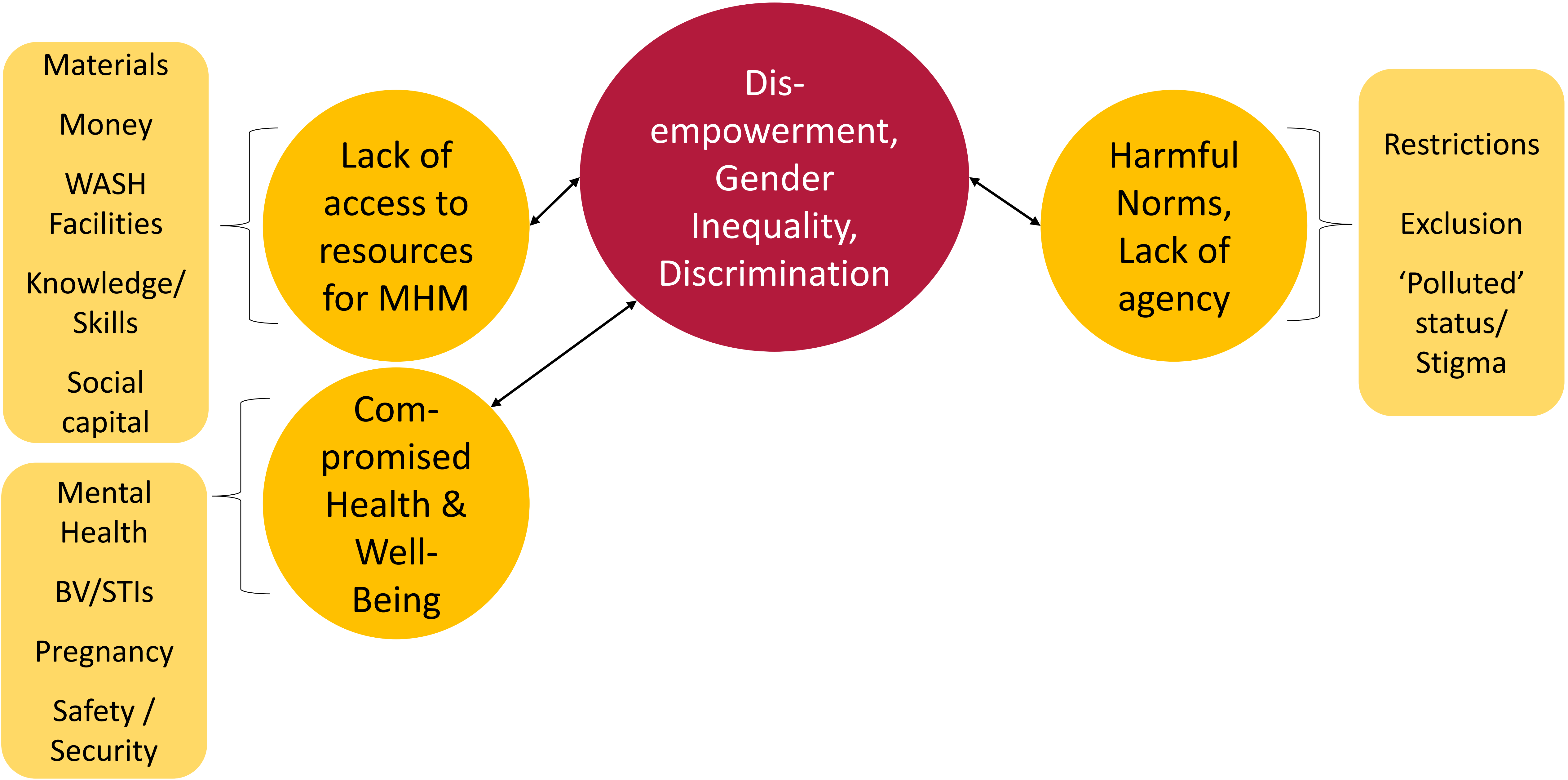
Materials
Money
WASH Facilities
Knowledge/ Skills
Social capital
Lack of access to resources for MHM
Dis-empowerment, Gender Inequality, Discrimination
Harmful Norms, Lack of agency
Restrictions
Exclusion
'Polluted' status/ Stigma
Com-promised Health & Well-Being
Mental Health
BV/STIs
Pregnancy
Safety / Security

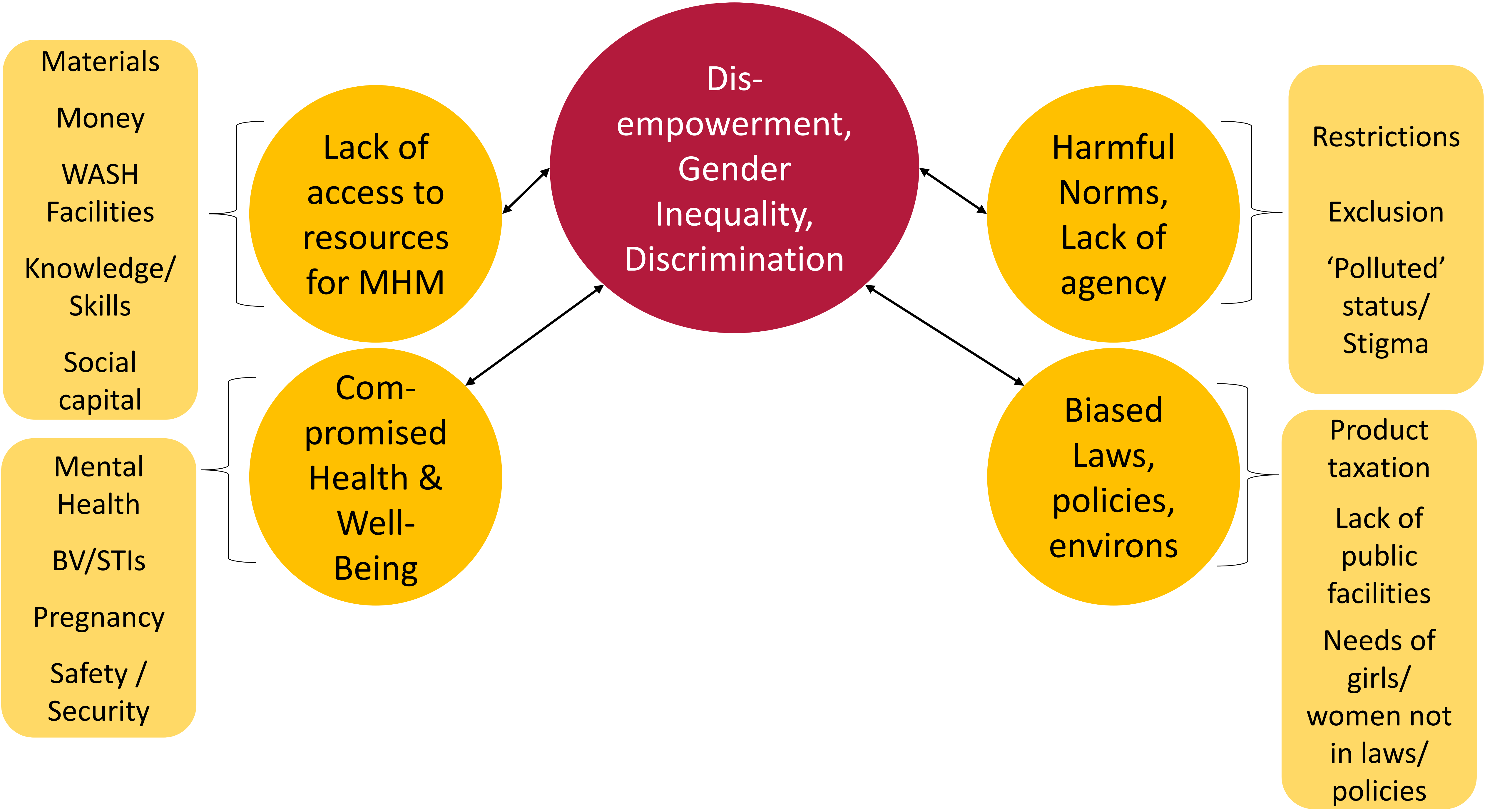
Materials
Money
WASH Facilities
Knowledge/ Skills
Social capital
Mental Health
BV/STIs
Pregnancy
Safety / Security
Lack of access to resources for MHM
Com-promised Health & Well-Being
Dis-empowerment, Gender Inequality, Discrimination
Harmful Norms, Lack of agency
Biased Laws, policies, environs
Restrictions
Exclusion
'Polluted' status/ Stigma
Product taxation
Lack of public facilities
Needs of girls/ women not in laws/ policies

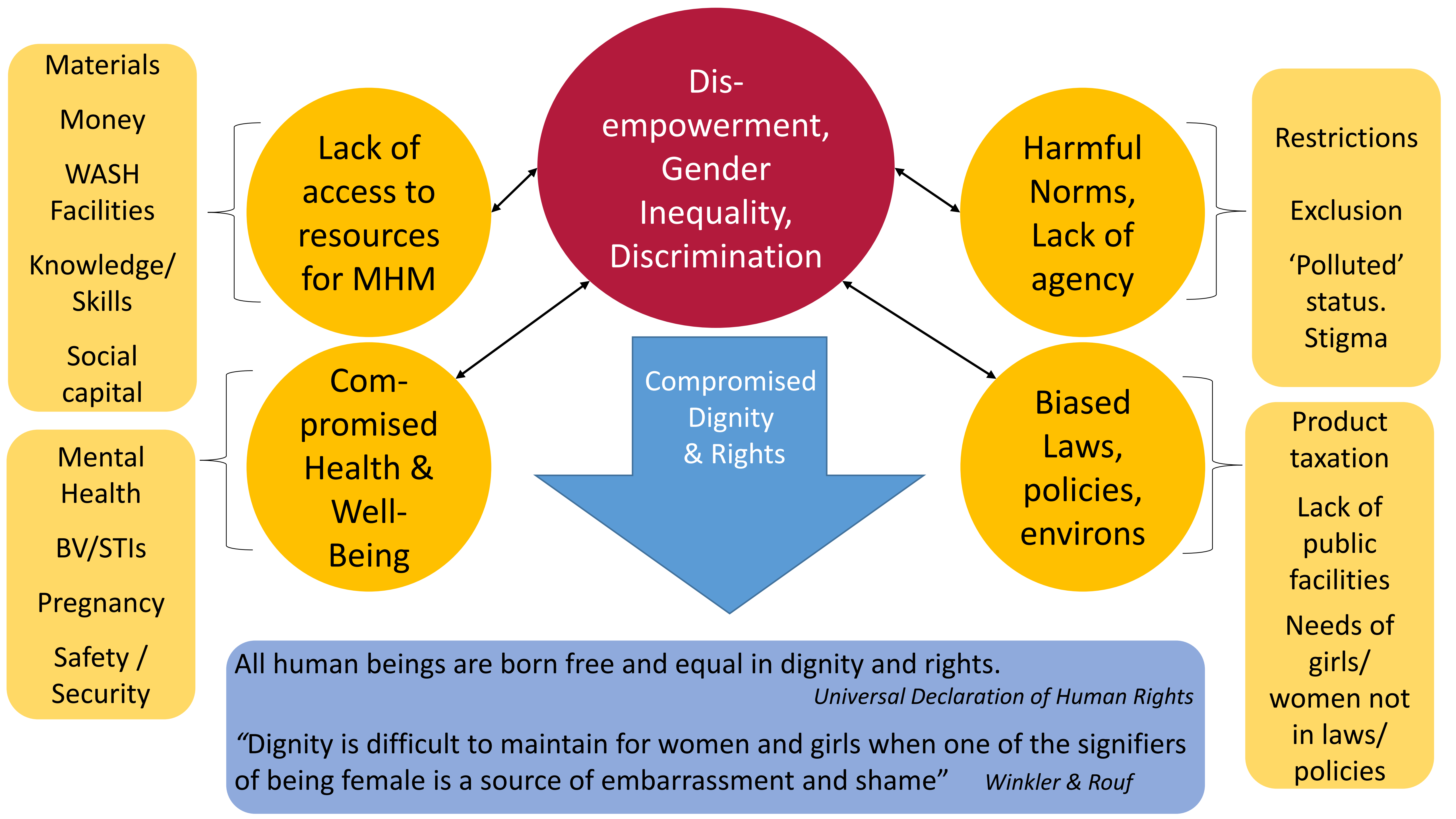
Materials
Money
WASH Facilities
Knowledge/ Skills
Social capital
Lack of access to resources for MHM
Dis-empowerment, Gender Inequality, Discrimination
Harmful Norms, Lack of agency
Restrictions
Exclusion
'Polluted' status. Stigma
Mental Health
BV/STIs
Pregnancy
Safety / Security
Com-promised Health & Well-Being
Compromised Dignity & Rights
Biased Laws, policies, environs
Product taxation
Lack of public facilities
Needs of girls/ women not in laws/ policies
All human beings are born free and equal in dignity and rights.
Universal Declaration of Human Rights
"Dignity is difficult to maintain for women and girls when one of the signifiers of being female is a source of embarrassment and shame" Winkler & Rouf

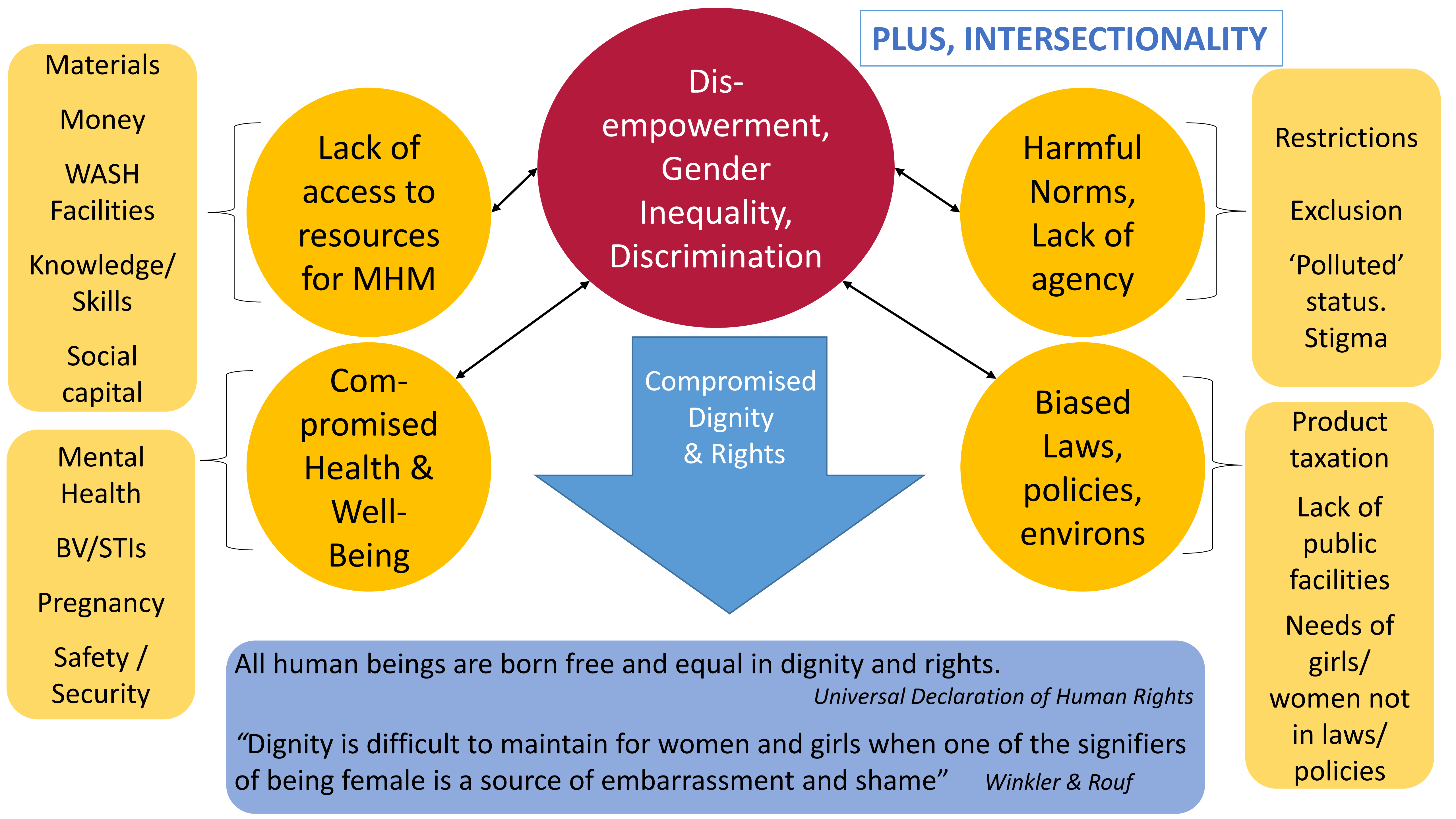
PLUS, INTERSECTIONALITY
Materials
Money
WASH Facilities
Knowledge/ Skills
Social capital
Lack of access to resources for MHM
Dis-empowerment, Gender Inequality, Discrimination
Harmful Norms, Lack of agency
Restrictions
Exclusion
'Polluted' status. Stigma
Mental Health
BV/STIs
Pregnancy
Safety / Security
Com-promised Health & Well-Being
Compromised Dignity & Rights
Biased Laws, policies, environs
Product taxation
Lack of public facilities
Needs of girls/ women not in laws/ policies
All human beings are born free and equal in dignity and rights.
Universal Declaration of Human Rights
"Dignity is difficult to maintain for women and girls when one of the signifiers of being female is a source of embarrassment and shame" Winkler & Rouf

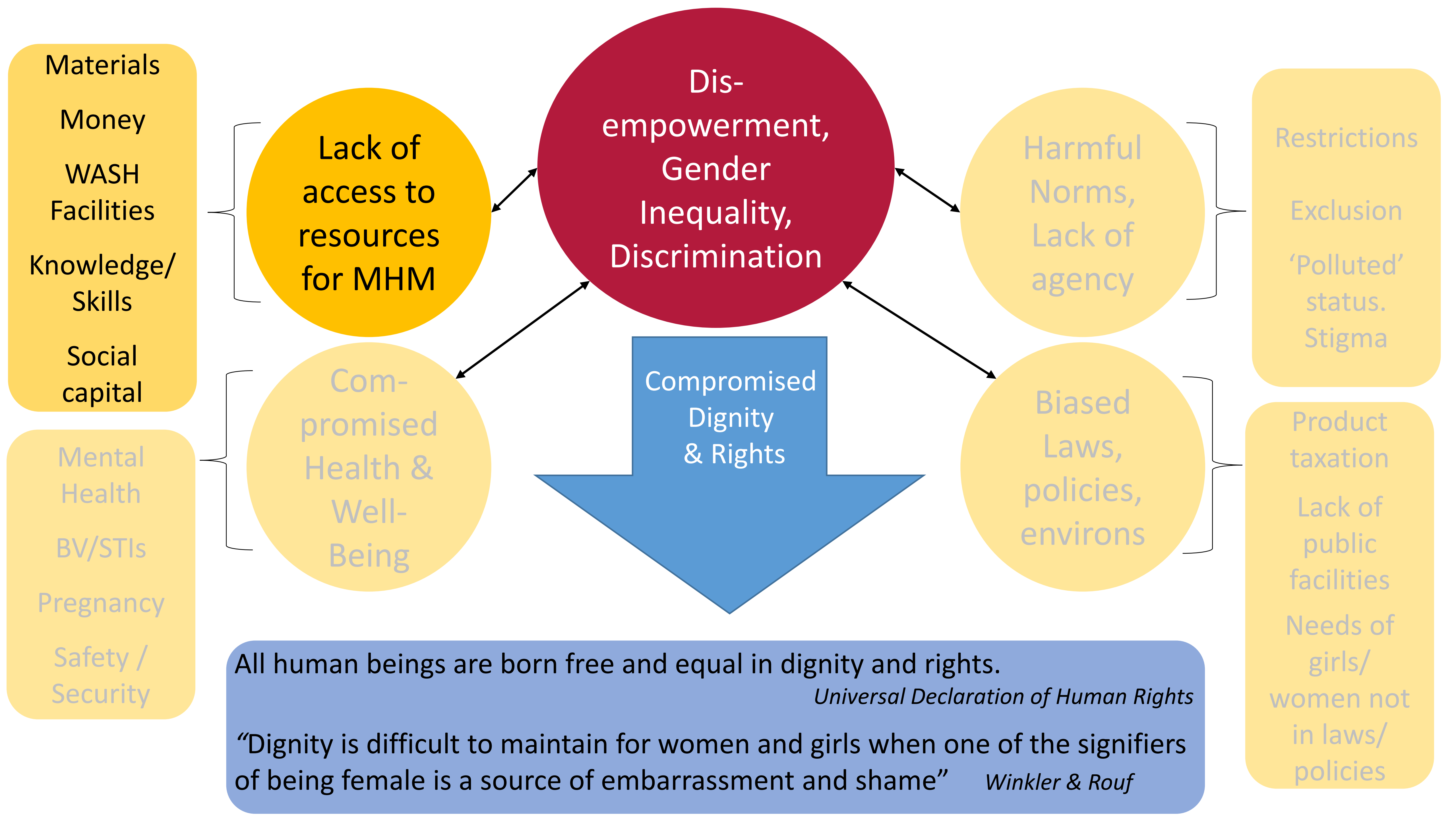
Materials
Money
WASH Facilities
Knowledge/ Skills
Social capital
Lack of access to resources for MHM
Dis-empowerment, Gender Inequality, Discrimination
Harmful Norms, Lack of agency
Restrictions
Exclusion
'Polluted' status. Stigma
Mental Health
BV/STIs
Pregnancy
Safety / Security
Com-promised Health & Well-Being
Compromised Dignity & Rights
Biased Laws, policies, environs
Product taxation
Lack of public facilities
Needs of girls/ women not in laws/ policies
All human beings are born free and equal in dignity and rights.
Universal Declaration of Human Rights
"Dignity is difficult to maintain for women and girls when one of the signifiers of being female is a source of embarrassment and shame" Winkler & Rouf

Example 1: Lack of access to resources is both an example of inequity and leads to inequity

Lack of Adequate …

WASH Facilities

Materials

Practical knowledge

Money

→

Inability to change materials at school during menstruation

Example 1: Lack of access to resources is both an example of inequity and leads to inequity

Lack of Adequate …

WASH Facilities

Materials

Practical knowledge

Money

→ Inability to change materials at school during menstruation

→ Concern/fear about leaks/stains

→ Distraction, reduced engagement, participation

→ Impacts on schooling experience,

advancement

Girls appear to do better … grades being only worse than boys around the menarche. Girls perform so well when they are in the lower primary, but when they reach class 5/6, they drop …you now find boys performing much better than girls.”

(Kenya, school teacher, Jewitt and Ryley, 2014)

Example 1: Lack of access to resources is both an example of inequity and leads to inequity

Lack of Adequate …

WASH Facilities

Materials

Practical knowledge

Money

→ Inability to change materials at school during menstruation

→ Concern/fear about leaks/stains → Distraction, reduced engagement, participation → Impacts on schooling experience, advancement

→ Leaving school to manage
Not Attending → Missed class
Missed school days
Non-advancement → Impacts on schooling experience, advancement

There is no bathroom, we usually go far over there, under the bushes. That is the reason we're late sometimes, the teachers lock the door on us.

(Bolivia, girl, Long, 2013)

Example 1: Lack of access to resources is both an example of inequity and leads to inequity

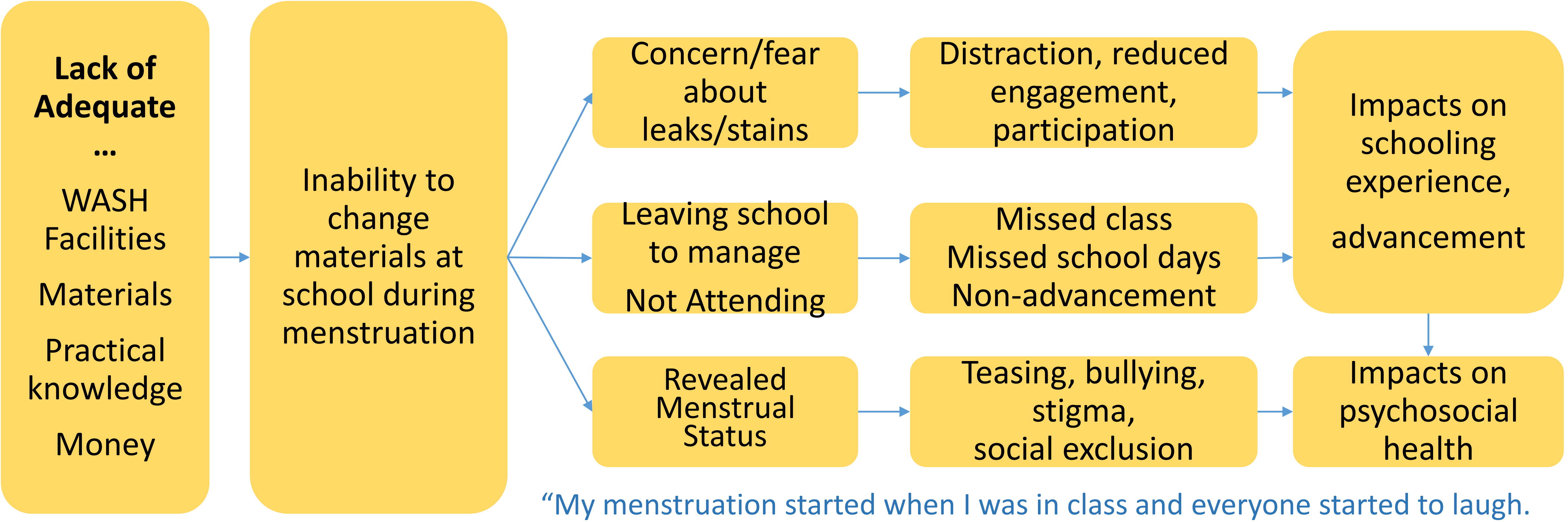

"My menstruation started when I was in class and everyone started to laugh. This is the reason that I stopped going to school."

(Ethiopia, rural out-of-school girl, Sommer et al., 2015)

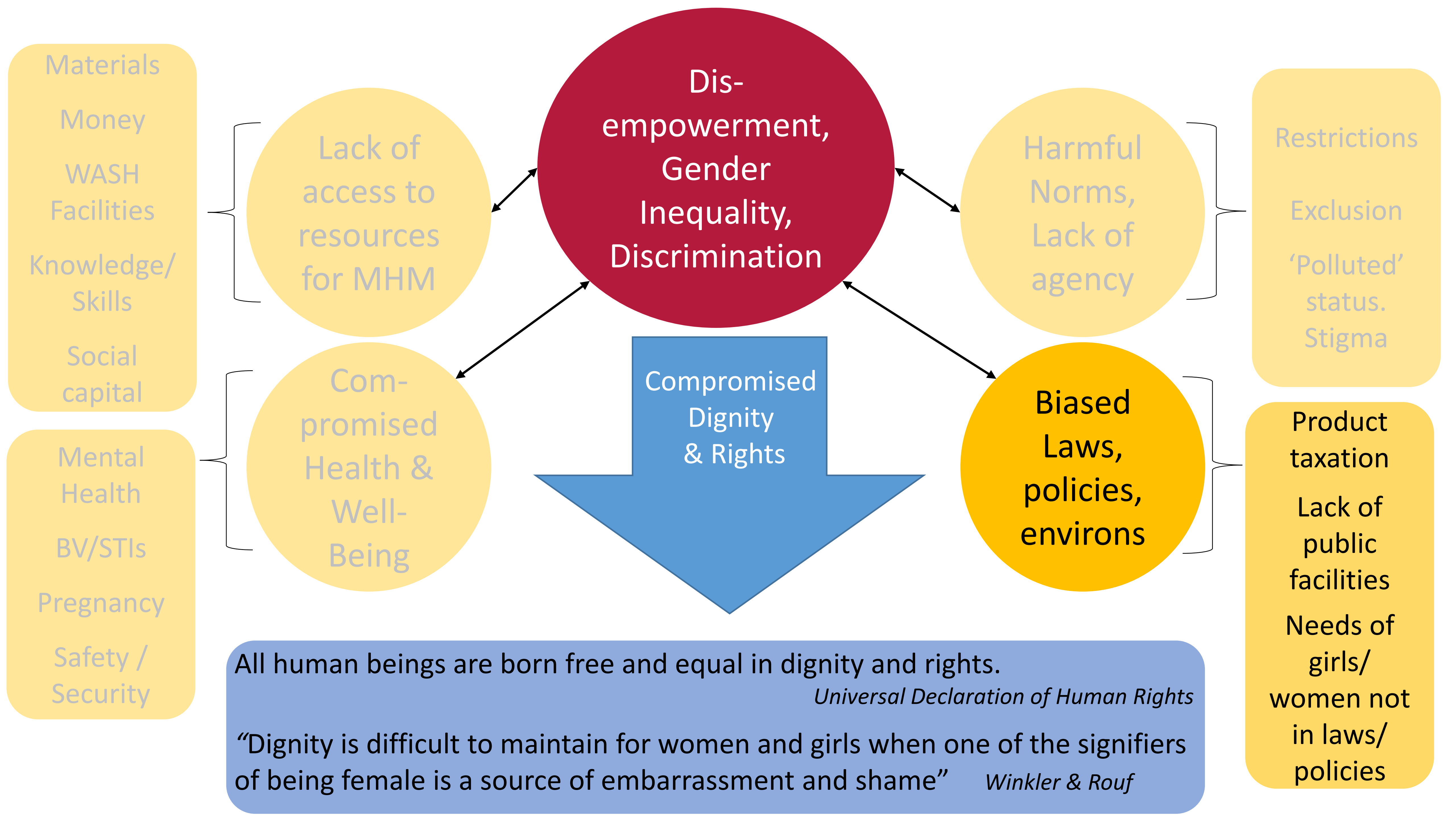
Materials
Money
WASH Facilities
Knowledge/ Skills
Social capital
Mental Health
BV/STIs
Pregnancy
Safety / Security
Lack of access to resources for MHM
Com-promised Health & Well-Being
Dis-empowerment, Gender Inequality, Discrimination
Compromised Dignity & Rights
Harmful Norms, Lack of agency
Biased Laws, policies, environs
Restrictions
Exclusion
'Polluted' status. Stigma
Product taxation
Lack of public facilities
Needs of girls/ women not in laws/ policies
All human beings are born free and equal in dignity and rights.
Universal Declaration of Human Rights
"Dignity is difficult to maintain for women and girls when one of the signifiers of being female is a source of embarrassment and shame" Winkler & Rouf

Example 2:
Discriminatory Laws/Policies Can Impact Girls

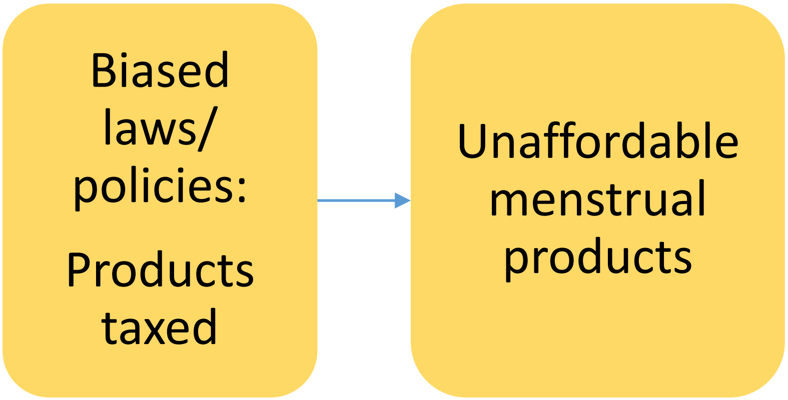

Example 2:
Discriminatory Laws/Policies Can Impact Girls

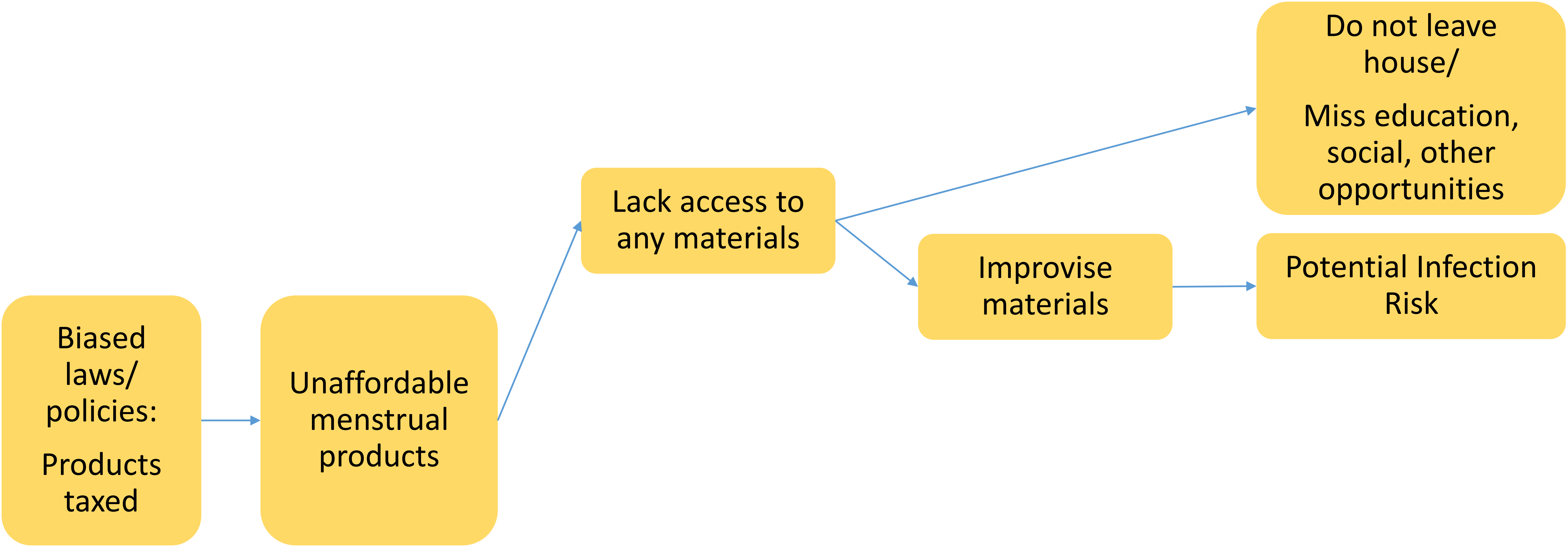

Example 2:
Discriminatory Laws/Policies Can Impact Girls

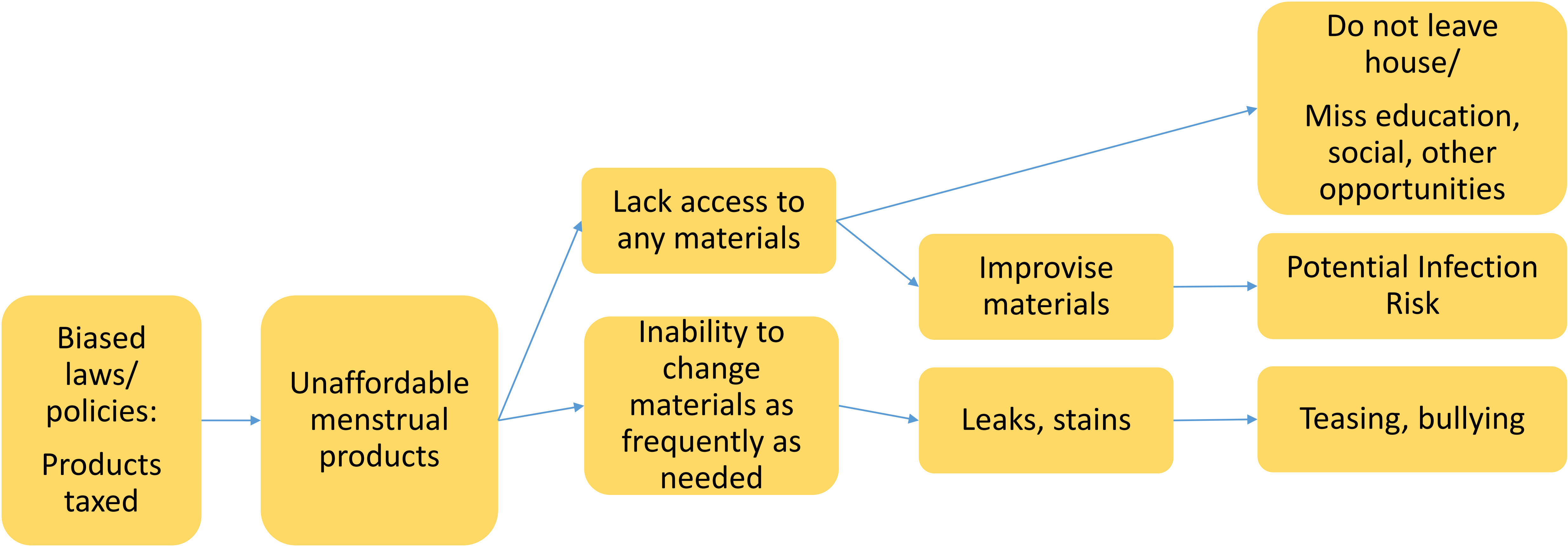

Example 2:
Discriminatory Laws/Policies Can Impact Girls

Some people exchange sex for money. The money is used to buy pads.
(Kenya, girl pupil, Mason et al., 2013)

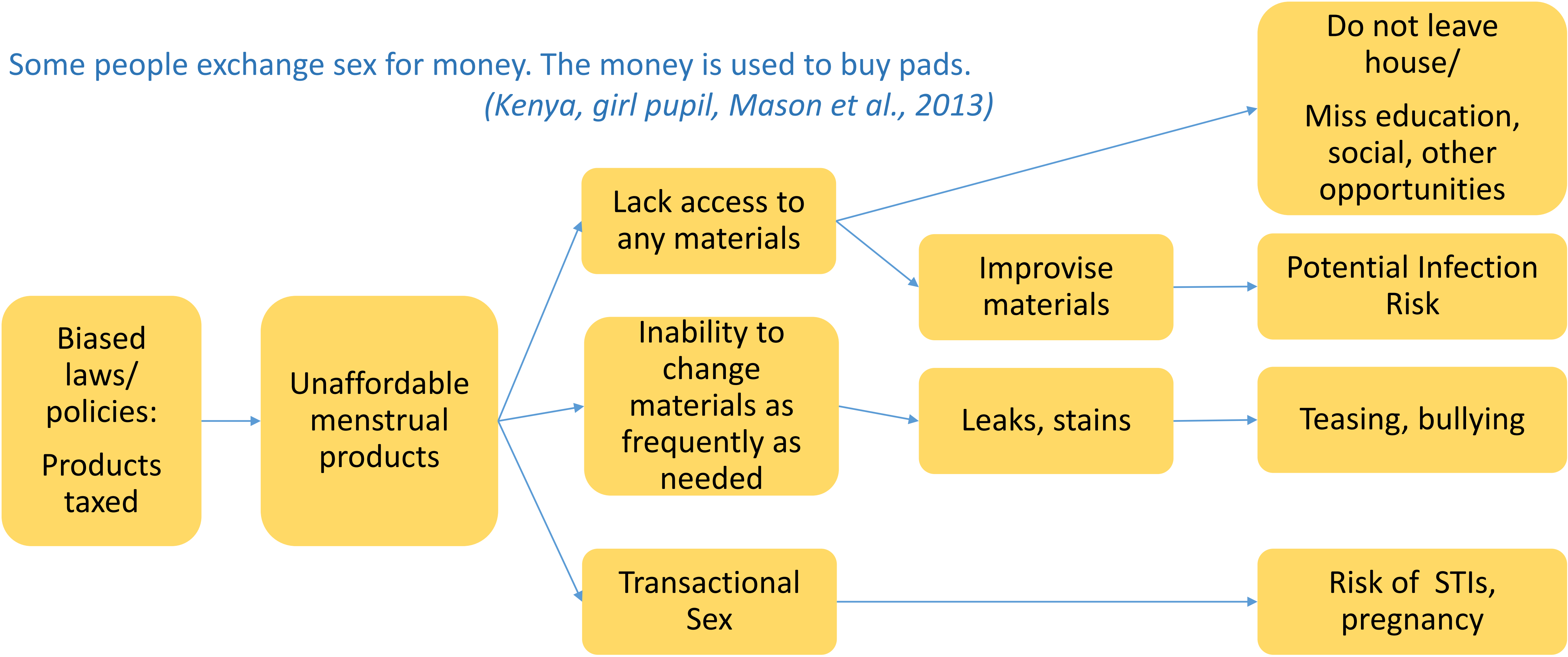

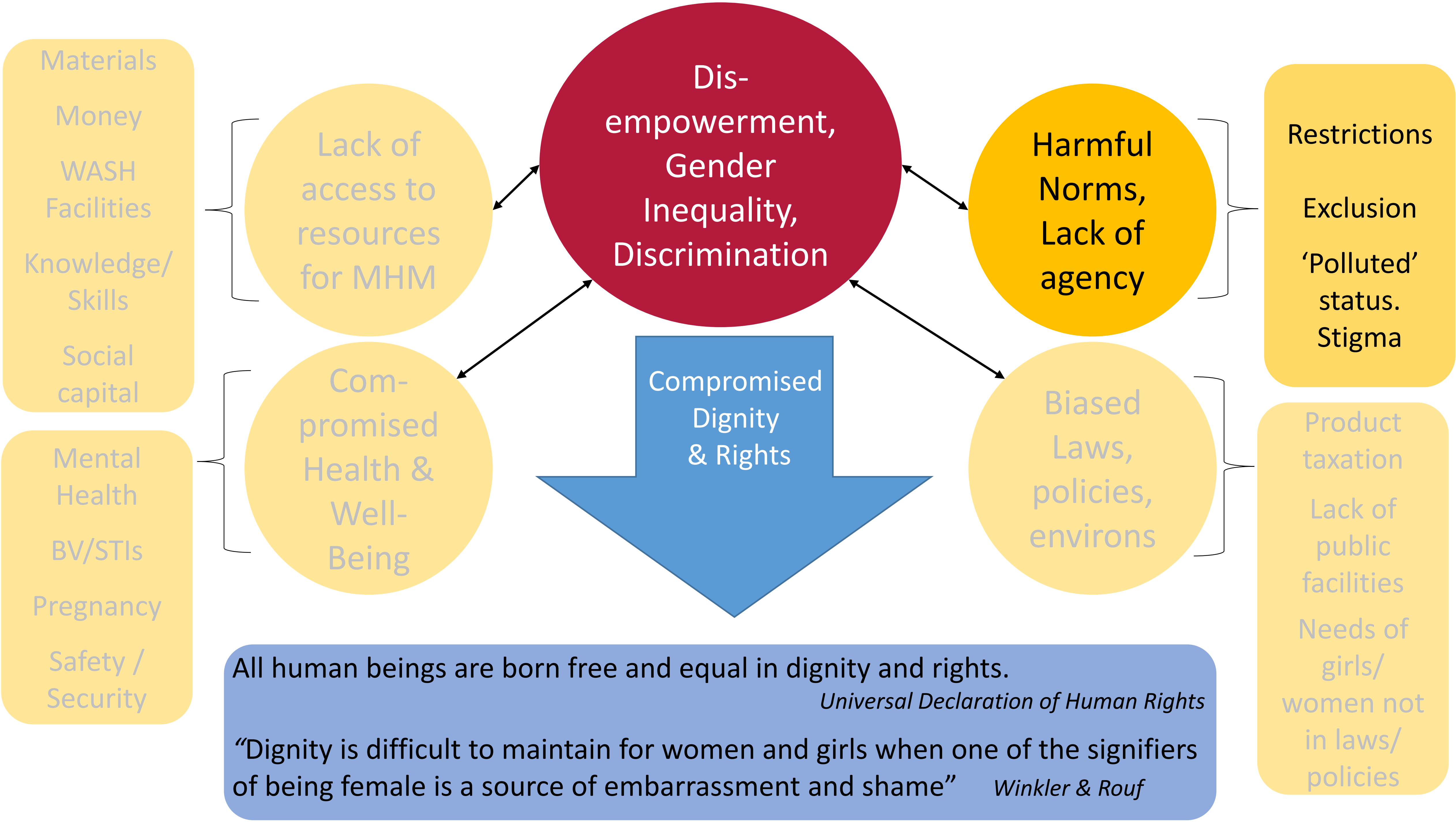
Materials
Money
WASH Facilities
Knowledge/ Skills
Social capital
Mental Health
BV/STIs
Pregnancy
Safety / Security
Lack of access to resources for MHM
Com-promised Health & Well-Being
Dis-empowerment, Gender Inequality, Discrimination
Compromised Dignity & Rights
Harmful Norms, Lack of agency
Biased Laws, policies, environs
Restrictions
Exclusion
'Polluted' status.
Stigma
Product taxation
Lack of public facilities
Needs of girls/ women not in laws/ policies
All human beings are born free and equal in dignity and rights.
Universal Declaration of Human Rights
"Dignity is difficult to maintain for women and girls when one of the signifiers of being female is a source of embarrassment and shame" *Winkler & Rouf*

Norms are Changeable

"If you tell **people they will gossip** about it in town. It will make you ashamed."
(Mali, rural school girl, Trinies et al., 2015)

"When I first started menstruating I was shocked because I had not learned about it before . . . I was too embarrassed to tell my parents because **I knew that they would not accept me** and would say that I had bad behaviour. I could not tell them because **they would say that I shamed the family and would shout at me**."
(Ethiopia, rural out-of-school girl, Sommer et al., 2015)

"I have a good male friend who I've known since childhood. One day in class I stood up and he noticed that my pants were stained and warned me about that. **I was very grateful because he understood my situation. So, boys should know about girls' puberty so they understand us better instead of making jokes**."
Girl Student, Kyrgyzstan

‘When schools ignore MHM, they are not responding to the education needs of adolescent girls. Full stop.’

- Nora Fyles

When society ignores MHM, it is not responding to the needs of adolescent girls to enable them to thrive.
